# Global antibiotic prescription practices in hospitals and associated factors: a systematic review and meta-analysis

**DOI:** 10.7189/jogh.15.04023

**Published:** 2025-01-31

**Authors:** Rui Chen, Jinxi Li, Chan Wang, Pengfei Zhou, Qihua Song, Jianxiong Wu, Qinnan Li, Hui Li, Yanhong Gong, Tao Zeng, Yu Fang, Xiaoxv Yin

**Affiliations:** 1 Department of Social Medicine and Health Management, School of Public Health, Tongji Medical College, Huazhong University of Science and Technology, Wuhan, Hubei, China; 2 School of Public Health, Tongji Medical College, Huazhong University of Science and Technology, Wuhan, Hubei, China; 3 School of Health Services Management, Anhui Medical University, Hefei, Anhui, China; 4 College of Medicine, Jingchu University of Technology, Jingmen, Hubei, China; 5 Department of Pharmacy Administration and Clinical Pharmacy, School of Pharmacy, Xi’an Jiaotong University, Xi’an, Shaanxi, China; 6 Center for Drug Safety and Policy Research, Xi’an Jiaotong University, Xi’an, Shaanxi, China

## Abstract

**Background:**

The prevalence of antibiotic prescribing among total prescriptions, the percentage of combined antibiotic prescribing among prescriptions containing at least one antibiotic, and factors influencing hospital antibiotic prescribing are currently unknown. In this systematic review, we aimed to summarise antibiotic prescribing in hospitals worldwide and identify the associated factors.

**Methods:**

We searched PubMed/MEDLINE, Ovid/Embase, and the Web of Science for articles published between 1 January 2000 and 28 February 2023 that reported antibiotic prescribing in hospitals or the associated factors. Four reviewers independently screened studies, extracted data, and assessed the risk of bias. We used meta-analysis with random-effects models to estimate the pooled effect sizes.

**Results:**

We included 403 studies covering 93 economies. The pooled prevalence of antibiotic prescribing among total prescriptions was 34.3% (95% CI = 29.6–39.3) in outpatient settings and 47.7% (95% CI = 45.8–49.5) in inpatient settings. The pooled percentages of antibiotics in the ‘access’ group were 48.5% (95% CI = 34.5–62.7) in outpatient settings and 43.8% (95% CI = 39.2–48.5) in inpatient settings. Subgroup analysis showed the prevalence of antibiotic prescribing was significantly higher in low-income compared to high-income economies. Additionally, there was a rising trend of the prevalence in inpatient settings over time. The studies showed that patients’ gender, education level, health status, and physicians’ work experience were associated with hospital antibiotic prescribing.

**Conclusions:**

The global prevalence of antibiotic prescribing in hospitals is high, with significant disparities across regions. Multifaceted measures with multi-sectoral cooperation are required, such as regulatory interventions, professional training for physicians, and public health education.

**Registration:**

PROSPERO: CRD42022354076.

Antimicrobial resistance (AMR) has become a global health problem [1]. It was associated with approximately 4.95 million deaths worldwide in 2019, of which 1.27 million were directly attributable [2]. As antibiotic consumption is an essential driver behind this phenomenon [3,4], promoting their rational use is a key strategy to control, which is one of the objectives of the Global Action Plan instituted at the World Health Assembly in 2015 [5,6].

Since hospitals are one of the main sources of antibiotic prescribing, monitoring their practices is a key tool in optimising antimicrobial stewardship and controlling AMR by managing trends in antibiotic use [7]. Consequently, the prevalence of antibiotic use was recommended by the World Health Organization (WHO) as the major indicator for surveillance [8]. Yet due to the imbalanced development of health service systems across countries, a global standardised surveillance network has not been established, resulting in lacking global reports on the prevalence of hospital antibiotic prescribing. Numerous studies conducted in the past two decades have suggested that the prevalence of antibiotic use in hospitals differs between nations, ranging from 19.5% in outpatient settings in the USA [9] to 53.8% in Ethiopia [10]. In inpatient settings, the prevalence of antibiotic prescribing was found to be 23.1% in France [11] and 82.3% in Pakistan [12]. Studies from such diverse contexts provide the basis for monitoring worldwide hospital antibiotic prescription practices, providing an opportunity for conducting a comprehensive analysis of national data to evaluate the global situation and to identify weaknesses in management. One systematic review from 2020 assessed antibiotic prescriptions in hospitals worldwide [13]. However, it only included 60 studies and did not perform a quantitative analysis, leaving a need for more accurate data.

Evaluating the factors associated with hospital antibiotic prescribing could provide an evidence base for the adjustment of antimicrobial stewardship. Several studies showed that this practice is influenced by multiple factors, such as patients’ education levels, symptom duration, and physicians’ work experience [14–16]. However, the associated factors were not consistent among studies, and few quantitative reviews elaborated on the strengths of these associations. Therefore, further research in this area is needed.

Considering the inadequacy of worldwide representative evidence, we conducted a systematic review to assess the antibiotic prescribing pattern of global hospitals and associated factors, which could provide the latest evidence to support antimicrobial stewardship.

## METHODS

We registered the protocol for this systematic review in PROSPERO (ID: CRD42022354076) and reported it according to PRISMA guidelines [17] (Table S1 in the **Online Supplementary Document**).

### Data sources and searches

We searched PubMed/MEDLINE, Ovid/Embase, and Web of Science for literature published in English between 1 January 2000 and 28 February 2023. We combined ‘hospital’, ‘antibiotic’, ‘prescription’, ‘consumption’ and other related terms to develop a search strategy (Text S1 in the **Online Supplementary Document**). In addition, we manually searched the references of the included studies to identify additional articles we may have missed in the previous steps.

We imported the search results into the citation manager NoteExpress, version 3.7.0.9296 (Beijing Aegean Software Co., Ltd, Beijing, China) and removed duplicates. Based on pre-defined inclusion and exclusion criteria, four authors (RC, JL, QS, and PZ) independently screened the retrieved records (Text S2 in the **Online Supplementary Document**). Two authors (RC and JL) then reviewed the full-text of the remaining articles. Disagreements were resolved through discussion and, if necessary, consultation with one senior author (XY) until a consensus was reached.

### Study selection

We considered ‘hospital’ as any medical environment, except those in primary care (*e.g.* general practitioner clinics, community health centres, and rural health clinics), and ‘antibiotic’ as any agent included in the J01 group (*i.e.* antibiotics for systemic use) of the standardised WHO Anatomical Therapeutic Chemical classification system [18]. We included cross-sectional, longitudinal, and intervention studies reporting baseline data on the indicators on hospital antibiotic prescribing or associated factors.

Original studies had to meet specific criteria for the assessment and calculation of indicators (Text S2 in the **Online Supplementary Document**). For example, we included any study that assessed the appropriateness of antibiotic prescribing based on recommended indications, duration, route of administration, spectrum, and dosage in international, local, or institutional guidelines. We excluded mathematical modelling studies, economic analyses, conference proceedings, qualitative studies, abstracts, reviews, editorials, and letters, as well as studies focussing on a single (*e.g.* ceftriaxone) or several types of antibiotics (*e.g.* third-generation cephalosporins) of the J01 group.

### Data extraction and quality assessment

Three reviewers (JL, RC, and QS) extracted the following data from the included studies into a structured sheet: bibliographic information, basic study information, study setting, and information on hospital antibiotic prescribing and the associated factors (Text S3 in the **Online Supplementary Document**). Any discrepancies were resolved through discussion.

We divided regions into seven types according to the World Bank classification criteria: Europe and Central Asia, sub-Saharan Africa, East Asia and Pacific, North America, South Asia, Middle East and North Africa, and Latin America and the Caribbean, and classified income groups of economies where the research was conducted into four categories: high income, upper middle income, lower middle income, and low income [19]. According to the WHO AWaRe classification, we divided the antibiotics into ‘access’, ‘watch’, and ‘reserve’ groups [20]. In addition, to analyse the association between the implementation of the Antimicrobial Resistance National Action Plan (AMR NAP) and antibiotic prescribing in hospitals, we divided studies into those conducted before and those conducted after the implementation of the action plan [21] (Table S2 in the **Online Supplementary Document**).

Four authors (RC, JL, QS, and PZ) used a modified version of a tool developed by Hoy [22] to evaluate the risk of bias in each included study, which included nine items: research population, sampling frame, population selecting, study excluding, data collection, case definition, antibiotic definition, and accuracy of the information on the effect size, with each item being allocated one point (Text S4 in the **Online Supplementary Document**). We then categorised the studies according to their total score as high quality (8–9 points), moderate quality (6–7 points), and low quality (1–5 points). Disagreements were resolved through discussion.

### Data synthesis and analysis

The pooled effect sizes for antibiotic prescribing were the prevalence of antibiotic prescribing among total prescriptions, the percentage of combined antibiotic prescribing among prescriptions containing at least one antibiotic, the percentage of parenterally administered antibiotic prescribing among prescriptions containing at least one antibiotic, the percentage of inappropriate antibiotic prescribing among prescriptions containing at least one antibiotic, and the percentage of antibiotic prescribing based on the AWaRe classification among prescriptions containing at least one antibiotic in outpatient or inpatient settings. We further added the percentage of empirical antibiotic prescribing and the percentage of prophylaxis antibiotic prescribing among prescriptions containing at least one antibiotic in inpatient settings (Text S5 in the **Online Supplementary Document**).

Considering the heterogeneity between studies, we used a random-effects model to estimate the pooled indicators. We used the Freeman-Tukey transformation and provided the summarised estimates after reverse transformation [23,24]. We assessed the heterogeneity between studies with *I*^2^ statistic, which indicates the proportion of total variance attributable to between-study variation. We explored the potential source of heterogeneity through subgroup analysis and meta-regression. We performed subgroup analyses according to the study location, income group, study period, patient type, hospital type, study quality, and condition of AMR NAP implementation.

The outcome variable in the meta-regression analysis was the prevalence of antibiotic prescribing in hospitals, while the explanatory variables were study characteristics that might affect the prevalence. We considered any study results stratified by country, study period, patient type, or hospital type as independent reports. Studies have shown that traditional methods, such as funnel plots and asymmetry tests, are not suitable for assessing publication bias in prevalence studies; therefore, we did not formally evaluate publication bias [25]. However, we did perform sensitivity analyses using the leave-one-out approach to assess the effect of any single study on the pooled prevalence estimates.

The effect sizes for the associated factors were the odds ratios (ORs) with 95% confidence intervals (CIs). As an example from prior studies, this included factors that were associated with antibiotic prescribing in hospitals from the perspectives of patients (*e.g.* illness condition), physicians (*e.g.* years of experience), or hospitals (*e.g.* size). We considered a variable if its OR and 95% CI were both reported at least five times in original studies. The factors analysed included the patient’s gender, age, educational level, income level, health insurance, their health status (symptom, comorbidity status, hospitalisation duration, and illness duration); physician’s gender, age, and work experience; settings (urban vs rural), and the size of facilities. We used random-effect meta-analysis models to estimate the pooled effect sizes of these factors.

We performed the analyses with STATA, version 17.0 (StataCorp, Texas, US) and R, version 4.2.1 (R Core Team, Vienna, Austria), and all statistical tests were two-sided tests with a significance level of 0.05.

## RESULTS

**Figure 1:**
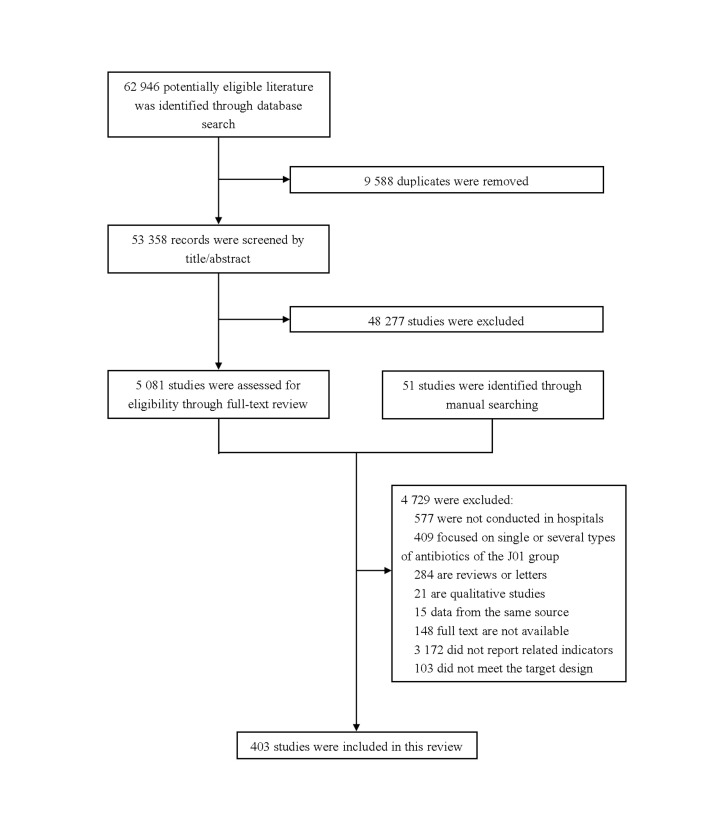
Flowchart of study selection.

We retrieved 62 946 records through systematic search and reference screening, of which 403 were included (**Figure 1**; Tables S3 and S4 in the **Online Supplementary Document**). Among them, 263 were published after 2015 (Table S5 in the **Online Supplementary Document**). The studies covered 93 economies, of which 39 (42.2%) were in the high-income group. In terms of the quality assessment, 109 (27.0%) were of high quality, 222 (55.1%) were of moderate quality, and 72 (17.9%) were of low quality (Table S6 in the **Online Supplementary Document**).

### Antibiotic prescribing in outpatient settings

We extracted 88 reports from 72 studies that reported the prevalence of antibiotic prescribing among total prescriptions in outpatient settings. The pooled prevalence was 34.3% (95% CI = 29.6–39.3) (Figures S1 and S2 in the **Online Supplementary Document**).

The prevalence of antibiotic prescribing in outpatient settings among regions differed significantly (*P* < 0.001) and was the highest in sub-Saharan Africa (51.5%; 95% CI = 43.5–59.4) and the lowest in Europe and Central Asia (15.2%; 95% CI = 14.4–16.0) (**Table 1**). The prevalence was higher in low-income economies (52.7%; 95% CI = 45.8–59.6) compared to high-income economies (17.2%; 95% CI = 12.2–22.9) (*P* < 0.001). We found no significant change in the prevalence of antibiotic prescribing in outpatient settings over time. The results of meta-regression indicated significant associations of the prevalence of antibiotic prescribing in outpatient settings with the region (*P* < 0.001), income group (*P* < 0.001), and patient type (*P* = 0.012), but not with the year of study (*P* = 0.110), hospital type (*P* = 0.380), study quality (*P* = 0.540), and implementation of AMR NAP (*P* = 0.760). The sensitivity analysis showed that the pooled prevalence was robust and not dependent on a single report. After a study was omitted, the prevalence fluctuated between 33.7% (95% CI = 29.1–38.6) and 34.8% (95% CI = 30.0–39.7).

**Table 1:** Subgroup analyses of the prevalence of outpatient antibiotic prescribing

	Reports, n	Prevalence, % (95% CI)	*I* ^2^	*P*-value for heterogeneity	*P*-value between groups
**Total**	88	34.3 (29.6–39.3)	>99.9	<0.001	
**Region**					<0.001
Sub-Saharan Africa	29	51.5 (43.5–59.4)	99.5	<0.001	
South Asia	19	37.2 (27.8–47.1)	99.6	<0.001	
Middle East and North Africa	6	33.4 (12.9–57.9)	>99.9	<0.001	
East Asia and Pacific	26	20.7 (16.1–25.7)	>99.9	<0.001	
North America	5	19.0 (10.5–29.2)	>99.9	<0.001	
Europe and Central Asia	3	15.2 (14.4–16.0)	19.5	0.290	
**Income group**					<0.001
Low income	22	52.7 (45.8–59.6)	99.1	<0.001	
Lower middle income	26	40.9 (31.5–50.6)	99.6	<0.001	
Upper middle income	26	23.6 (17.5–30.4)	>99.9	<0.001	
High income	14	17.2 (12.2–22.9)	>99.9	<0.001	
**Year of study**					0.110
2010 and before	17	43.5 (33.8–53.5)	99.9	<0.001	
2011–15	32	35.8 (27.2–44.9)	>99.9	<0.001	
After 2015	39	29.4 (23.0–36.1)	>99.9	<0.001	
**Research population**					0.012
Adults	69	31.1 (26.2–36.2)	>99.9	<0.001	
Children	19	46.5 (34.8–58.4)	99.9	<0.001	
**Hospital type**					0.380
Public	34	33.7 (26.2–41.6)	>99.9	<0.001	
Private	3	53.5 (40.4–66.3)	93.5	<0.001	
Not specified	51	33.7 (27.4–40.3)	99.9	<0.001	
**Study quality**					0.540
High	21	34.7 (25.2–44.8)	>99.9	<0.001	
Moderate	57	33.1 (27.1–39.5)	99.9	<0.001	
Low	9	40.5 (29.4–52.0)	99.9	<0.001	
**Implementation of AMR NAP**					0.760
Yes	15	32.7 (20.5–46.1)	>99.9	<0.001	
No	73	34.7 (29.5–40.0)	>99.9	<0.001	

AMP NAP – Antimicrobial Resistance National Action Plan, CI – confidence interval

In outpatient settings, 24 reports mentioned the percentage of combined antibiotic prescribing among prescriptions containing at least one antibiotic (14.7%; 95% CI = 11.4–18.2), 12 mentioned the percentage of parenterally administered antibiotic prescribing among prescriptions containing at least one antibiotic (17.4%; 95% CI = 7.0–31.2), and 14 mentioned the percentage of inappropriate antibiotic prescribing among prescriptions containing at least one antibiotic (26%; 95% CI = 13.8–40.7) (Table S7 in the **Online Supplementary Document**). Forty-two reports mentioned the percentage of antibiotic prescribing based on the AWaRe classification among prescriptions containing at least one antibiotic, with a percentage of 48.5% (95% CI = 34.5–62.7) in the ‘access’ group (Tables S8 and S9 in the **Online Supplementary Document**).

### Antibiotic prescribing in inpatient settings

We extracted 439 reports from 334 studies that reported the prevalence of antibiotic prescribing among total prescriptions in inpatient settings, and the pooled prevalence was 47.7% (95% CI = 45.8–49.5) (Figure S3 in the **Online Supplementary Document**).

The prevalence of antibiotic prescribing in inpatient settings among regions was significantly different (*P* < 0.001), with the highest in South Asia (68.9%; 95% CI = 60.9–76.3) and the lowest in Europe and Central Asia (37.0%; 95% CI = 35.1–39.0) (**Table 2**). The prevalence was significantly higher in low-income economies (67.5%; 95% CI = 61.1–73.6) compared to high-income economies (37.4%; 95% CI = 35.9–39.1, *P* < 0.001). Additionally, there was a significant rising trend in the prevalence of inpatient antibiotic prescribing over time, with the highest point being after 2015 (51.9%; 95% CI = 49.0–54.7) and the lowest in 2010 and before (41.0%; 95% CI = 37.6–44.5). The results of meta-regression indicated significant associations of the prevalence of antibiotic prescribing in inpatient settings with the region (*P* < 0.001), income group (*P* < 0.001), year of study (*P* < 0.001), patient type (*P* < 0.001), and hospital type (*P* = 0.008), but not with study quality (*P* = 0.680) and the implementation of AMR NAP (*P* = 0.061). The sensitivity analysis showed that the pooled prevalence was robust and not dependent on a single report. After a study was omitted, the prevalence fluctuated between 47.5% (95% CI = 45.7–49.3) and 47.8% (95% CI = 45.9–49.6).

**Table 2:** Subgroup analyses of the prevalence of inpatient antibiotic prescribing

	Reports, n	Prevalence, % (95% CI)	*I* ^2^	*P*-value for heterogeneity	*P*-value between groups
**Total**	439	47.7 (45.8–49.5)	99.9	<0.001	
**Region**					<0.001
South Asia	29	68.9 (60.9–76.3)	99.6	<0.001	
Sub-Saharan Africa	71	63.2 (58.7–67.7)	98.9	<0.001	
Middle East and North Africa	18	57.7 (49.0–66.2)	98.9	<0.001	
East Asia and Pacific	90	49.3 (45.4–53.3)	>99.9	<0.001	
Latin America and the Caribbean	22	47.2 (41.1–53.4)	98.8	<0.001	
North America	24	43.8 (40.0–47.6)	99.9	<0.001	
Europe and Central Asia	181	37.0 (35.1–39.0)	99.5	<0.001	
Not specified	4	42.6 (33.5–52.0)	99.9	<0.001	
**Income group**					<0.001
Low income	37	67.5 (61.1–73.6)	98.8	<0.001	
Lower middle income	73	65.2 (60.9–69.3)	99.6	<0.001	
Upper middle income	113	49.3 (45.7–52.9)	99.9	<0.001	
High income	206	37.4 (35.9–39.1)	99.9	<0.001	
Not specified	10	38.6 (33.5–43.9)	99.8	<0.001	
**Year of study**					<0.001
2010 and before	110	41.0 (37.6–44.5)	>99.9	<0.001	
2011–2015	128	46.8 (43.6–50.0)	99.8	<0.001	
After 2015	201	51.9 (49.0–54.7)	99.8	<0.001	
**Research population**					<0.001
Adults	304	45.1 (43.1–47.0)	99.9	<0.001	
Children	135	53.6 (49.5–57.6)	99.8	<0.001	
Hospital type					0.008
Public	102	52.3 (48.4–56.3)	>99.9	<0.001	
Private	17	53.9 (41.5–66.0)	99.9	<0.001	
Not specified	320	45.8 (43.7–48.0)	99.8	<0.001	
**Study quality**					0.680
High	125	48.4 (45.1–51.7)	99.8	<0.001	
Moderate	276	47.8 (45.5–50.1)	99.9	<0.001	
Low	38	44.2 (35.5–53.1)	99.6	<0.001	
**Implementation of AMP NAP**					0.061
Yes	35	55.3 (47.9–62.5)	99.6	<0.001	
No	402	47.0 (45.0–48.9)	99.9	<0.001	
No data report	2	52.6 (43.8–61.3)	69.9	<0.001	

AMP NAP – Antimicrobial Resistance National Action Plan, CI – confidence interval

**Figure 2:**
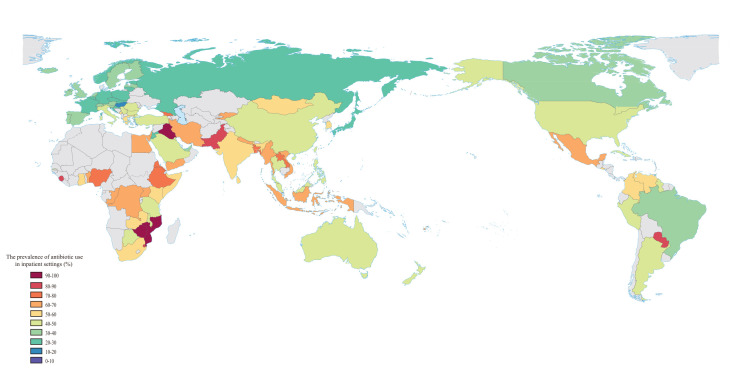
Inpatient antibiotic prescribing across countries.

The prevalence of antibiotic prescribing in inpatient settings was higher in countries in sub-Saharan Africa, South Asia, and Middle East and North Africa (**Figure 2**). We analysed trends of the prevalence of antibiotic prescribing in nine countries covered by at least five original studies and found no significant decline over the periods covered in most cases (Figure S4 in the **Online Supplementary Document**).

In inpatient settings, 149 reports mentioned the percentage of combined antibiotic prescribing among prescriptions containing at least one antibiotic (38.4%; 95% CI = 35.6–41.3), 56 mentioned the percentage of empirical antibiotic prescribing among prescriptions containing at least one antibiotic (73.7%; 95% CI = 68.0–79.1), 120 mentioned the percentage of prophylaxis antibiotic prescribing among prescriptions containing at least one antibiotic (32.1%; 95% CI = 29.6–34.8), 114 mentioned the percentage of parenterally administered antibiotic prescribing among prescriptions containing at least one antibiotic (76.2%; 95% CI = 73.4–78.8), and 53 mentioned the percentage of inappropriate antibiotic prescribing among prescriptions containing at least one antibiotic (37.8%; 95% CI = 33.8–41.9) (Table S7 in the **Online Supplementary Document**). Additionally, 195 reports mentioned the percentage of antibiotic prescribing based on the AWaRe classification among prescriptions containing at least one antibiotic, with a percentage of 43.8% (95% CI = 39.2–48.5) in the ‘access’ group (Tables S8 and S9 in the **Online Supplementary Document**).

### Factors associated with antibiotic prescribing in hospitals

Seventy-four studies reported data on factors associated with hospital antibiotic prescribing (Table S10 in the **Online Supplementary Document**). Among the reported factors, a higher educational level of the patient (OR = 0.922; 95% CI = 0.865–0.982) was found to reduce hospital antibiotic prescribing (**Table 3**). Male patients (OR = 1.054; 95% CI = 1.011–1.099), patients with fever (OR = 1.173; 95% CI = 1.139–1.209), patients with a comorbidity (OR = 1.168; 95% CI = 1.035–1.318), patients with longer illness duration (OR = 1.178; 95% CI = 1.198–2.465), and physicians with longer work experience (OR = 1.297; 95% CI = 1.176–1.431) were found to be associated with higher antibiotic prescribing in hospitals.

**Table 3:** Factors associated with antibiotic prescribing in hospitals

	Number of reports	OR (95% CI)	*I* ^2^	*P* value for heterogeneity
Patient’s age (older vs younger)	143	0.964 (0.893–1.041)	99.9	<0.001
Patient’s gender (male vs female)	61	1.054 (1.011–1.099)	98.8	<0.001
Patient’s ducational level (higher vs lower)	7	0.922 (0.865–0.982)	23.4	0.006
Patient’s income level (higher vs lower)	6	1.048 (0.985–1.115)	65.0	0.007
Symptom (fever or not)	27	1.173 (1.139–1.209)	95.8	<0.001
Symptom (cough or not)	5	1.421 (0.835–2.419)	95.2	<0.001
Comorbidity status (yes vs no)	9	1.168 (1.035–1.318)	95.8	<0.001
Hospitalisation duration (longer vs shorter)	8	0.905 (0.672–1.220)	94.3	<0.001
Illness duration (longer vs shorter)	6	1.718 (1.198–2.465)	94.7	<0.001
Health insurance (have vs not have)	5	1.179 (0.868–1.600)	89.1	<0.001
Physician’s age (older vs younger)	12	1.101 (0.903–1.130)	92.4	<0.001
Physician’s gender (male vs female)	7	1.158 (0.952–1.407)	88.3	0.002
Physician’s work experience (longer vs shorter)	18	1.297 (1.176–1.431)	92.8	<0.001
Settings (urban vs rural)	10	0.870 (0.705–1.075)	82.9	<0.001
Size of facilities (larger vs smaller)	11	0.843 (0.700–1.014)	96.9	<0.001

OR – odds ratio, CI – confidence interval

## DISCUSSION

This study provides a comprehensive overview of global antibiotic prescribing patterns in outpatient and inpatient settings, including indicators reflecting the pattern and identifying its associated factors. Due to variations in regional health care systems and disease types, a standardised prevalence range of hospital antibiotic prescribing is lacking. However, our findings show a high prevalence of antibiotic use in hospitals worldwide. In outpatient settings, over one-third of patients received antibiotics, with 26.1% of prescriptions deemed inappropriate. In inpatient settings, nearly half of the patients were prescribed antibiotics, with 37.8% being inappropriate. Additionally, the percentage of antibiotics in the WHO-recommended ‘access’ group was below 60% threshold in both settings. These results emphasise the need for enhanced hospital antimicrobial stewardship to promote rational prescribing practices.

Our subgroup analysis showed that sub-Saharan Africa had the highest prevalence of antibiotic prescribing in outpatient settings, while South Asia had the highest prevalence in inpatient settings. In contrast, Europe and Central Asia had the lowest prevalence of antibiotic prescribing in both settings. This lower prevalence can be attributed to the region's high and upper-middle-income economies, which generally have lower rates of antibiotic prescribing. It might also reflect the significant efforts made in this region to combat AMR. The European Commission has implemented several continent-wide action plans to address AMR [26,27], introducing policies focussed on education, regulating antibiotic prescribing, and monitoring antibiotic consumption, aimed to guide member states in managing the growing AMR crisis [28]. Europe also hosts the only continent-wide monitoring network for antibiotic consumption and AMR, providing long-term data to support AMR control efforts [29,30]. Such continent-wide initiatives might effectively integrate resources and facilitate collaboration between countries.

Our findings indicate higher rates of hospital antibiotic prescribing in low- and middle-income countries (LMICs), a finding which is likely influenced by several factors. Poor sanitary conditions and limited access to safe drinking water in such contexts increase the prevalence of infectious diseases [31], leading to higher antibiotic consumption. Additionally, cultural norms often promote the preventive use of antibiotics, which is linked to limited education levels [32,33]. Inadequate health care infrastructure, including limited access to accurate laboratory diagnostics, hinders optimal antibiotic prescribing by medical personnel [34]. Furthermore, persistent challenges such as food insecurity and housing crises limit resources away from antimicrobial stewardship efforts [35–37], while inadequate antibiotic regulation contributes to inappropriate or excessive prescribing.

The WHO has developed numerous antimicrobial stewardship strategies to control AMR, and many countries (149 out of 166 countries included in the WHO’s statistics on AMR NAP implementation in 2017) have formulated national action plans [21]. However, our findings show that hospital antibiotic prescribing has not declined globally and has significantly increased in inpatient settings, which could be related to several factors. First, socioeconomic development in the 21st century has improved antibiotic availability [38], while urbanisation has increased the incidence of infectious diseases, promoting antibiotic prescribing in hospitals [39,40]. Second, the implementation of national action plans remains insufficient. Although many countries have adopted AMR NAP strategies, only 47 (28.3%) have costed and budgeted operational plans with monitoring mechanisms, and just 17 (10.2%) have allocated sufficient financial resources to support these programs [21]. Additionally, inefficiencies and bureaucratic hurdles in African countries hinder effective implementation [41]. Finally, antimicrobial stewardship measures are not yet widely adopted in the health care system. While interventions such as C-reactive protein testing and regulatory approaches like persuasion and restriction have proven effective [42–45], their application remains limited, especially in resource-constrained and poorly regulated health care settings. For the elevated prevalence of antibiotic use among hospitalised patients, we hypothesise that it may also be related to the complexity of hospital-acquired infection and the excess challenges of antimicrobial stewardship policies in inpatient settings. Additionally, the growing situation of AMR increases the risk of infectious disease transmission, the occurrence of serious illnesses, and hospital-acquired infections in hospitalised patients [46]. Events such as the COVID-19 pandemic have exacerbated these challenges [47]. More nosocomial infections may result in more antibiotic prescribing in inpatient settings. Moreover, the relative complexity of conditions among hospitalised patients makes it difficult for antimicrobial stewardship strategies to cover every medical situation, which may lead to irrational and overprescription of antibiotics.

We identified three key factors associated with hospital antibiotic prescribing, involving patients, physicians, and hospitals. These findings highlight the need to strengthen hospital antimicrobial stewardship from multiple perspectives. First, patients with lower education levels are more likely to accept antibiotics. Studies have demonstrated that patients with higher education are better informed about antibiotics, reducing pressure on physicians [48]. Improving public health literacy and raising awareness about antibiotics and AMR is therefore essential. Second, physicians play a crucial role in controlling AMR in hospitals. Their prescribing behaviour should align with strict guidelines and responsible practices, minimising negative influences from colleagues and patients. Regular feedback on prescribing patterns can promote accountability and reduce inappropriate prescribing [49], as can providing physicians with updated guidelines and integrating diagnostic tools into daily practice [50]. Finally, governments should promote appropriate antibiotic prescribing at a macro level by strengthening collaboration across different sectors and promulgating policies. Optimising antibiotic prescription practices is a complex process and systematic approaches are required.

Our study is the first systematic review to assess global hospital antibiotic prescribing practices and associated factors, offering the latest evidence-based insights to inform hospital antimicrobial stewardship. With 403 studies included, it is the most comprehensive evaluation of worldwide hospital antibiotic prescribing. However, it has some limitations. First, there was significant heterogeneity among the included studies, likely due to the differences in study contexts, samples, and outcome evaluations. Second, limitations in the original studies prevented subgroup analyses for other indicators other than the prevalence of antibiotic prescribing and its associated factors. Third, while this review includes data from the largest number of countries to date, it covers only about half of all countries worldwide. Many LMICs lack studies on hospital antibiotic prescribing, highlighting the need for more original research in these regions.

## CONCLUSIONS

This systematic review highlights the widespread prevalence of antibiotic prescribing worldwide, with no significant improvement observed over the past 20 years. This calls for a critical re-evaluation of current antimicrobial stewardship strategies. The prevalence of hospital antibiotic prescribing is high and the related research is insufficient in low-income countries specifically, underscoring a need for greater support and attention. Hospital antibiotic prescribing is influenced by a range of several factors. To improve appropriateness and standardisation, urgent actions are needed, including multi-sectoral cooperation and targeted educational initiatives.

## Additional material


Online Supplementary Document
