# Supplementary material for: Global antibiotic prescription practices in hospitals and associated factors: a systematic review and meta-analysis
Source: J Glob Health. 2025 Jan 31;15:04023. doi: 10.7189/jogh.15.04023 (PMC11781807; doi:10.7189/jogh.15.04023)
Supplement: Online Supplementary Document [file jogh-15-04023-s001.pdf]

Supplement to: Chen R, Li J, Wang C, Zhou P, Song Q, Wu J, Li Q, Li H, Gong Y, Fang Y, Zeng T, Yin X. Global antibiotic prescription practices in hospitals and associated factors: a systematic review and meta-analysis. J Glob Health. 2025;15:04023.

Supplementary Text 1. Search strategies........1

Supplementary Text 2. Research screening........5

Supplementary Text 3. Data extraction........7

Supplementary Text 4. Criteria of quality evaluation........10

Supplementary Text 5. Definitions and calculation methods of indicators........12

Supplementary Table 1. PRISMA checklist........15

Supplementary Table 2. Implementation of AMR NAP among countries........19

Supplementary Table 3. The distribution of studies by country........22

Supplementary Table 4. The distribution of studies by region and income group......25

Supplementary Table 5. Basic information of included studies........28

Supplementary Table 6. Results of quality evaluation........78

Supplementary Table 7. Inappropriate antibiotic prescribing of included studies......88

Supplementary Table 8. Antibiotic prescribing on the AWaRe classification........97

Supplementary Table 9. Other indicators of hospital antibiotic prescribing........109

Supplementary Table 10. Factors associated with hospital antibiotic prescribing....110

Supplementary Figure 1. Forest plot of outpatient antibiotic prescribing........134

Supplementary Figure 2. Outpatient antibiotic prescribing across countries........135

Supplementary Figure 3. Forest plot of inpatient antibiotic prescribing........136

Supplementary Figure 4. Year-prevalence scatter plot........137

Supplementary Text 1. Search strategies

Strategies of studies on antibiotic use in hospitals

PubMed/MEDLINE (Access date: February 28, 2023)

((("Anti-Infective Agents"[Mesh]) OR ("Anti-Bacterial Agents" [Pharmacological Action]) OR (antibiotic*[Title/Abstract]) OR (anti-bacteria*[Title/Abstract]) OR (anti-Infective*[Title/Abstract]) OR (antimicrob*[Title/Abstract]) OR (antibacterial*[Title/Abstract])) AND ((("Drug Utilization"[Mesh]) OR ("Drug Utilization Review"[Mesh]) OR ("Practice Patterns, Physicians'"[Mesh]) OR (consum*[Title]) OR (prescri*[Title]) OR (use[Title]) OR (usage[Title]) OR (utili*[Title]) OR (appropriate*[Title]) OR (pattern[Title])))) AND (("Hospitals"[Mesh]) OR (hospital*[Title/Abstract]) OR (setting*[Title/Abstract]) OR (department*[Title/Abstract]) OR (outpatient[Title/Abstract]) OR (inpatient[Title/Abstract])) Filters: English, MEDLINE, from 2000/1/1 - 2023/2/28

**Query**	**Results**
Search: **((("Anti-Infective Agents"[Mesh]) OR ("Anti-Bacterial Agents" [Pharmacological Action]) OR (antibiotic*[Title/Abstract]) OR (anti-bacteria*[Title/Abstract]) OR (anti-Infective*[Title/Abstract]) OR (antimicrob*[Title/Abstract]) OR (antibacterial*[Title/Abstract])) AND ((("Drug Utilization"[Mesh]) OR ("Drug Utilization Review"[Mesh]) OR ("Practice Patterns, Physicians'"[Mesh]) OR (consum*[Title]) OR (prescri*[Title]) OR (use[Title]) OR (usage[Title]) OR (utili*[Title]) OR (appropriate*[Title]) OR (pattern[Title])))) AND (("Hospitals"[Mesh]) OR (hospital*[Title/Abstract]) OR (setting*[Title/Abstract]) OR (department*[Title/Abstract]) OR (outpatient[Title/Abstract]) OR (inpatient[Title/Abstract]))** Filters: **English, MEDLINE, from 2000/1/1 - 2023/2/28**	12,342

Ovid/Embase (Access date: February 28, 2023)

((antibiotic* or antimicrobial* or antibacterial*).ab,ti.) and ((hospital* or setting* or department* or outpatient or inpatient).ab,ti.) and ((consump* or use* or usage or utili* or prescri* or appropriate* or pattern).ti.) and English.lg.

# ▲	Retrieve content	outcome	Retrieval method
1	((antibiotic* or antimicrobial* or antibacterial*) and (hospital* or setting* or department* or outpatient or inpatient)).ab,ti. and (consump* or use* or usage or utili* or prescri* or appropriate* or pattern).ti. and English.lg.	18275	Senior
2	limit 1 to yr="2000 - 2023"	17111	Senior

Web of Science (Access date: February 28, 2023)

(TS=(antibiotic* OR antimicrobial* OR antibacterial*)) AND (TS=(hospital* OR setting* OR department* OR outpatient OR inpatient)) AND (TI=(consump* OR use OR usage OR utili* OR prescri* OR appropriate* OR pattern)) AND (LA=English) AND (DOP=2000-01-01/2023-02-28)

☐	1	(TS=(antibiotic* OR antimicrobial* OR antibacterial*)) AND (TS=(hospital* OR setting* OR department* OR outpatient OR inpatient)) AND (TI=(consump* OR use OR usage OR utili* OR prescri* OR appropriate* OR pattern)) AND (LA=English) AND (DOP=2000-01-01/2023-02-28)	12,353	Add to query ⌄

12,353 results from Web of Science Core Collection for:

(TS=(antibiotic* OR antimicrobial* OR antibacterial*)) AND (TS=(hospital* OR setting* OR department* OR outpatient OR inpatient)) AND (TI=(c…

Strategies of studies on the associated factors

PubMed/MEDLINE (Access date: February 28, 2023)

(((("Anti-Infective Agents"[Mesh]) OR ("Anti-Bacterial Agents" [Pharmacological Action]) OR (antibiotic*[Title/Abstract]) OR (anti-bacteria*[Title/Abstract]) OR (anti-Infective*[Title/Abstract]) OR (antimicrob*[Title/Abstract]) OR (antibacterial*[Title/Abstract])) AND (("Drug Utilization"[Mesh]) OR ("Drug Utilization Review"[Mesh]) OR ("Practice Patterns, Physicians'"[Mesh]) OR (consum*[Title]) OR (prescri*[Title]) OR (use[Title]) OR (usage[Title]) OR (utili*[Title]) OR (appropriate*[Title]) OR (pattern[Title]) OR ("improve*"[Title]))) AND (("Hospitals"[Mesh]) OR (hospital*[Title/Abstract]) OR (setting*[Title/Abstract]) OR (department*[Title/Abstract]) OR (outpatient[Title/Abstract]) OR (inpatient[Title/Abstract]))) AND (("factor*"[Title/Abstract]) OR ("determinant"[Title/Abstract]) OR ("cause"[Title/Abstract]) OR ("drive"[Title/Abstract]) OR ("driving"[Title/Abstract]) OR ("predict*"[Title/Abstract]) OR ("mechanism"[Title/Abstract]) OR ("perception*"[Title/Abstract]) OR ("attitude*"[Title/Abstract]) OR ("practice*"[Title/Abstract]) OR ("reason*"[Title/Abstract])) Filters: English, MEDLINE, from 2000/1/1 - 2023/2/28

Query	Results
Search: **(((("Anti-Infective Agents"[Mesh]) OR ("Anti-Bacterial Agents" [Pharmacological Action]) OR (antibiotic*[Title/Abstract]) OR (anti-bacteria*[Title/Abstract]) OR (anti-Infective*[Title/Abstract]) OR (antimicrob*[Title/Abstract]) OR (antibacterial*[Title/Abstract])) AND (("Drug Utilization"[Mesh]) OR ("Drug Utilization Review"[Mesh]) OR ("Practice Patterns, Physicians'"[Mesh]) OR (consum*[Title]) OR (prescri*[Title]) OR (use[Title]) OR (usage[Title]) OR (utili*[Title]) OR (appropriate*[Title]) OR (pattern[Title]) OR ("improve*"[Title]))) AND (("Hospitals"[Mesh]) OR (hospital*[Title/Abstract]) OR (setting* [Title/Abstract]) OR (department*[Title/Abstract]) OR (outpatient[Title/Abstract]) OR (inpatient[Title/Abstract]))) AND (("factor*"[Title/Abstract]) OR ("determinant"[Title/Abstract]) OR ("cause"[Title/Abstract]) OR ("drive"[Title/Abstract]) OR ("driving" [Title/Abstract]) OR ("predict*"[Title/Abstract]) OR ("mechanism" [Title/Abstract]) OR ("perception*"[Title/Abstract]) OR ("attitude*" [Title/Abstract]) OR ("practice*"[Title/Abstract]) OR ("reason*" [Title/Abstract]))** Filters: **English, MEDLINE, from 2000/1/1 - 2023/2/28**	6,579

Ovid/Embase (Access date: February 28, 2023)

((antibiotic* or antimicrobial* or antibacterial*).ab,ti.) and ((hospital* or setting* or department* or outpatient or inpatient).ab,ti.) and ((consump* or use* or usage or utili* or prescri* or improve* or pattern).ti.) and ((factor*or determinant or cause or drive or driving or predict* or mechanism or perception* or attitude* or practice* or reason*).ab,ti.) and English.lg.

# ▲	Retrieve content	outcome	Retrieval method
1	((antibiotic* or antimicrobial* or antibacterial*) and (hospital* or setting* or department* or outpatient or inpatient)).ab,ti. and (consump* or use* or usage or utili* or prescri* or improve* or pattern).ti. and (factor*or determinant or cause or drive or driving or predict* or mechanism or perception* or attitude* or practice* or reason*).ab,ti. and English.lg.	7667	Senior
2	limit 1 to yr="2000 - 2023"	7348	Senior

Web of Science (Access date: February 28, 2023)

(TS=(antibiotic* OR antimicrobial* OR antibacterial*)) AND (TS=(hospital* OR setting* OR department* OR outpatient OR inpatient)) AND (TI=(consump* OR use OR usage OR utili* OR prescri* OR improve* OR pattern)) AND (TS=(factor* OR determinant OR cause OR drive OR driving OR predict* OR mechanism OR perception* OR attitude* OR practice* OR reason*)) AND (LA=English) AND (DOP=2000-01-01/2023-02-28)

2	#1 AND (LA=English) AND (DOP=2000-01-01/2023-02-28)	7,213	Add to query
1	(TS=(antibiotic* OR antimicrobial* OR antibacterial*)) AND (TS=(hospital* OR setting* OR department* OR outpatient OR inpatient)) AND (TI=(consump* OR use OR usage OR utili* OR prescri* OR improve* OR pattern)) AND (TS=(factor* OR determinant OR cause OR drive OR driving OR predict* OR mechanism OR perception* OR attitude* OR practice* OR reason*))	7,707	Add to query

7,213 results from Web of Science Core Collection for:

(TS=(antibiotic* OR antimicrobial* OR antibacterial*)) AND (TS=(hospital* OR setting* OR department* OR outpatient OR inpatient)) AND (TI=(c…

Supplementary Text 2. Research screening

Research screening was performed in three steps.

First, four authors (RC, JL, QS, and PZ) screened titles of publications to exclude studies that were explicitly unrelated to this study. Studies with primary health care settings, studies on microbiology experiments, and studies that regarded antifungal or antiviral drugs as their subject matter were excluded. Uncertainties were resolved through discussion. The consistency of reviewers in this phase was good (>95%) and the detection rate was ensured.

Second, the four authors mentioned above reviewed the abstracts of the first-step selection studies. Studies that met the following exclusion criteria were excluded.

- Reviews
- Mathematical modeling studies
- Economic analyses
- Qualitative studies
- Conference proceedings
- Abstracts
- The research focused on veterinary antibiotics
- Editorials
- Full text not available
- Reports on studies of topical antibiotic use

If it is not sure to exclude a study through abstract review, the publication would be retained for full-text evaluation. All abstracts selected by each reviewer were discussed jointly to reach a consensus for inclusion or exclusion.

Third, two authors (RC and JL) read the full text of the remaining articles and excluded studies following the above criteria. References of included studies were

screened to ensure that no relevant studies were missed. Any disagreements were discussed until a consensus was reached. Individual studies were determined for inclusion in the final analysis by consulting with one senior author (XY).

Supplementary Text 3. Data extraction

The following information from included studies was extracted.

1. Bibliographic information:

- Title of the study;
- First author;
- Year of publication.

2. Basic study information:

- Study location (country)[†];
- Year of study;
- Study type (cross-sectional study, case-control study, cohort study, the trial).

3. Settings of the study:

- The number of hospitals;
- Hospital type (public or private);
- Research population (adults, children)[‡];
- Medical setting (outpatient or inpatient).

4. Information on antibiotic prescribing in hospitals:

- The prevalence of antibiotic prescribing:
  - Total number of prescriptions or total number of patients;
  - The number of prescriptions containing at least one antibiotic or the number of patients prescribed at least one antibiotic.
- The percentage of antibiotics prescribed for combination:
  - The number of prescriptions containing at least one antibiotic or the number of patients prescribed at least one antibiotic;
  - The number of prescriptions containing two or more antibiotics or the number of patients prescribed two or more antibiotics.
- The percentage of empiric antibiotics:
  - The number of prescriptions containing at least one antibiotic or the number of patients prescribed at least one antibiotic;

    - The number of prescriptions containing empiric antibiotics or the number of patients prescribed empiric antibiotics.
- The percentage of antibiotics administered parenterally:
    - The number of prescriptions containing at least one antibiotic or the number of patients prescribed at least one antibiotic;
    - The number of prescriptions containing antibiotics administered parenterally or the number of patients prescribed antibiotics administered parenterally.
- The percentage of inappropriate antibiotic prescribing:
    - The number of prescriptions containing at least one antibiotic or the number of patients prescribed at least one antibiotic;
    - The number of prescriptions containing inappropriate antibiotics or the number of patients prescribed inappropriate antibiotics.
- The percentage of antibiotics prescribed for prophylaxis:
    - The number of prescriptions containing at least one antibiotic or the number of patients prescribed at least one antibiotic;
    - The number of prescriptions containing antibiotics for prophylaxis or the number of patients prescribed antibiotics for prophylaxis.
- The percentage of antibiotics in the Access group, Watch group, or Reserve group:
    - The number of prescriptions containing at least one antibiotic;
    - The number of prescriptions containing antibiotics in the Access group, Watch group, or Reserve group.
- Associated factors of antibiotic prescribing in hospitals (influencing factors, OR values, and their 95% CIs).

* For intervention studies in randomized and non-randomized trials, we extracted baseline data or data from the control group on antibiotic prescribing.

† According to the classification criteria developed by the World Bank, regions were divided into seven types: Europe & Central Asia, East Asia & Pacific, sub-Saharan Africa, South Asia, North

America, Latin America & the Caribbean, and Middle East & North Africa; income groups[2] of the economies where the research was conducted were classified into four categories: high income, upper middle income, lower middle income, and low income.[1] The classification of income groups was based on the standards of the year when each study was conducted (https://view.officeapps.live.com/op/view.aspx?src=https%3A%2F%2Fdatacatalogfiles.worldbank.org%2Fddh-published%2F0037712%2FDR0090754%2FOGHIST.xlsx&wdOrigin=BROWSELINK).

‡ If the patient type was not reported in a study, the proportion of children to all subjects was typically very small.[2] In this situation, the patient type of this study was recorded as "adults".

Reference

1. World Bank. World Bank Country and Lending Groups. 2023. Available: https://datahelpdesk.worldbank.org/knowledgebase/articles/906519. Accessed: 15 November 2024.
2. Saleem Z, Hassali MA, Godman B, Versporten A, Hashmi FK, Saeed H, et al. Point prevalence surveys of antimicrobial use: a systematic review and the implications. Expert Rev Anti Infect Ther. 2020;18:897-910.

Supplementary Text 4. Criteria of quality evaluation

External validity

Q1. Was the study's target population **a close representation** of the population of interest in relation to relevant variables, e.g. age, sex, occupation, health status or other? If the study's target population was a close representation of the national population, 1 point is scored; otherwise, 0 is recorded.

Q2. Was the sampling frame **a true or close representation** of the target population? If the sampling frame was a true or close representation of the target population, 1 point is scored; otherwise, 0 is recorded.

Q3. Was some form of **random selection** used to select the sample, or, was a census undertaken? If a census was undertaken, or, some form of random selection was used to select the sample (e.g. simple random sampling, stratified random sampling, cluster sampling, systematic sampling), 1 point is scored; otherwise, 0 is recorded.

Q4. Did the study **avoid inappropriate exclusion**s? If the inappropriate exclusions was avoided, 1 point is scored; otherwise, 0 is recorded.

Internal validity

Q5. Were data **collected from the clinical records** (as opposed to patients)? If the data was collected from the clinical records, 1 point is scored; otherwise, 0 is recorded.

Q6. Was an acceptable **case definition** used in the study? If an acceptable case definition was used, 1 point is scored; otherwise, 0 is recorded.

Q7. Is the study method for measuring drug prescription shown to have **reliability and validity (if necessary)**? i.e. is there an opportunity for misclassification. If the method is shown to have minimal misclassification potential, 1 point is scored; otherwise, 0 is recorded.

Q8. Was the **same mode of data collection** used for all subjects? If the same mode of data collection was used for all subjects, 1 point is scored; otherwise, 0 is recorded.

Q9. Were the **numerator(s) and denominator(s)** for the parameter of interest

appropriate? If the paper presented appropriate numerator(s) and denominator(s) for the parameter of interest, 1 point is scored; otherwise, 0 is recorded.

According to the total score, the quality of original studies was divided into three groups: high quality (8–9 points), moderate quality (6–7 points), and low quality (1–5 points).

Supplementary Text 5. Definitions and calculation methods of indicators

- **The prevalence of antibiotic prescribing**

Definition: The percentage of prescriptions containing at least one antibiotic or patients receiving at least one antibiotic.

Calculation method: $$\frac{\text{The number of prescriptions containing at least one antibiotic / The number of patients prescribed at least one antibiotic}}{\text{Total number of prescriptions / Total number of patients}} \times 100\%$$

- **The percentage of antibiotics prescribed for combination**

Definition: The percentage of prescriptions containing two or more antibiotics or patients prescribed two or more antibiotics.

Calculation method: $$\frac{\text{The number of prescriptions containing two or more antibiotics / The number of patients prescribed two or more antibiotics}}{\text{The number of prescriptions containing at least one antibiotic / The number of patients prescribed at least one antibiotic}} \times 100\%$$

- **The percentage of empiric antibiotics**

Definition: The percentage of prescriptions given for a clinical syndrome where an etiologic agent had not yet been identified.

Calculation method: $$\frac{\text{The number of prescriptions containing empiric antibiotics / The number of patients prescribed empiric antibiotics}}{\text{The number of prescriptions containing at least one antibiotic / The number of patients prescribed at least one antibiotic}} \times 100\%$$

- **The percentage of antibiotics administered parenterally**

Definition: The percentage of antibiotics administered except in the oral route, etc., intravenous route, intramuscular route, and inhaling route.

Calculation method:

$$\frac{\text{The number of prescriptions containing antibiotics administered parenterally / The number of patients prescribed antibiotics administered parenterally}}{\text{The number of prescriptions containing at least one antibiotic / The number of patients prescribed at least one antibiotic}} \times 100\%$$

- **The percentage of antibiotics prescribed for prophylaxis**

Definition: The percentage of antibiotics prescribed for both surgical and medical prophylaxis.

Calculation method:

$$\frac{\text{The number of prescriptions containing antibiotics for prophylaxis / The number of patients prescribed antibiotics for prophylaxis}}{\text{The number of prescriptions containing at least one antibiotic / The number of patients prescribed at least one antibiotic}} \times 100\%$$

- **The percentage of inappropriate antibiotic prescribing**

Definition: According to the different basis, the percentage of inappropriate antibiotic prescribing was reported in some included studies. Details are shown in Appendix 15.

Calculation method:

$$\frac{\text{The number of prescriptions containing inappropriate antibiotics / The number of patients prescribed inappropriate antibiotics}}{\text{The number of prescriptions containing at least one antibiotic / The number of patients prescribed at least one antibiotic}} \times 100\%$$

- **The percentage of antibiotics in the Access group, Watch group, or Reserve group**

According to the WHO AWaRe classification (2019 edition), antibiotics are divided into three groups. The Access group included first-line and narrow-spectrum agents, e.g., amoxicillin. The Watch group included broad-spectrum agents with higher resistance selection, e.g., azithromycin. The Reserve group includes antibiotics needed to treat multidrug-resistant microbial infections as a last resort, e.g., cefazolin.

$$\textbf{The percentage of antibiotics in the Access group} = \frac{\text{The number of prescriptions containing antibiotics in the Access group}}{\text{The number of prescriptions containing at least one antibiotic}} \times 100\%$$

$$\textbf{The percentage of antibiotics in the Watch group} = \frac{\text{The number of prescriptions containing antibiotics in the Watch group}}{\text{The number of prescriptions containing at least one antibiotic}} \times 100\%$$

$$\textbf{The percentage of antibiotics in the Reserve group} = \frac{\text{The number of prescriptions containing antibiotics in the Reserve group}}{\text{The number of prescriptions containing at least one antibiotic}} \times 100\%$$

Supplementary Table 1. PRISMA checklist

Table S1. PRISMA 2020 checklist

Section and Topic	Item #	Checklist item	Location where item is reported
**TITLE**			
Title	1	Identify the report as a systematic review.	Page1
**ABSTRACT**			
Abstract	2	See the PRISMA 2020 for Abstracts checklist.	Page 1, 2
**INTRODUCTION**			
Rationale	3	Describe the rationale for the review in the context of existing knowledge.	Page 2, 3
Objectives	4	Provide an explicit statement of the objective(s) or question(s) the review addresses.	Page 3
**METHODS**			
Eligibility criteria	5	Specify the inclusion and exclusion criteria for the review and how studies were grouped for the syntheses.	Page 4
Information sources	6	Specify all databases, registers, websites, organisations, reference lists and other sources searched or consulted to identify studies. Specify the date when each source was last searched or consulted.	Page 4
Search strategy	7	Present the full search strategies for all databases, registers and websites, including any filters and limits used.	Page4; Appendix S2
Selection process	8	Specify the methods used to decide whether a study met the inclusion criteria of the review, including how many reviewers screened each record and each report retrieved, whether they worked independently, and if applicable, details of automation tools used in the process.	Page 4; Appendix S3
Data collection process	9	Specify the methods used to collect data from reports, including how many reviewers collected data from each report, whether	Page 4, 5

Section and Topic	Item #	Checklist item	Location where item is reported
		they worked independently, any processes for obtaining or confirming data from study investigators, and if applicable, details of automation tools used in the process.	
Data items	10a	List and define all outcomes for which data were sought. Specify whether all results that were compatible with each outcome domain in each study were sought (e.g. for all measures, time points, analyses), and if not, the methods used to decide which results to collect.	Page 6, 7; Appendix S7
	10b	List and define all other variables for which data were sought (e.g. participant and intervention characteristics, funding sources). Describe any assumptions made about any missing or unclear information.	Page 6, 7
Study risk of bias assessment	11	Specify the methods used to assess risk of bias in the included studies, including details of the tool(s) used, how many reviewers assessed each study and whether they worked independently, and if applicable, details of automation tools used in the process.	Page 6, 7; Appendix S6
Effect measures	12	Specify for each outcome the effect measure(s) (e.g. risk ratio, mean difference) used in the synthesis or presentation of results.	Page 6, 7
Synthesis methods	13a	Describe the processes used to decide which studies were eligible for each synthesis (e.g. tabulating the study intervention characteristics and comparing against the planned groups for each synthesis (item #5)).	Page 6, 7
	13b	Describe any methods required to prepare the data for presentation or synthesis, such as handling of missing summary statistics, or data conversions.	Page 6, 7
	13c	Describe any methods used to tabulate or visually display results of individual studies and syntheses.	Page 7
	13d	Describe any methods used to synthesize results and provide a rationale for the choice(s). If meta-analysis was performed, describe the model(s), method(s) to identify the presence and extent of statistical heterogeneity, and software package(s) used.	Page 6, 7
	13e	Describe any methods used to explore possible causes of heterogeneity among study results (e.g. subgroup analysis, meta-regression).	Page 6, 7

Section and Topic	Item #	Checklist item	Location where item is reported
	13f	Describe any sensitivity analyses conducted to assess robustness of the synthesized results.	Page 6, 7
Reporting bias assessment	14	Describe any methods used to assess risk of bias due to missing results in a synthesis (arising from reporting biases).	NA
Certainty assessment	15	Describe any methods used to assess certainty (or confidence) in the body of evidence for an outcome.	Page 6, 7
**RESULTS**			
Study selection	16a	Describe the results of the search and selection process, from the number of records identified in the search to the number of studies included in the review, ideally using a flow diagram.	Page 7; Figure 1
	16b	Cite studies that might appear to meet the inclusion criteria, but which were excluded, and explain why they were excluded.	NA
Study characteristics	17	Cite each included study and present its characteristics.	Appendix S10
Risk of bias in studies	18	Present assessments of risk of bias for each included study.	Appendix S11
Results of individual studies	19	For all outcomes, present, for each study: (a) summary statistics for each group (where appropriate) and (b) an effect estimate and its precision (e.g. confidence/credible interval), ideally using structured tables or plots.	Table 1–3
Results of syntheses	20a	For each synthesis, briefly summarise the characteristics and risk of bias among contributing studies.	Appendix S10
	20b	Present results of all statistical syntheses conducted. If meta-analysis was done, present for each the summary estimate and its precision (e.g. confidence/credible interval) and measures of statistical heterogeneity. If comparing groups, describe the direction of the effect.	Page 8–10
	20c	Present results of all investigations of possible causes of heterogeneity among study results.	Table 1–2
	20d	Present results of all sensitivity analyses conducted to assess the robustness of the synthesized results.	Page 8–10
Reporting biases	21	Present assessments of risk of bias due to missing results (arising from reporting biases) for each synthesis assessed.	NA
Certainty of evidence	22	Present assessments of certainty (or confidence) in the body of evidence for each outcome assessed.	Appendix S11

Section and Topic	Item #	Checklist item	Location where item is reported
**DISCUSSION**			
Discussion	23a	Provide a general interpretation of the results in the context of other evidence.	Page 11
	23b	Discuss any limitations of the evidence included in the review.	Page 14
	23c	Discuss any limitations of the review processes used.	Page 14
	23d	Discuss implications of the results for practice, policy, and future research.	Page 14
**OTHER INFORMATION**			
Registration and protocol	24a	Provide registration information for the review, including register name and registration number, or state that the review was not registered.	Page 3
	24b	Indicate where the review protocol can be accessed, or state that a protocol was not prepared.	Page 3
	24c	Describe and explain any amendments to information provided at registration or in the protocol.	NA
Support	25	Describe sources of financial or non-financial support for the review, and the role of the funders or sponsors in the review.	Page 15
Competing interests	26	Declare any competing interests of review authors.	Page 15
Availability of data, code and other materials	27	Report which of the following are publicly available and where they can be found: template data collection forms; data extracted from included studies; data used for all analyses; analytic code; any other materials used in the review.	NA

Supplementary Table 2. Implementation of AMR NAP among countries

Table S2. Implementation condition of AMR NAP among countries

Country	Whether to implement	Year of implementation
Argentina	YES	2022
Australia	YES	2021
Austria	YES	2017
Bangladesh	YES	2021
Barbados	YES	2021
Belgium	YES	2022
Benin	YES	2022
Botswana	NO	
Brazil	YES	2021
Canada	NO	
China	YES	2018
Colombia	YES	2021
Congo, Rep	YES	2021
Croatia	YES	2018
Cuba	YES	2022
Egypt, Arab Rep.	YES	2021
El Salvador	YES	2022
Eritrea	YES	2022
Eswatini	YES	2022
Ethiopia	YES	2019
Fiji	YES	2021
France	YES	2021
Gambia, The	NO	
Georgia	YES	2021
Germany	YES	2021
Ghana	YES	2020
Greece	YES	2022
Guyana	NO	
India	YES	2021
Indonesia	YES	2020
Iran, Islamic Rep.	YES	2021
Iraq	YES	2021
Ireland	YES	2021
Israel	NO	
Italy	YES	2021
Japan	YES	2018
Jordan	YES	2019

Country	Whether to implement	Year of implementation
Kenya	YES	2019
Korea, Rep.	YES	2018
Kosovo	NO	
Kuwait	NO	
Kyrgyz Republic	YES	2022
Lao PDR	YES	2021
Latvia	YES	2021
Liberia	YES	2020
Lithuania	YES	2022
Malaysia	YES	2018
Mexico	YES	2021
Mongolia	YES	2021
Montenegro	YES	2021
Mozambique	YES	2021
Myanmar	YES	2021
Nepal	NO	
Netherlands	YES	2017
New Zealand	YES	2022
Nigeria	YES	2021
Norway	YES	2017
Pakistan	YES	2019
Palestine	No data available	
Paraguay	YES	2021
Peru	YES	2020
Philippines	YES	2019
Poland	NO	
Russian Federation	YES	2021
Saudi Arabia	YES	2018
Serbia	YES	2021
Sierra Leone	YES	2022
Singapore	YES	2018
Slovak Republic	YES	2021
Somalia	NO	
South Africa	YES	2021
Spain	YES	2020
Sri Lanka	YES	2019
St. Lucia	No data available	
Sudan	NO	
Sweden	YES	2017
Switzerland	YES	2019
Tanzania	YES	2018
Thailand	YES	2018

Country	Whether to implement	Year of implementation
Trinidad and Tobago	NO	
Türkiye	YES	2021
Uganda	YES	2021
United Arab Emirates	YES	2020
United Kingdom	YES	2021
United States	YES	2018
Venezuela, RB	No data available	
Vietnam	YES	2021
Yemen, Rep.	YES	2022
Zambia	YES	2021
Zimbabwe	YES	2021

Supplementary Table 3. The distribution of studies by country

Table S3. The distribution of studies by country

Country*	No. of studies
Argentina	1
Australia	6
Austria	3
Bangladesh	2
Barbados	1
Belgium	3
Benin	1
Botswana	1
Brazil	7
Bulgaria	1
Canada	8
China	35
Colombia	1
Congo, Rep	1
Croatia	4
Cuba	1
Cyprus	1
Czech Republic	1
Egypt, Arab Rep.	2
El Salvador	1
Eritrea	1
Estonia	2
Eswatini	1
Ethiopia	22
Fiji	1
Finland	2
France	6
Gambia, The	2
Georgia	1
Germany	5
Ghana	10
Greece	7
Guyana	1
Hungary	1
Iceland	1
India	23
Indonesia	4

Country*	No. of studies
Iran, Islamic Rep.	3
Iraq	1
Ireland	2
Israel	2
Italy	20
Japan	7
Jordan	6
Kenya	7
Korea, Rep.	2
Kuwait	1
Kyrgyz Republic	1
Lao PDR	1
Latvia	6
Lebanon	1
Liberia	1
Lithuania	3
Luxembourg	1
Malaysia	4
Malta	1
Mexico	2
Mongolia	1
Montenegro	1
Mozambique	2
Myanmar	1
Nepal	5
Netherlands	5
New Zealand	1
Nigeria	18
Norway	5
Pakistan	10
Paraguay	1
Peru	2
Philippines	1
Poland	2
Portugal	2
Romanian	1
Russian Federation	2
Saudi Arabia	4
Serbia	2
Sierra Leone	2
Singapore	1
Slovak Republic	1

Country*	No. of studies
Slovakia	1
Slovenia	1
Somalia	1
South Africa	8
Spain	7
Sri Lanka	3
St. Lucia	1
Sudan	1
Sweden	2
Switzerland	7
Tanzania	4
Thailand	6
Trinidad and Tobago	1
Türkiye	15
Uganda	4
United Arab Emirates	2
United Kingdom	12
United States	45
Venezuela, RB	1
Vietnam	2
Yemen, Rep.	1
Zambia	4
Zimbabwe	1

* In eight studies, the study was conducted in several countries, the information on antibiotic use and (or) the associated factors of a single country can not be extracted separately. These studies were not counted in the table above. In nine studies, the study was conducted in several countries, the information on antibiotic use and (or) the associated factors of a single country can be extracted separately. Among these studies, a study was conducted in Cuba, Mexico, El Salvador, Peru, and Paraguay; a study was conducted in Tanzania, Kenya, and Sri Lanka; a study was conducted in Brazil, Colombia, Mexico, and Bolivarian Republic of Venezuela; a study was conducted in Latvia, France, and United Kingdom; a study was conducted in Ghana, Uganda, Zambia, and Tanzania; a study was conducted in Barbados, Guyana, and St. Lucia; a study was conducted in Germany and Croatia; a study was conducted in Croatia, Estonia, Latvia, Lithuania, and Sweden; a study was conducted in 29 counties in Europe, including Austria, Belgium, Bulgaria, Croatia, Cyprus, Czech Republic, Estonia, Finland, France, Germany, Greece, Hungary, Iceland, Ireland, Italy, Latvia, Lithuania, Luxembourg, Malta, Netherlands, Norway, Poland, Portugal, Romanian, Slovakia, Slovenia, Spain, United Kingdom, and Serbia.

Supplementary Table 4. The distribution of studies by region and income group

Table S4-1. Summary of country classification (by region)

Region*	No. of reports†	No. of studies	No. of countries	No. of global countries	Percentage (%)‡	Included countries
Europe & Central Asia	184	104	38	58	65.5	Austria, Belgium, Bulgaria, Croatia, Cyprus, Czech Republic, Estonia, Finland, France, Georgia Germany, Greece, Hungary, Iceland, Ireland, Italy, Japan, Kyrgyz Republic, Latvia, Lithuania, Luxembourg, Malta, Montenegro, Netherlands, Norway, Poland, Portugal, Romanian, Russian Federation, Serbia, Slovak Republic, Slovakia, Slovenia, Spain, Sweden, Switzerland, Türkiye, United Kingdom.
Sub-Saharan Africa	100	88	20	48	41.7	Benin, Botswana, Eritrea, Eswatini, Ethiopia, Ghana, Kenya, Liberia, Mozambique, Nigeria, Sierra Leone, Somalia, South Africa, Republic of the Congo, Republic of the Gambia, Sudan, Tanzania, Uganda, Zambia, Zimbabwe.
East Asia & Pacific	116	72	15	38	39.5	Australia, China, Fiji, Indonesia, Japan, Republic of Korea, Lao PDR, Malaysia, Mongolia, Myanmar, New Zealand, Philippines, Singapore, Thailand, Vietnam.
North America	29	55	2	3	66.7	Canada, United States.
South Asia	48	43	5	8	62.5	Bangladesh, India, Nepal, Pakistan, Sri Lanka.
Middle East & North Africa	24	23	10	21	47.6	Iraq, Israel, Jordan, Kuwait, Lebanon, Saudi Arabia, United Arab Emirates, The Arab Republic of Egypt, The Islamic Republic of Iran, The Islamic Republic of Yemen.
Latin America & the Caribbean	22	12	13	42	31.0	Argentina, Barbados, Brazil, Colombia, Cuba, El Salvador, Guyana, Mexico, Paraguay, Peru, St. Lucia, The Bolivarian Republic of Venezuela, Trinidad and Tobago.

* The classification of regions was based on the latest World Bank standards (https://datahelpdesk.worldbank.org/knowledgebase/articles/906519). One study reported the data in sub-Saharan Africa and South Asia, this study was counted twice when calculating the number of studies of the two regions. Six studies reported the antibiotic use of several regions, and related data of each region can not be extracted separately.

† Only including reports on the prevalence of antibiotic use in hospitals.

‡ Percentage indicates the ratio of the number of countries and regions covered in this study to the number of all countries and regions worldwide.

Table S4-2. Summary of country classification (by income group)

Income group*	No. of reports†	No. of studies	No. of countries and regions	No. of global countries	Percentage (%)‡	Included countries and regions
H	220	173	43	81	54.1	Australia, Austria, Barbados, Belgium, Canada, Cyprus, Czech Republic, Estonia, Finland, France, Germany, Greece, Hong Kong SAR (China), Hungary, Iceland, Ireland, Israel, Italy, Japan, Kuwait, Latvia, Lithuania, Malta, Netherlands, New Zealand, Norway, Poland, Portugal, Republic of Korea, Saudi Arabia, Singapore, Slovak Republic, Slovakia, Slovenia, Spain, Sweden, Switzerland, Türkiye, Taiwan (China), Trinidad and Tobago, United Arab Emirates, United Kingdom, United States.
UM	139	86	33	54	61.1	Argentina, Botswana, Brazil, Bulgaria, China, Colombia, Croatia, Estonia, Fiji, Georgia, Germany, Guyana, Indonesia, Iraq, Jordan, Latvia, Lebanon, Lithuania, Malaysia, Mexico, Montenegro, Paraguay, Peru, Romanian, Russian Federation, Serbia, South Africa, Sri Lanka, St. Lucia, The Islamic Republic of Iran, Türkiye, Thailand, Bolivarian Republic of Venezuela.
LM	99	85	25	54	46.3	Bangladesh, Brazil, China, Eswatini, Ghana, India, Indonesia, Jordan, Kenya, Lao PDR, Mongolia, Myanmar, Nepal, Nigeria, Pakistan, Philippines, Sri Lanka, Sudan, Tanzania, Thailand, The Arab Republic of Egypt, The Islamic Republic of Iran, Vietnam, Zambia, Zimbabwe.

Income group*	No. of reports†	No. of studies	No. of countries and regions	No. of global countries	Percentage (%)‡	Included countries and regions
L	59	57	21	28	75.0	Benin, Democratic Republic of the Congo, Eritrea, Ethiopia, Ghana, India, Indonesia, Kenya, Kyrgyz Republic, Liberia, Mozambique, Nepal, Nigeria, Republic of the Congo, Sierra Leone, Somalia, Tanzania, The Islamic Republic of Yemen, Uganda, Vietnam, Zambia.

* The classification of income group was based on the World Bank standards of the year when each study was conducted (https://view.officeapps.live.com/op/'view.aspx?src=https%3A%2F%2Fdatacatalogfiles.worldbank.org%2Fddh-published%2F0037712%2FDR0090754%2FOGHIST.xlsx&wdOrigin=BROWSELINK), some countries appear twice or more times in the above table since income group of these countries changes among time. H(High income), UM(Upper middle income), LM(Lower middle income), L(Low income). Nine studies reported the data of several income groups, and related data of each group can not be extracted separately. One study reported the data in low-income, lower-middle-income, and upper-middle-income economies, this study was counted three times when calculating the number of studies of the three income groups. One study reported the data in low-income and low-middle-income economies, this study was counted twice when calculating the number of studies of the two income groups. Four studies reported the data in upper-middle-income and high-income economies, the four studies were counted twice when calculating the number of studies of the two income groups.

† Only including reports on the prevalence of antibiotic use in hospitals.

‡ Percentage indicates the ratio of the number of countries and regions covered in this study to the number of all countries and regions worldwide.

Supplementary Table 5. Basic information of included studies

Table S5-1. The main characteristics of included studies

Characteristic	No. of studies†	Percentage (%)
Total	403	100.0
Region		
East Asia & Pacific	104	26.6
Sub-Saharan Africa	88	21.2
Europe & Central Asia	72	18.1
North America	55	13.9
South Asia	43	10.4
Middle East & North Africa	23	5.6
Latin America & the Caribbean	12	2.8
Not specified*	6	1.4
Income group		
High income	173	43.8
Upper middle income	86	20.2
Lower middle income	85	20.2
Low income	57	13.7
Not specified*	9	2.1
Year of publication		
2010 and before	68	17.8
2011–2015	57	18.2

Characteristic	No. of studies†	Percentage (%)
After 2015	263	64.8
Quality assessment		
High	109	27.0
Moderate	222	55.1
Low	72	17.9

* Some studies reported the data of several regions or several income groups, and related data of each region or income group can not be extracted separately.

† One study reported the data in sub-Saharan Africa and South Asia, this study was counted twice when calculating the number of studies of the two regions. Therefore, when calculating the percentage of each region, the total number of studies was set to 425. One study reported the data in low-income, lower-middle-income, and upper-middle-income economies, this study was counted three times when calculating the number of studies of the three groups of economies. One study reported the data in low-income and low-middle-income economies, this study was counted twice when calculating the number of studies of the two groups of economies. Four studies reported the data in upper-middle-income and high-income economies, the four studies were counted twice when calculating the number of studies of the two groups of economies. Therefore, when calculating the percentage of each income group, the total number of studies was set to 431.

Table S5-2. Basic information of included studies

Study	Country	Region*	Income group*,†	Year of study	Patient type	Medical setting	Reported information‡	Research quality
Fentie AM et al., 2022 [1]	Ethiopia	Sub-Saharan Africa	L	2021	Both	Inpatient	T C E P A I F	Moderate
Levy HG et al., 2022 [2]	Cuba; Mexico; El Salvador; Peru; Paraguay	Latin America & the Caribbean	UM	2019	Adults	Inpatient	T E P I G	High
Moulin E et al., 2022 [3]	Switzerland	Europe & Central Asia	H	2021	Adults	Inpatient	T P A I G	Moderate
Magill SS et al., 2021 [4]	United States	North America	H	2011 2015	Adults	Inpatient	T P G	High
Mustafa ZU et al., 2022 [5]	Pakistan	South Asia	LM	2020	Children	Inpatient	T C P A G	High

Study	Country	Region*	Income group*,†	Year of study	Patient type	Medical setting	Reported information‡	Research quality
Muro FJ et al., 2022 [6]	Tanzania; Kenya; Sri Lanka	Sub-Saharan Africa South Asia	L LM UM	2018	Adults	Inpatient	T C	Moderate
Yi S et al., 2022 [7]	Liberia	Sub-Saharan Africa	L	2019	Adults	Outpatient	T C G	Moderate
Xavier SP et al., 2022 [8]	Mozambique	Sub-Saharan Africa	L	2019	Children	Inpatient	T C A I	Moderate
Haseeb A et al., 2021 [9]	Saudi Arabia	Middle East & North Africa	H	2019	Adults	Inpatient	T E P A I G	High
Ogunleye OO et al., 2022 [10]	Nigeria	Sub-Saharan Africa	LM	2019	Adults	Inpatient	T C A G	Moderate
Zhao H et al., 2021 [11]	China	East Asia & Pacific	UM	2014–2018	Adults	Outpatient	T I	High
Da Silva RMR et al., 2021 [12]	Brazil	Latin America & the Caribbean	UM	2018	Adults	Inpatient	T	Moderate
Boone K et al., 2021 [13]	Bangladesh	South Asia	LM	2014–2016	Children	Inpatient	T G F	Moderate
Obura B et al., 2021 [14]	Uganda	Sub-Saharan Africa	L	2019	Children	Inpatient	T	Moderate
Kruger D et al., 2021 [15]	South Africa	Sub-Saharan Africa	UM	2016	Adults	Inpatient	T	Moderate
Kurdi A et al., 2021 [16]	Iraq	Middle East & North Africa	UM	2019	Adults	Inpatient	T C E P A G	Moderate
Skosana PP et al., 2021 [17]	South Africa	Sub-Saharan Africa	UM	2018	Adults	Inpatient	T A G	High
Arif S et al., 2021 [18]	Pakistan	South Asia	LM	2018–2019	Children	Inpatient	T C E P A	Moderate
Suljagic V et al., 2021 [19]	Serbia	Europe & Central Asia	UM	2017	Adults	Inpatient	T P	Moderate
German GJ et al., 2021 [20]	Canada	North America	H	2018	Adults	Inpatient	T P	Moderate
Almansoori N et al., 2021 [21]	South Africa	Sub-Saharan Africa	UM	2018	Adults	Outpatient	T C A I	Moderate
Blackburn J et al., 2021 [22]	Canada	North America	H	2018–2019	Children	Inpatient	T C P G	High
Pauwels I et al., 2021 [23]	69 countries	NS	NS	2015–2018	Adults	Inpatient	G	Moderate
Oguz E et al., 2021 [24]	Türkiye	Europe & Central Asia	UM	2016	Children	Inpatient	T E A	Moderate
Tassew SG et al., 2021 [25]	Ethiopia	Sub-Saharan Africa	L	2019	Adults	Inpatient	T G	Moderate
Yehualaw A et al., 2021 [26]	Ethiopia	Sub-Saharan Africa	L	2018–2019	Children	Inpatient	A I G	Moderate
Atal S et al., 2021 [27]	India	South Asia	LM	2016–2018	Adults	Outpatient	T	Moderate

Study	Country	Region*	Income group*,†	Year of study	Patient type	Medical setting	Reported information‡	Research quality
Shrestha JTM et al., 2021 [28]	Nepal	South Asia	LM	2019–2020	Adults	Outpatient	T	Moderate
Wendie TF et al., 2021 [29]	Ethiopia	Sub-Saharan Africa	L	2019	Adults	Outpatient	T G	Moderate
de Guzman Betito G et al., 2021 [30]	Philippines	East Asia & Pacific	LM	2017–2019	Adults	Inpatient	T P G	Moderate
Magill SS et al., 2012 [31]	United States	North America	H	2009	Adults	Inpatient	T	Moderate
Chandelkar U et al., 2014 [32]	India	South Asia	LM	2014	Adults	Inpatient	T	Low
Momanyi L et al., 2019 [33]	Kenya	Sub-Saharan Africa	LM	2017	Adults	Inpatient	T C E I G	Moderate
Mama M et al., 2020 [34]	Ethiopia	Sub-Saharan Africa	L	2018–2019	Adults	Inpatient	C A I	Moderate
Rachina S et al., 2020 [35]	Russian Federation	Europe & Central Asia	UM	2015 2017 2018	Adults	Inpatient	T C E A I	Moderate
Barchitta M et al., 2020 [36]	Italy	Europe & Central Asia	H	2016–2018	Adults	Inpatient	T	Moderate
Afriyie DK et al., 2020 [37]	Ghana	Sub-Saharan Africa	LM	2019	Both	Inpatient	T P	Low
Liang JJ et al., 2022 [38]	Canada	North America	H	2002 2009 2017	Adults	Inpatient	T	Moderate
Soltani J et al., 2018 [39]	Iran, Islamic Rep.	Middle East & North Africa	UM	2011–2012	Children	Inpatient	T E G	Moderate
Holen O et al., 2017 [40]	Norway	Europe & Central Asia	H	2016	Adults	Inpatient	T P	Low
Ahoyo TA et al., 2014 [41]	Benin	Sub-Saharan Africa	L	2012	Adults	Inpatient	T	Moderate
Charani E et al., 2019 [42]	United Kingdom	Europe & Central Asia	H	2016–2017	Adults	Inpatient	T C A I	Moderate
Ughasoro MD et al., 2019 [43]	Nigeria	Sub-Saharan Africa	LM	2016	Children	Inpatient	T C E A	Moderate
Ofori-Adjei YA et al., 2019 [44]	Ghana	Sub-Saharan Africa	LM	2013	Adults	Outpatient	T	Low
Gürtler N et al., 2019 [45]	Switzerland	Europe & Central Asia	H	2017	Adults	Inpatient	T E P A I	High
Umeokonkwo CD et al., 2019 [46]	Nigeria	Sub-Saharan Africa	LM	2017	Adults	Inpatient	T P	Low
Hufnagel M et al., 2019 [47]	41 countries	NS	NS	2012	Children	Inpatient	T P	High
Zhang Y et al., 2019 [48]	China	East Asia & Pacific	UM	2017–2018	Adults	Inpatient	T	Moderate
Ciofi Degli Atti ML et al., 2019 [49]	Italy	Europe & Central Asia	H	2008–2016	Children	Inpatient	T P F	High
Sheng T et al., 2019 [50]	Sri Lanka	South Asia	LM	2017	Adults	Inpatient	T C I	Moderate

Study	Country	Region*	Income group*,†	Year of study	Patient type	Medical setting	Reported information‡	Research quality
Tersigni C et al., 2019 [51]	Italy	Europe & Central Asia	H	2016	Children	Inpatient	T	Low
Saleem Z et al., 2019 [52]	Pakistan	South Asia	LM	2017	Adults	Inpatient	T C E P A G	High
Thaulow CM et al., 2019 [53]	Norway	Europe & Central Asia	H	2015–2017	Children	Inpatient	T	Moderate
Huerta-Gutiérrez R et al., 2019 [54]	Brazil; Colombia; Mexico; Venezuela, RB	Latin America & the Caribbean	UM	2016	Adults	Inpatient	T P A	Moderate
Labi AK et al., 2019 [55]	Ghana	Sub-Saharan Africa	LM	2016	Adults	Inpatient	T	High
Ripabelli G et al., 2019 [56]	Italy	Europe & Central Asia	H	2016	Adults	Inpatient	T C P A	Moderate
Luthander J et al., 2019 [57]	Sweden	Europe & Central Asia	H	2003–2017	Children	Inpatient	T C P A	High
Dlamini NN et al., 2019 [58]	South Africa	Sub-Saharan Africa	UM	2017	Adults	Inpatient	T	Moderate
Li H et al., 2019 [59]	China	East Asia & Pacific	UM	2012–2016	Adults	Both	T C A	High
Zingg W et al., 2019 [60]	Switzerland	Europe & Central Asia	H	2017	Adults	Inpatient	T	High
Singh SK et al., 2019 [61]	India	South Asia	LM	2017	Adults	Inpatient	T P	Moderate
Dyer AP et al., 2019 [62]	United States	North America	H	2016	Adults	Inpatient	T	High
Peralta N et al., 2019 [63]	Argentina	Latin America & the Caribbean	UM	2018	Adults	Inpatient	T P	High
Al Matar M et al., 2019 [64]	Saudi Arabia	Middle East & North Africa	H	2016	Adults	Inpatient	T P A G	Moderate
Komagamine J et al., 2019 [65]	Japan	East Asia & Pacific	H	2018	Adults	Inpatient	T P G	Moderate
Yimenu DK et al., 2019 [66]	Ethiopia	Sub-Saharan Africa	L	2019	Adults	Outpatient	T A G	Moderate
EmyInumaru F et al., 2019 [67]	Brazil	Latin America & the Caribbean	UM	2015	Children	Inpatient	T A	Moderate
Komagamine J et al., 2019 [68]	Japan	East Asia & Pacific	H	2018	Adults	Inpatient	T I G	High
Lakoh S et al., 2020 [69]	Sierra Leone	Sub-Saharan Africa	L	2017–2018	Adults	Inpatient	T C P	High
Porto APM et al., 2020 [70]	Brazil	Latin America & the Caribbean	UM	2017	Both	Inpatient	T C P A	High
Resurreccion-Delgado C et al., 2020	Peru	Latin America & the Caribbean	UM	2018	Adults	Inpatient	T C E	Moderate

Study	Country	Region*	Income group*,†	Year of study	Patient type	Medical setting	Reported information‡	Research quality
[71]								
Fowotade A et al., 2020 [72]	Nigeria	Sub-Saharan Africa	LM	2017	Adults	Inpatient	T E P A	Moderate
Tribble AC et al., 2020 [73]	United States	North America	H	2016–2017	Children	Inpatient	T	Moderate
Hafeez M et al., 2020 [74]	Pakistan	South Asia	LM	2018	Children	Inpatient	T P	Moderate
Komagamine J et al., 2020 [75]	Japan	East Asia & Pacific	H	2020	Adults	Outpatient	T P G	Moderate
Masich AM et al., 2020 [76]	Zambia	Sub-Saharan Africa	LM	2018	Adults	Inpatient	T C I G	Moderate
Frenette C et al., 2020 [77]	Canada	North America	H	2017	Both	Inpatient	T E P	Moderate
Roberts AA et al., 2020 [78]	Nigeria	Sub-Saharan Africa	LM	2018	Children	Inpatient	T E P A	Low
Tham DWJ et al., 2020 [79]	Malaysia	East Asia & Pacific	UM	2015	Children	Outpatient	T F	Moderate
Maina M et al., 2020 [80]	Kenya	Sub-Saharan Africa	LM	2018	Both	Inpatient	T I	Moderate
Griffith HG et al., 2020 [81]	United States	North America	H	2017–2018	Children	Inpatient	T C A	Moderate
Chautrakarn S et al., 2020 [82]	Thailand	East Asia & Pacific	UM	2016	Children	Inpatient	T C I	Moderate
Khan Z et al., 2020 [83]	Pakistan	South Asia	LM	2017–2018	Adults	Outpatient	T G	Moderate
Yilma Z et al., 2020 [84]	Ethiopia	Sub-Saharan Africa	L	2019–2020	Adults	Outpatient	T G	Moderate
Yilma Z et al., 2020 [85]	Ethiopia	Sub-Saharan Africa	L	2016	Adults	Outpatient	T	Low
Nepal A et al., 2020 [86]	Nepal	South Asia	L	2017	Adults	Both	T F	Moderate
Frost HM et al., 2020 [87]	United States	North America	H	2017–2018	Adults	Both	T	Moderate
Miao R et al., 2020 [88]	China	East Asia & Pacific	UM	2018	Children	Inpatient	T A	Moderate
Bimba HV et al., 2020 [89]	India	South Asia	LM	2017	Adults	Both	T A	Moderate
Seni J et al., 2020 [90]	Tanzania	Sub-Saharan Africa	LM	2019	Adults	Inpatient	T C P G F	High
Karabay O et al., 2020 [91]	Türkiye	Europe & Central Asia	UM	2018	Adults	Inpatient	T C	Moderate
Abubakar U et al., 2020 [92]	Nigeria	Sub-Saharan Africa	LM	2019	Both	Inpatient	T C P A G	Moderate
Horumpende PG et al., 2020 [93]	Tanzania	Sub-Saharan Africa	L	2016	Adults	Inpatient	T P G	Moderate

Study	Country	Region*	Income group*,†	Year of study	Patient type	Medical setting	Reported information‡	Research quality
Vandael E et al., 2020 [94]	Belgium	Europe & Central Asia	H	2017	Adults	Inpatient	T A	High
Mohammed ZA et al., 2020 [95]	India	South Asia	LM	2018	Adults	Inpatient	T C E I G	Moderate
Jaggi P et al., 2020 [96]	United States	North America	H	2016–2017	Children	Inpatient	T	Moderate
Thamlikitkul V et al., 2020 [97]	Thailand	East Asia & Pacific	UM	2018	Adults	Inpatient	T C P	High
Al-Tawfiq JA et al., 2020 [98]	Saudi Arabia	Middle East & North Africa	H	2017–2019	Adults	Inpatient	T C E	Low
Antonioli P et al., 2020 [99]	Italy	Europe & Central Asia	H	2016 2018	Adults	Inpatient	T	High
Loftus MJ et al., 2020 [100]	Fiji	East Asia & Pacific	UM	2019	Adults	Inpatient	T C P A G	Moderate
Gardiner SJ et al., 2020 [101]	New Zealand	East Asia & Pacific	H	2017–2018	Adults	Inpatient	T C P A I G	Moderate
Xu JJ et al., 2020 [102]	China	East Asia & Pacific	UM	2012–2018	Children	Inpatient	T C P G	High
Olaru ID et al., 2020 [103]	Zimbabwe	Sub-Saharan Africa	LM	2018	Children	Inpatient	T C A G	Moderate
Wang CN et al., 2020 [104]	China	East Asia & Pacific	UM	2018	Children	Outpatient	T C A G	Moderate
Zhang M et al., 2020 [105]	China	East Asia & Pacific	UM	2015–2018	Children	Inpatient	T C G	Moderate
Cusini A et al., 2010 [106]	Switzerland	Europe & Central Asia	H	2008	Adults	Inpatient	T C E P I	High
Black E et al., 2018 [107]	Canada	North America	H	2015	Adults	Inpatient	T	High
Oduyebo OO et al., 2017 [108]	Nigeria	Sub-Saharan Africa	LM	2015	Adults	Inpatient	T P A	Low
Monteiro LGS et al., 2017 [109]	Mozambique	Sub-Saharan Africa	L	2015	Children	Inpatient	T A F	Low
Nair V et al., 2015 [110]	India	South Asia	LM	2014	Adults	Inpatient	T C P G	Moderate
Singh S et al., 2014 [111]	India	South Asia	LM	2012	Children	Inpatient	T E P A	Low
Denny KJ et al., 2019 [112]	Australia	East Asia & Pacific	H	2016	Adults	Outpatient	T I F	Moderate
Sikkens JJ et al., 2018 [113]	Netherlands	Europe & Central Asia	H	2011–2012	Adults	Inpatient	T I F	Moderate
Croche Santander B et al., 2018 [114]	Spain	Europe & Central Asia	H	2013	Children	Outpatient	T I	Moderate
Labi AK et al., 2018 [115]	Ghana	Sub-Saharan Africa	LM	2016	Adults	Inpatient	T C P A G	High
Olivier C et al., 2018 [116]	South Africa	Sub-Saharan Africa	UM	2016	Children	Inpatient	T	Moderate

Study	Country	Region*	Income group*,†	Year of study	Patient type	Medical setting	Reported information‡	Research quality
Labi AK et al., 2018 [117]	Ghana	Sub-Saharan Africa	LM	2016	Children	Inpatient	T C P A	High
Zhang JS et al., 2018 [118]	China	East Asia & Pacific	UM	2016–2017	Children	Inpatient	T C	Moderate
Shrestha B et al., 2018 [119]	Nepal	South Asia	L	2017	Adults	Inpatient	T G	Moderate
Mengistu G et al., 2020 [120]	Ethiopia	Sub-Saharan Africa	L	2018	Adults	Outpatient	T	Moderate
Plachouras D et al., 2018 [121]	29 countries	Europe & Central Asia	UM H	2016–2017	Adults	Inpatient	T C P A	High
Al-Taani GM et al., 2018 [122]	United Kingdom	Europe & Central Asia	H	2009 2011 2015	Adults	Inpatient	T P A	Moderate
Gutema G et al., 2018 [123]	Ethiopia	Sub-Saharan Africa	L	2015	Adults	Inpatient	T C	High
Sticchi C et al., 2018 [124]	Italy	Europe & Central Asia	H	2016	Adults	Inpatient	T C P	High
Elhajji FD et al., 2018 [125]	United Kingdom	Europe & Central Asia	H	2015	Adults	Inpatient	T P	High
Koopmans LR et al., 2018 [126]	South Africa	Sub-Saharan Africa	UM	2015	Children	Inpatient	T P A	Moderate
Okoth C et al., 2018 [127]	Kenya	Sub-Saharan Africa	LM	2017	Adults	Inpatient	T P A	Moderate
Metsini A et al., 2018 [128]	Switzerland	Europe & Central Asia	H	2016	Adults	Inpatient	T C P	Moderate
Morioka H et al., 2018 [129]	Japan	East Asia & Pacific	H	2016	Adults	Inpatient	T P	Moderate
Amaha ND et al., 2018 [130]	Eritrea	Sub-Saharan Africa	L	2017	Adults	Inpatient	T C	Moderate
Sviestina I et al., 2018 [131]	Latvia	Europe & Central Asia	UM	2011–2013	Children	Inpatient	T C A G	High
Yan K et al., 2018 [132]	China	East Asia & Pacific	UM	2013–2015	Adults	Inpatient	T	Moderate
Umar LW et al., 2018 [133]	Nigeria	Sub-Saharan Africa	LM	2013–2014	Children	Inpatient	T C	Moderate
Chaw PS et al., 2018 [134]	Gambia, The	Sub-Saharan Africa	L	2015	Children	Inpatient	T G	High
Hussain S et al., 2018 [135]	India	South Asia	LM	2015	Adults	Outpatient	T A	Moderate
Cairns S et al., 2018 [136]	United Kingdom	Europe & Central Asia	H	2016	Both	Inpatient	T	High
Wang S et al., 2018 [137]	China	East Asia & Pacific	UM	2016	Adults	Outpatient	T C I	Low
Wang J et al., 2017 [138]	China	East Asia & Pacific	UM	2015	Adults	Inpatient	T C P	High
Chen Y et al., 2017 [139]	China	East Asia & Pacific	UM	2014–2015	Adults	Inpatient	T C P	High

Study	Country	Region*	Income group*,†	Year of study	Patient type	Medical setting	Reported information‡	Research quality
Ioannou P et al., 2022 [140]	Greece	Europe & Central Asia	H	2022	Adults	Inpatient	T P A	Moderate
Zeng L et al., 2017 [141]	China	East Asia & Pacific	UM	2013	Children	Outpatient	T C	Moderate
Kebede HK et al., 2017 [142]	Ethiopia	Sub-Saharan Africa	L	2012–2014	Children	Inpatient	T C P A G	High
Goycochea-Valdivia WA et al., 2017 [143]	Spain	Europe & Central Asia	H	2013	Children	Inpatient	T E P I G	Moderate
Sisay M et al., 2017 [144]	Ethiopia	Sub-Saharan Africa	L	2014	Adults	Outpatient	T G	Moderate
Cai Y et al., 2017 [145]	Singapore	East Asia & Pacific	H	2015–2016	Adults	Inpatient	T C P A G	High
Guclu E et al., 2017 [146]	Türkiye	Europe & Central Asia	UM	2014	Adults	Inpatient	T	Moderate
Liao XP et al., 2017 [147]	China	East Asia & Pacific	UM	2008–2010	Children	Inpatient	T	Low
Fleming-Dutra KE et al., 2016 [148]	United States	North America	H	2011–2012	Both	Outpatient	T	Moderate
Lee ML et al., 2016 [149]	Taiwan, China	East Asia & Pacific	H	2000–2009	Children	Outpatient	T F	High
Versporten A et al., 2016 [150]	41 countries	NS	NS	2012	Children	Inpatient	T C P A	High
Morioka H et al., 2016 [151]	Japan	East Asia & Pacific	H	2014	Adults	Inpatient	T P	Moderate
Fahimzad A et al., 2016 [152]	Iran, Islamic Rep.	Middle East & North Africa	UM	2014	Children	Inpatient	T E P A	Moderate
Gharbi M et al., 2016 [153]	United Kingdom	Europe & Central Asia	H	2012	Children	Inpatient	T P A	High
Ren N et al., 2016 [154]	China	East Asia & Pacific	UM	2012	Adults	Inpatient	T C P	Low
De Luca M et al., 2016 [155]	Italy	Europe & Central Asia	H	2012	Children	Inpatient	T C P	Moderate
Stefkovicova M et al., 2016 [156]	Slovak Republic	Europe & Central Asia	H	2012	Adults	Inpatient	T P A G	High
Kiguba R et al., 2016 [157]	Uganda	Sub-Saharan Africa	L	2014	Adults	Inpatient	T A G	High
Bilal AI et al., 2016 [158]	Ethiopia	Sub-Saharan Africa	L	2014	Adults	Outpatient	T G	Moderate
Knezevic B et al., 2016 [159]	Australia	East Asia & Pacific	H	2013–2014	Adults	Inpatient	T I	Moderate
Segagni Lusignani L et al., 2016 [160]	Austria	Europe & Central Asia	H	2012	Adults	Inpatient	T P A	High
Antonioli P et al., 2016 [161]	Italy	Europe & Central Asia	H	2011–2013	Adults	Inpatient	T A	Moderate

Study	Country	Region*	Income group*,†	Year of study	Patient type	Medical setting	Reported information‡	Research quality
Vercheval C et al., 2016 [162]	Belgium	Europe & Central Asia	H	2012–2013	Adults	Inpatient	T C A	High
Marani A et al., 2016 [163]	Italy	Europe & Central Asia	H	2007–2015	Adults	Inpatient	T P	Moderate
Zhang YW et al., 2016 [164]	China	East Asia & Pacific	UM	2012–2014	Adults	Inpatient	T P	Moderate
Atif M et al., 2017 [165]	Pakistan	South Asia	LM	2016	Adults	Inpatient	T C G	High
Atif M et al., 2016 [166]	Pakistan	South Asia	LM	2014–2015	Adults	Outpatient	T	Moderate
Akhtar M et al., 2012 [167]	India	South Asia	LM	2008	Children	Outpatient	T	Low
Henry CN et al., 2013 [168]	Nigeria	Sub-Saharan Africa	LM	2010–2011	Adults	Outpatient	T	Moderate
Angamo MT et al., 2011 [169]	Ethiopia	Sub-Saharan Africa	L	2009	Adults	Outpatient	T	High
Woldu MA et al., 2013 [170]	Ethiopia	Sub-Saharan Africa	L	2012	Children	Inpatient	T C	High
Al-Niemat SI et al., 2014 [171]	Jordan	Middle East & North Africa	UM	2012–2013	Children	Outpatient	T C	High
Mohajer KA et al., 2011 [172]	Saudi Arabia	Middle East & North Africa	H	2007	Children	Outpatient	T	Moderate
Wang H et al., 2013 [173]	China	East Asia & Pacific	UM	2011–2012	Adults	Outpatient	T	Moderate
Osowicki J et al., 2015 [174]	Australia	East Asia & Pacific	H	2012	Children	Inpatient	T C E P I	Moderate
Amadeo B et al., 2010 [175]	21 countries	Europe & Central Asia	NS	2008	Children	Inpatient	T C P A G	High
Magill SS et al., 2014 [176]	United States	North America	H	2011	Adults	Inpatient	T C P	High
Aldeyab MA et al., 2012 [177]	United Kingdom	Europe & Central Asia	H	2009	Adults	Inpatient	T P A	High
Talaat M et al., 2014 [178]	Egypt, Arab Rep.	Middle East & North Africa	LM	2011	Adults	Inpatient	T C P	Moderate
Akhloufi H et al., 2015 [179]	Netherlands	Europe & Central Asia	H	2013	Adults	Inpatient	T C P I	High
Sinatra I et al., 2013 [180]	Italy	Europe & Central Asia	H	2011	Adults	Inpatient	T C P	Moderate
Gizework A et al., 2015 [181]	Ethiopia	Sub-Saharan Africa	L	2012–2013	Children	Inpatient	T C	Low
Gandra S et al., 2017 [182]	India	South Asia	LM	2016–2017	Children	Inpatient	T C P	Moderate
Nsofor CA et al., 2016 [183]	Nigeria	Sub-Saharan Africa	LM	2013	Adults	Inpatient	T C G F	Moderate
Mulwa Charles N et al., 2015 [184]	Kenya	Sub-Saharan Africa	L	2013	Adults	Outpatient	T	Moderate

Study	Country	Region*	Income group*,†	Year of study	Patient type	Medical setting	Reported information‡	Research quality
Bashrahil KA et al., 2010 [185]	Yemen, Rep.	Middle East & North Africa	L	2003	Adults	Inpatient	T	Low
Mudenda W et al., 2016 [186]	Zambia	Sub-Saharan Africa	LM	2015	Adults	Outpatient	T	Moderate
Joda AE et al., 2013 [187]	Nigeria	Sub-Saharan Africa	LM	2000–2001	Adults	Outpatient	T	Moderate
Choudhury DK et al., 2013 [188]	India	South Asia	LM	2011	Children	Inpatient	T C	Low
Jose MW et al., 2016 [189]	Gambia, The	Sub-Saharan Africa	L	2014	Adults	Inpatient	T C G	Moderate
Assen A et al., 2014 [190]	Ethiopia	Sub-Saharan Africa	L	2013	Adults	Outpatient	T G	Low
Li C et al., 2013 [191]	China	East Asia & Pacific	UM	2001 2003 2005 2008 2010	Adults	Inpatient	T C	Moderate
Versporten A et al., 2013 [192]	23 countries	NS	NS	2011	Children	Inpatient	T	Moderate
Sviestina I et al., 2015 [193]	Latvia; France; United Kingdom	Europe & Central Asia	H	2012	Children	Inpatient	T A	High
Sviestina I et al., 2013 [194]	United Kingdom	Europe & Central Asia	H	2011	Children	Inpatient	T A	Moderate
Bergicho M et al., 2012 [195]	Ethiopia	Sub-Saharan Africa	L	2007–2009	Children	Inpatient	T	Moderate
Xie DS et al., 2015 [196]	China	East Asia & Pacific	UM	2007	Adults	Inpatient	T C P A	Moderate
Fadare J et al., 2015 [197]	Nigeria	Sub-Saharan Africa	LM	2013	Children	Outpatient	T	Moderate
Xie DS et al., 2015 [198]	China	East Asia & Pacific	UM	2008	Adults	Inpatient	T C P A	Moderate
Alfandari S et al., 2015 [199]	France	Europe & Central Asia	H	2010	Adults	Inpatient	T	Moderate
van Spreuwel PC et al., 2015 [200]	United States	North America	H	2013	Adults	Inpatient	T E P A	Moderate
Cotta MO et al., 2014 [201]	Australia	East Asia & Pacific	H	2012	Adults	Inpatient	T I	Moderate
Sviestina I et al., 2014 [202]	Latvia	Europe & Central Asia	H	2012	Children	Inpatient	T C	Moderate
Osowicki J et al., 2014 [203]	Australia	East Asia & Pacific	H	2012	Children	Inpatient	T P I G	Moderate
Bozkurt F et al., 2014 [204]	Türkiye	Europe & Central Asia	UM	2011	Adults	Inpatient	T P	Moderate
Muyu G et al., 2013 [205]	Kenya	Sub-Saharan Africa	L	2012	Adults	Outpatient	T	Low

Study	Country	Region*	Income group*,†	Year of study	Patient type	Medical setting	Reported information‡	Research quality
Desalegn AA et al., 2013 [206]	Ethiopia	Sub-Saharan Africa	L	2007–2009	Adults	Outpatient	T G	High
Sozen H et al., 2013 [207]	Türkiye	Europe & Central Asia	UM	2011	Adults	Inpatient	T E	Moderate
Behnke M et al., 2013 [208]	Germany	Europe & Central Asia	H	2011	Adults	Inpatient	T P	High
Mabiala Babela JR et al., 2013 [209]	Congo, Rep	Sub-Saharan Africa	L	2009	Children	Inpatient	T	Moderate
Borras Novell C et al., 2013 [210]	Spain	Europe & Central Asia	H	2009–2010	Children	Inpatient	T C A G	Moderate
Zarb P et al., 2012 [211]	23 countries	Europe & Central Asia	NS	2010	Adults	Inpatient	T P A	Moderate
Thu TA et al., 2012 [212]	Vietnam	East Asia & Pacific	L	2008	Both	Inpatient	T C E F	High
Ingram PR et al., 2012 [213]	Australia	East Asia & Pacific	H	2010	Adults	Inpatient	T C E A I	Low
Robert J et al., 2012 [214]	France	Europe & Central Asia	H	2009	Adults	Inpatient	T C P	High
Levy ER et al., 2012 [215]	United States	North America	H	2007–2010	Children	Inpatient	T C	Moderate
Sharma M et al., 2012 [216]	India	South Asia	LM	2008	Adults	Inpatient	T C	High
Baktygul K et al., 2011 [217]	Kyrgyz Republic	Europe & Central Asia	L	2007	Adults	Inpatient	T	High
Carneiro M et al., 2011 [218]	Brazil	Latin America & the Caribbean	UM	2009	Adults	Inpatient	T	Moderate
Pathak A et al., 2011 [219]	India	South Asia	LM	2007–2009	Adults	Outpatient	T	High
Xie DS et al., 2010 [220]	China	East Asia & Pacific	UM	2007–2008	Both	Inpatient	T C P	Moderate
Ceyhan M et al., 2010 [221]	Türkiye	Europe & Central Asia	UM	2007	Children	Inpatient	T C I	Moderate
Willemsen I et al., 2010 [222]	Netherlands	Europe & Central Asia	H	2008–2009	Adults	Inpatient	T C A	Moderate
Kolyva S et al., 2017 [223]	Greece	Europe & Central Asia	H	2012–2013	Children	Outpatient	T I	Moderate
Larru B et al., 2016 [224]	United States	North America	H	2012	Children	Inpatient	T	Low
Sharma S et al., 2016 [225]	United States	North America	H	2012	Children	Outpatient	T C	High
Aly NY et al., 2012 [226]	Kuwait	Middle East & North Africa	H	2008	Adults	Inpatient	T I	High
Gailiene G et al., 2012 [227]	Lithuania	Europe & Central Asia	UM	2010	Adults	Inpatient	T C E P	Moderate
Askarian M et al., 2012 [228]	Iran, Islamic Rep.	Middle East & North Africa	LM	2008–2009	Adults	Inpatient	T	Moderate

Study	Country	Region*	Income group*,†	Year of study	Patient type	Medical setting	Reported information‡	Research quality
Dryden M et al., 2012 [229]	United Kingdom	Europe & Central Asia	H	2010	Adults	Inpatient	T A G	Moderate
Gomez-Gomez J et al., 2015 [230]	Austria	Europe & Central Asia	H	2012	Adults	Inpatient	T C E P I	Moderate
Gugliotta C et al., 2020 [231]	Italy	Europe & Central Asia	H	2018	Adults	Inpatient	T P A	Low
Ababneh MA et al., 2021 [232]	Jordan	Middle East & North Africa	UM	2018	Adults	Inpatient	T	High
Mijovic G et al., 2020 [233]	Montenegro	Europe & Central Asia	UM	2015	Adults	Inpatient	T P	Low
Wushouer H et al., 2020 [234]	China	East Asia & Pacific	UM	2015–2016	Adults	Outpatient	T C	Moderate
Chui CSL et al., 2020 [235]	Hong Kong SAR, China	East Asia & Pacific	H	2000–2015	Adults	Inpatient	T	Moderate
Murni IK et al., 2015 [236]	Indonesia	East Asia & Pacific	LM	2011	Children	Inpatient	T I	High
Korinteli IG et al., 2019 [237]	Georgia	Europe & Central Asia	UM	2015 2017 2018	Children	Inpatient	T E	Low
Gibbons CL et al., 2019 [238]	United Kingdom	Europe & Central Asia	H	2016	Children	Inpatient	T C P A	Moderate
Okoro RN et al., 2019 [239]	Nigeria	Sub-Saharan Africa	LM	2013	Adults	Outpatient	T C G F	Moderate
Cole CP et al., 2018 [240]	Sierra Leone	Sub-Saharan Africa	L	2012–2013	Adults	Outpatient	T	Low
Arnoldo L et al., 2019 [241]	Italy	Europe & Central Asia	H	2011 2013 2015 2017	Adults	Inpatient	T P G	Moderate
Mao W et al., 2019 [242]	China	East Asia & Pacific	UM	2011 2013	Adults	Outpatient	T C	Moderate
Nguyen S et al., 2018 [243]	France	Europe & Central Asia	H	2014	Adults	Inpatient	T	Moderate
Palms DL et al., 2018 [244]	United States	North America	H	2014	Adults	Outpatient	T	Moderate
Naughton C et al., 2011 [245]	Ireland	Europe & Central Asia	H	2007	Adults	Inpatient	T	Moderate
Al-Azayzih A et al., 2017 [246]	Jordan	Middle East & North Africa	UM	2014	Adults	Outpatient	T	Moderate
Gerber JS et al., 2010 [247]	United States	North America	H	2008	Children	Inpatient	T	Moderate
Ider BE et al., 2010 [248]	Mongolia	East Asia & Pacific	LM	2008	Adults	Inpatient	T	Moderate
Arcavi L et al., 2010 [249]	Israel	Middle East & North Africa	H	2007–2008	Children	Inpatient	T	Moderate

Study	Country	Region*	Income group*,†	Year of study	Patient type	Medical setting	Reported information‡	Research quality
Ahmed AM et al., 2010 [250]	Sudan	Sub-Saharan Africa	LM	2007	Children	Outpatient	T	Moderate
Mohlala G et al., 2010 [251]	South Africa	Sub-Saharan Africa	UM	2005	Adults	Inpatient	T	Low
Evirgen O et al., 2011 [252]	Türkiye	Europe & Central Asia	UM	2009	Adults	Inpatient	T	Low
Etienne P et al., 2011 [253]	France	Europe & Central Asia	H	2007–2008	Adults	Inpatient	T C E I	Low
Gwebu P et al., 2022 [254]	Eswatini	Sub-Saharan Africa	LM	2021	Adults	Inpatient	T P A G	Moderate
Gulmez SE et al., 2022 [255]	Türkiye	Europe & Central Asia	UM	2019	Adults	Inpatient	T	Moderate
Guzeloglu E et al., 2022 [256]	Türkiye	Europe & Central Asia	UM	2018	Children	Inpatient	T	Moderate
Park SY et al., 2022 [257]	Korea, Rep.	East Asia & Pacific	H	2018	Adults	Both	T C P A	Moderate
Kitt E et al., 2022 [258]	Botswana	Sub-Saharan Africa	UM	2018	Children	Inpatient	T I F	Moderate
Yang Q et al., 2022 [259]	China	East Asia & Pacific	UM	2016–2019	Adults	Outpatient	T C I	Moderate
Talaat M et al., 2022 [260]	7 countries	NS	NS	2019	Adults	Inpatient	T P G	Moderate
Sheikh S et al., 2022 [261]	India	South Asia	LM	2021	Adults	Inpatient	T C	Moderate
Zhang JS et al., 2022 [262]	China	East Asia & Pacific	UM	2016	Children	Inpatient	T G	High
Shaikh Q et al., 2022 [263]	Pakistan	South Asia	LM	2021	Adults	Inpatient	T C E P A G	Moderate
Oghuvwu S et al., 2023 [264]	Nigeria	Sub-Saharan Africa	LM	2020	Adults	Outpatient	T	Low
Ranasinghe P et al., 2022 [265]	Sri Lanka	South Asia	LM	2019	Adults	Outpatient	T C	Moderate
Akkawi ME et al., 2022 [266]	Malaysia	East Asia & Pacific	UM	2020	Children	Inpatient	T C I G F	Moderate
Nunez-Nunez M et al., 2022 [267]	Spain	Europe & Central Asia	H	2012–2019	Adults	Inpatient	C E I F	Moderate
Omulo S et al., 2022 [268]	Kenya	Sub-Saharan Africa	LM	2018	Adults	Inpatient	T C	Moderate
Gutiérrez-Urbón J et al., 2022 [269]	Spain	Europe & Central Asia	H	2021	Adults	Inpatient	T C	Moderate
Joshi R et al., 2022 [270]	India	South Asia	LM	2020	Adults	Outpatient	T	Moderate
Nguyen HQ et al., 2022 [271]	Vietnam	East Asia & Pacific	LM	2016–2020	Children	Inpatient	T G	High
Sharif M et al., 2022 [272]	Pakistan	South Asia	LM	2020	Adults	Inpatient	T E P A G	Low

Study	Country	Region*	Income group*,†	Year of study	Patient type	Medical setting	Reported information‡	Research quality
Versporten A et al., 2018 [273]	53 countries	NS	NS	2015	Adults	Inpatient	T P	Moderate
Saleem Z et al., 2022 [274]	Pakistan	South Asia	LM	2020	Adults	Inpatient	T E P A	High
Limato R et al., 2021 [275]	Indonesia	East Asia & Pacific	UM	2019	Adults	Inpatient	T C P A G	High
Ishibashi N et al., 2022 [276]	Japan	East Asia & Pacific	H	2019	Adults	Inpatient	T G	Moderate
Jamaluddin NAH et al., 2021 [277]	Malaysia	East Asia & Pacific	UM	2019	Adults	Inpatient	T C E P A	Moderate
Ashour RH et al., 2022 [278]	Egypt, Arab Rep.	Middle East & North Africa	LM	2019	Adults	Inpatient	T C P A G	Moderate
Panditrao AM et al., 2021 [279]	India	South Asia	LM	2019	Adults	Inpatient	T C E P A G	Moderate
Rashid MM et al., 2022 [280]	Bangladesh	South Asia	LM	2021	Adults	Inpatient	T C P G	Moderate
Ankrah D et al., 2021 [281]	Ghana	Sub-Saharan Africa	LM	2019	Adults	Inpatient	T C E P A	High
D'Arcy N et al., 2021 [282]	Ghana; Uganda; Zambia; Tanzania	Sub-Saharan Africa	LM L	2019	Adults	Inpatient	T P G	Moderate
Kiggundu R et al., 2022 [283]	Uganda	Sub-Saharan Africa	L	2021	Adults	Inpatient	T C P A G	Moderate
Wang CN et al., 2021 [284]	China	East Asia & Pacific	UM	2019	Children	Inpatient	T C G	High
Rocke T et al., 2022 [285]	Barbados; Guyana; St. Lucia	Latin America & the Caribbean	H UM	2013 2018	Adults	Inpatient	T G	Low
Oo WT et al., 2022 [286]	Myanmar	East Asia & Pacific	LM	2019	Adults	Inpatient	T P A	Moderate
Spernovasilis N et al., 2022 [287]	Greece	Europe & Central Asia	H	2019	Adults	Inpatient	T E P	Moderate
Okoye BI et al., 2022 [288]	Nigeria	Sub-Saharan Africa	LM	2022	Children	Outpatient	T	Moderate
El-Dahiyat F et al., 2022 [289]	United Arab Emirates	Middle East & North Africa	H	2018	Children	Outpatient	T A G	Low
Mittal N et al., 2023 [290]	India	South Asia	LM	2021	Adults	Inpatient	T C	Moderate
Sidamo T et al., 2022 [291]	Somalia	Sub-Saharan Africa	L	2020	Adults	Inpatient	T C	Moderate
Tadesse TY et al., 2022 [292]	Ethiopia	Sub-Saharan Africa	L	2021	Adults	Inpatient	T P A G	Moderate

Study	Country	Region*	Income group*,†	Year of study	Patient type	Medical setting	Reported information‡	Research quality
Sodhi B et al., 2022 [293]	India	South Asia	LM	2022	Children	Outpatient	T A	Moderate
Amponsah OKO et al., 2022 [294]	Ghana	Sub-Saharan Africa	LM	2021	Adults	Outpatient	T G	Moderate
Skosana PP et al., 2022 [295]	South Africa	Sub-Saharan Africa	UM	2018	Children	Inpatient	T P A	High
Yimer YS et al., 2022 [296]	Ethiopia	Sub-Saharan Africa	L	2021	Adults	Outpatient	T	Moderate
Deiana G et al., 2022 [297]	Italy	Europe & Central Asia	H	2021	Adults	Inpatient	T C P	Moderate
Meenakshi R et al., 2022 [298]	India	South Asia	LM	2019	Adults	Outpatient	T	Moderate
Abu Farha R et al., 2022 [299]	Jordan	Middle East & North Africa	UM	2021	Adults	Outpatient	T G	Moderate
Alnajjar MS et al., 2022 [300]	United Arab Emirates	Middle East & North Africa	H	2020	Adults	Inpatient	T P A	Moderate
Anugulruengkitt S et al., 2022 [301]	Thailand	East Asia & Pacific	UM	2020	Adults	Inpatient	T C E P G	Moderate
Parathoduvil AA et al., 2022 [302]	India	South Asia	LM	2017	Adults	Inpatient	T C	Moderate
Kalungia AC et al., 2022 [303]	Zambia	Sub-Saharan Africa	L	2021	Adults	Inpatient	T	Moderate
Catho G et al., 2022 [304]	Switzerland	Europe & Central Asia	H	2019	Adults	Inpatient	T	Moderate
Chansamouth V et al., 2022 [305]	Lao PDR	East Asia & Pacific	LM	2017–2020	Adults	Inpatient	T C P A G	High
Usluer G et al., 2005 [306]	Türkiye	Europe & Central Asia	H	2002	Adults	Inpatient	T C E	Low
Ufer M et al., 2005 [307]	Germany; Croatia	Europe & Central Asia	H UM	2003	Children	Inpatient	T	Low
McGregor JC et al., 2006 [308]	United States	North America	H	2004	Adults	Inpatient	T	Moderate
Avci IY et al., 2006 [309]	Türkiye	Europe & Central Asia	H	2004	Adults	Outpatient	T C A	Low
Likic R et al., 2007 [310]	Croatia	Europe & Central Asia	UM	2001	Adults	Inpatient	T	Low
Seaton RA et al., 2007 [311]	United Kingdom	Europe & Central Asia	H	2003	Adults	Inpatient	T	Low
Lee MK et al., 2007 [312]	Hong Kong SAR, China	East Asia & Pacific	H	2005	Adults	Inpatient	T C	Low
Vlahovic-Palcevski V et al., 2007 [313]	Croatia; Estonia;	Europe & Central Asia	UM H	2003	Adults	Inpatient	T C A	Low

Study	Country	Region*	Income group*,†	Year of study	Patient type	Medical setting	Reported information‡	Research quality
	Latvia; Lithuania; Sweden							
Al-Niemat SI et al., 2008 [314]	Jordan	Middle East & North Africa	LM	2007	Adults	Outpatient	T	Low
Akande TM et al., 2007 [315]	Nigeria	Sub-Saharan Africa	L	2004–2005	Adults	Outpatient	T C	Low
Oshikoya KA et al., 2007 [316]	Nigeria	Sub-Saharan Africa	L	2006	Children	Outpatient	T	Low
Danchaivijitr S et al., 2007 [317]	Thailand	East Asia & Pacific	LM	2006	Adults	Inpatient	T	Low
Hajdu A et al., 2007 [318]	Russian Federation	Europe & Central Asia	UM	2006	Children	Inpatient	T C	Low
Tunger O et al., 2008 [319]	Türkiye	Europe & Central Asia	H	2005	Adults	Inpatient	T	Low
Dimina E et al., 2009 [320]	Latvia	Europe & Central Asia	UM	2003–2007	Adults	Inpatient	T C	High
de With K et al., 2009 [321]	Germany	Europe & Central Asia	H	2006	Adults	Inpatient	T	Moderate
Ciofi Degli Atti ML et al., 2008 [322]	Italy	Europe & Central Asia	H	2007	Children	Inpatient	T C E P	Low
Hariharan S et al., 2009 [323]	Trinidad and Tobago	Latin America & the Caribbean	H	2006	Adults	Inpatient	T C	Moderate
Dimri S et al., 2009 [324]	India	South Asia	L	2006	Children	Outpatient	T	Low
Newman MJ et al., 2009 [325]	Ghana	Sub-Saharan Africa	L	2000	Adults	Inpatient	T C	Low
Ghimire S et al., 2009 [326]	Nepal	South Asia	L	2008	Adults	Outpatient	T	Low
Calligaris L et al., 2009 [327]	Italy	Europe & Central Asia	H	2008	Adults	Inpatient	T P	Moderate
Ansari F et al., 2009 [328]	20 countries	Europe & Central Asia	NS	2006	Adults	Inpatient	T P	Moderate
Rivero M et al., 2000 [329]	Spain	Europe & Central Asia	H	1996	Adults	Inpatient	T E P	Low
Erbay A et al., 2003 [330]	Türkiye	Europe & Central Asia	H	2001	Adults	Inpatient	T C A G	Low
Potocki M et al., 2003 [331]	Switzerland	Europe & Central Asia	H	2001	Children	Inpatient	T C E P G	Low
Mora Y et al., 2002 [332]	United States	North America	H	1997–1998	Children	Inpatient	T	Low
Raveh D et al., 2001 [333]	Israel	Middle East & North Africa	H	1998	Adults	Inpatient	T A G	Low

Study	Country	Region*	Income group*,†	Year of study	Patient type	Medical setting	Reported information‡	Research quality
Tunger O et al., 2000 [334]	Türkiye	Europe & Central Asia	H	1998	Adults	Inpatient	T C E P G	Low
Berild D et al., 2002 [335]	Norway	Europe & Central Asia	H	1996–1999	Adults	Inpatient	T C	Moderate
McDonald LC et al., 2001 [336]	Taiwan, China	East Asia & Pacific	H	1999	Adults	Inpatient	T A	Low
Berild D et al., 2002 [337]	Norway	Europe & Central Asia	H	1996–1998	Children	Inpatient	T	Low
Apisarnthanarak A et al., 2006 [338]	Thailand	East Asia & Pacific	LM	2004	Adults	Inpatient	T E	Low
Karande S et al., 2005 [339]	India	South Asia	L	2001	Children	Outpatient	T	Low
Fonseca LG et al., 2004 [340]	Brazil	Latin America & the Caribbean	UM	2001–2002	Adults	Inpatient	T C P	Low
Starakis I et al., 2002 [341]	Greece	Europe & Central Asia	H	1998–1999	Adults	Inpatient	T C E	Low
Chukwuani CM et al., 2002 [342]	Nigeria	Sub-Saharan Africa	L	1999	Adults	Both	T	Low
Gikas A et al., 2002 [343]	Greece	Europe & Central Asia	H	1999	Adults	Inpatient	T C	Low
Kanerva M et al., 2007 [344]	Finland	Europe & Central Asia	H	2005	Adults	Inpatient	T C	High
Willemsen I et al., 2007 [345]	Netherlands	Europe & Central Asia	H	2004	Adults	Inpatient	T C G	Low
Shankar PR et al., 2006 [346]	Nepal	South Asia	L	2003–2004	Children	Inpatient	T C	Low
Desai NM et al., 2020 [347]	United States	North America	H	2010–2015	Children	Outpatient	F	Moderate
Anteneh DA et al., 2021 [348]	Ethiopia	Sub-Saharan Africa	L	2020	Adults	Inpatient	F	High
Cotter JM et al., 2022 [349]	United States	North America	H	2013–2017	Children	Inpatient	F	Moderate
Dilworth TJ et al., 2022 [350]	United States	North America	H	2019	Adults	Outpatient	F	Low
Kroening-Roche JC et al., 2012 [351]	United States	North America	H	2008	Adults	Outpatient	F	Low
Linder JA et al., 2003 [352]	United States	North America	H	2001–2022	Adults	Outpatient	F	Moderate
Kornblith AE et al., 2018 [353]	United States	North America	H	2002–2013	Children	Outpatient	F	High
Kozyrskyj AL et al., 2004 [354]	Canada; United States	North America	H	1996–2000	Children	Outpatient	F	Moderate
Curt AM et al., 2020 [355]	United States	North America	H	2015–2019	Children	Outpatient	F	Low

Study	Country	Region*	Income group*,†	Year of study	Patient type	Medical setting	Reported information‡	Research quality
Kumar R et al., 2008 [356]	India	South Asia	L	2005	Adults	Outpatient	F	Moderate
Cantrell R et al., 2002 [357]	United States	North America	H	1996	Adults	Outpatient	F	High
Arnold SR et al., 2005 [358]	Canada	North America	H	2002	Children	Outpatient	F	Moderate
Cadieux G et al., 2011 [359]	Canada	North America	H	1993–2007	Adults	Outpatient	F	High
Barnett ML et al., 2014 [360]	United States	North America	H	1996–2010	Adults	Outpatient	F	High
Bergmark RW et al., 2016 [361]	United States	North America	H	2005–2010	Adults	Outpatient	F	Moderate
Depew RE et al., 2020 [362]	United States	North America	H	2012	Adults	Outpatient	F	High
Coco AS et al., 2009 [363]	United States	North America	H	1998–2004	Children	Outpatient	F	High
Copp HL et al., 2011 [364]	United States	North America	H	1998–2007	Children	Outpatient	F	High
Ababneh MA et al., 2017 [365]	Jordan	Middle East & North Africa	H	2014–2015	Children	Outpatient	F	Moderate
Chang LY et al., 2017 [366]	Taiwan, China	East Asia & Pacific	H	2000–2009	Children	Outpatient	F	High
Ardoino I et al., 2019 [367]	Italy	Europe & Central Asia	H	2010–2017	Adults	Inpatient	F	High
Covino M et al., 2022 [368]	Italy	Europe & Central Asia	H	2015–2020	Children	Inpatient	F	Moderate
Kourlaba G et al., 2016 [369]	Greece	Europe & Central Asia	H	2010–2013	Adults	Outpatient	F	High
Lin YC et al., 2010 [370]	Taiwan, China	East Asia & Pacific	H	2005–2006	Adults	Outpatient	F	High
Kronman MP et al., 2011 [371]	United States	North America	H	1994–2007	Children	Outpatient	F	High
Forster CS et al., 2022 [372]	United States	North America	H	2016–2017	Children	Outpatient	F	High
Goodman KE et al., 2023 [373]	United States	North America	H	2019	Adults	Inpatient	F	High
Hadi U et al., 2008 [374]	Indonesia	East Asia & Pacific	LM	2006	Adults	Inpatient	F	High
Hersh AL et al., 2011 [375]	United States	North America	H	2006–2008	Children	Outpatient	F	High
Kawanami GH et al., 2011 [376]	Brazil	Latin America & the Caribbean	LM	2005	Adults	Inpatient	F	Moderate
Jewell MJ et al., 2021 [377]	United States	North America	H	2015–2018	Children	Inpatient	F	High
Hashimoto H et al., 2019 [378]	Japan	Europe & Central Asia	H	2012–2013	Adults	Outpatient	F	High

Study	Country	Region*	Income group*,†	Year of study	Patient type	Medical setting	Reported information‡	Research quality
Hadi U et al., 2008 [379]	Indonesia	East Asia & Pacific	L	2001–2002	Adults	Inpatient	F	Moderate
Stone S et al., 2000 [380]	United States	North America	H	1996	Adults	Outpatient	F	High
Vanderweil SG et al., 2008 [381]	United States	North America	H	1993–2004	Adults	Outpatient	F	High
Shapiro DJ et al., 2014 [382]	United States	North America	H	2007–2009	Adults	Outpatient	F	High
Sencan I et al., 2022 [383]	Türkiye	Europe & Central Asia	H	2020	Adults	Inpatient	F	Moderate
Shin SM et al., 2015 [384]	Korea, Rep.	East Asia & Pacific	H	2009–2011	Children	Outpatient	F	High
Zhang Z et al., 2017 [385]	China	East Asia & Pacific	UM	2014	Children	Outpatient	F	High
Teixeira Rodrigues A et al., 2016 [386]	Portugal	Europe & Central Asia	H	2010–2012	Adults	Outpatient	F	Moderate
Velasco E et al., 2011 [387]	Germany	Europe & Central Asia	H	2007	Adults	Outpatient	F	Moderate
Havers FP et al., 2018 [388]	United States	North America	H	2013–2015	Adults	Outpatient	F	Moderate
Salzo A et al., 2021 [389]	Italy	Europe & Central Asia	H	2016	Adults	Inpatient	F	Moderate
Opoku MM et al., 2020 [390]	Ghana	Sub-Saharan Africa	LM	2015	Adults	Outpatient	F	Moderate
Morley VJ et al., 2020 [391]	United States	North America	H	2016	Adults	Outpatient	F	Low
McKay R et al., 2019 [392]	Canada	North America	H	2005–2011	Children	Outpatient	F	High
Manne M et al., 2018 [393]	United States	North America	H	2011–2012	Adults	Outpatient	F	High
Paul IM et al., 2011 [394]	United States	North America	H	1998–2007	Children	Outpatient	F	High
Moro ML et al., 2009 [395]	Italy	Europe & Central Asia	H	2003	Children	Outpatient	F	Moderate
Osatakul S et al., 2007 [396]	Thailand	East Asia & Pacific	LM	2004	Children	Outpatient	F	High
Rutschmann OT et al., 2004 [397]	United States	North America	H	1997–1999	Adults	Outpatient	F	High
Nadeem Ahmed M et al., 2010 [398]	United States	North America	H	2006	Children	Outpatient	F	High
Aspinall SL et al., 2009 [399]	United States	North America	H	2003–2004	Adults	Outpatient	F	Moderate
Ahmad A et al., 2021 [400]	Malaysia	East Asia & Pacific	UM	2018–2019	Adults	Outpatient	F	Low
Sawaya RD et al., 2020 [401]	Lebanon	Middle East & North Africa	UM	2009–2012	Children	Outpatient	F	Moderate

Study	Country	Region*	Income group*,†	Year of study	Patient type	Medical setting	Reported information‡	Research quality
Steinberg MB et al., 2016 [402]	United States	North America	H	2006–2010	Adults	Outpatient	F	High
Zhao H et al., 2020 [403]	China	East Asia & Pacific	UM	2014–2018	Adults	Outpatient	F	High

* NS(Not specified);

† H(High income), UM(Upper middle income), LM(Lower middle income), L(Low income);

‡ T(information on the prevalence of antibiotic use), C(information on the percentage of antibiotics prescribed for combination use), E(information on the percentage of empiric antibiotics), P(information on antibiotics prescribed for prophylaxis), A(information on the percentage of antibiotics administered parenterally), I(information on the percentage of inappropriate antibiotic use), G(information on the prevalence of antibiotic use in the AWaRe groups), F(information on the associated factors).

References for included studies

1. Fentie AM, Degefaw Y, Asfaw G, Shewarega W, Woldearegay M, Abebe E, et al. Multicentre point-prevalence survey of antibiotic use and healthcare-associated infections in Ethiopian hospitals. Bmj Open. 2022;12(2):e54541.

2. Levy HG, Rojas-Cortes R, Molina LH, Dreser MA, Alfonso OI, Rizo-Amezquita JN, et al. Point prevalence survey of antibiotic use in hospitals in Latin American countries. J Antimicrob Chemoth. 2022;77(3):807-15.

3. Moulin E, Boillat-Blanco N, Zanetti G, Plüss-Suard C, De Vallière S, Senn L. Point prevalence study of antibiotic appropriateness and possibility of early discharge from hospital among patients treated with antibiotics in a Swiss university hospital. Antimicrobial Resistance & Infection Control. 2022;11(1):1-8.

4. Magill SS, O'Leary E, Ray SM, Kainer MA, Evans C, Bamberg WM, et al. Antimicrobial use in US hospitals: comparison of results from emerging infections program prevalence surveys, 2015 and 2011. Clin Infect Dis. 2021;72(10):1784-92.

5. Mustafa ZU, Salman M, Yasir M, Godman B, Majeed HA, Kanwal M, et al. Antibiotic consumption among hospitalized neonates and children in Punjab province, Pakistan. Expert Rev Anti-Infe. 2022;20(6):931-9.

6. Muro FJ, Lyamuya FS, Kwobah C, Bollinger J, Bodinayake CK, Nagahawatte A, et al. Opportunities for improving antimicrobial stewardship: findings from a prospective, multi-center study in three low- or middle-income countries. Front Public Health. 2022;10:848802.

7. Yi S, Ramachandran A, Epps L, Mayah A, Burkholder TW, Jaung MS, et al. Emergency department antimicrobial use in a low-resource setting: results from a

retrospective observational study at a referral hospital in Liberia. Bmj Open. 2022;12(4):e56709.
8. Xavier SP, Victor A, Cumaquela G, Vasco MD, Rodrigues O. Inappropriate use of antibiotics and its predictors in pediatric patients admitted at the Central Hospital of Nampula, Mozambique. Antimicrob Resist in. 2022;11(1):79.
9. Haseeb A, Faidah HS, Algethamy M, Alghamdi S, Alhazmi GA, Alshomrani AO, et al. Antimicrobial usage and resistance in Makkah region hospitals: a regional point prevalence survey of public hospitals. Int J Env Res Pub He. 2021;19(1):254.
10. Ogunleye OO, Oyawole MR, Odunuga PT, Kalejaye F, Yinka-Ogunleye AF, Olalekan A, et al. A multicentre point prevalence study of antibiotics utilization in hospitalized patients in an urban secondary and a tertiary healthcare facilities in Nigeria: findings and implications. Expert Rev Anti-Infe. 2022;20(2):297-306.
11. Zhao H, Wei L, Li H, Zhang M, Cao B, Bian J, et al. Appropriateness of antibiotic prescriptions in ambulatory care in China: a nationwide descriptive database study. Lancet Infect Dis. 2021;21(6):847-57.
12. Da SR, de Mendonca S, Leao IN, Dos SQ, Batista AM, Melo MS, et al. Use of monitoring indicators in hospital management of antimicrobials. Bmc Infect Dis. 2021;21(1):827.
13. Boone K, Morris SK, Doshi S, Black J, Mohsin M, Ahmed T, et al. Antimicrobial prescribing during infant hospital admissions in a birth cohort in Dhaka, Bangladesh. J Trop Pediatrics. 2021;67(3):a93.
14. Obura B, Alele PE, Obua C. Off-label antibiotic use among paediatric in-patients: a mixed-method prospective study at a tertiary hospital in southwestern Uganda. Int J Clin Pharm-Net. 2021;43(3):637-44.
15. Kruger D, Dlamini NN, Meyer JC, Godman B, Kurdi A, Lennon M, et al. Development of a web-based application to improve data collection of antimicrobial utilization in the public health care system in South Africa. Hosp Pract (1995). 2021;49(3):184-93.
16. Kurdi A, Hasan AJ, Baker KI, Seaton RA, Ramzi ZS, Sneddon J, et al. A multicentre point prevalence survey of hospital antibiotic prescribing and quality indices in the Kurdistan regional government of Northern Iraq: the need for urgent action. Expert Rev Anti-Infe. 2021;19(6):805-14.
17. Skosana PP, Schellack N, Godman B, Kurdi A, Bennie M, Kruger D, et al. A point prevalence survey of antimicrobial utilisation patterns and quality indices amongst hospitals in South Africa; findings and implications. Expert Rev Anti-Infe. 2021;19(10):1353-66.
18. Arif S, Sadeeqa S, Saleem Z. Patterns of antimicrobial use in hospitalized children: a repeated point prevalence survey from Pakistan. J Pediat Inf Dis Soc. 2021;10(10):970-4.
19. Suljagic V, Bajcetic M, Mioljevic V, Dragovac G, Mijovic B, Janicijevic I, et al. A nationwide assessment of the burden of healthcare-associated infections and antimicrobial use among surgical patients: results from Serbian point prevalence survey, 2017. Antimicrob Resist in. 2021;10(1):47.
20. German GJ, Frenette C, Caissy JA, Grant J, Lefebvre MA, Mertz D, et al. The 2018 Global Point Prevalence Survey of antimicrobial consumption and resistance in 47

Canadian hospitals: a cross-sectional survey. CMAJ Open. 2021;9(4):E1242-51.
21. Almansoori N, Parag N. Antibiotic prescribing patterns in emergency department at regional hospital in South Africa. Afr Health Sci. 2021;21(4):1651-61.
22. Blackburn J, Barrowman N, Bowes J, Tsampalieros A, Le Saux N. Establishing benchmarks for antimicrobial use in Canadian children's hospitals: results from 2 national point prevalence surveys. Pediatr Infect Dis J. 2021;40(10):899-905.
23. Pauwels I, Versporten A, Drapier N, Vlieghe E, Goossens H. Hospital antibiotic prescribing patterns in adult patients according to the WHO Access, Watch and Reserve classification (AWaRe): results from a worldwide point prevalence survey in 69 countries. J Antimicrob Chemoth. 2021;76(6):1614-24.
24. Oguz E, Bebitoglu BT, Nuhoglu C, Cag Y, Hodzic A, Temel F, et al. Evaluation of antibiotic use among hospitalised patients in a paediatric department of a training hospital in Turkey. Int J Clin Pract. 2021;75(3):e13782.
25. Tassew SG, Abraha HN, Gidey K, Gebre AK. Assessment of drug use pattern using WHO core drug use indicators in selected general hospitals: a cross-sectional study in Tigray region, Ethiopia. Bmj Open. 2021;11(10):e45805.
26. Yehualaw A, Taferre C, Bantie AT, Demsie DG. Appropriateness and pattern of antibiotic prescription in pediatric patients at Adigart General Hospital, Tigray, Ethiopia. Biomed Res Int. 2021;2021:6640892.
27. Atal S, Jhaj R, Mathur A, Rai N, Misra S, Sadasivam B. Outpatient prescribing trends, rational use of medicine and impact of prescription audit with feedback at a tertiary care centre in India. Int J Health Plan M. 2021;36(3):738-53.
28. Shrestha J, Tiwari S, Kushwaha DK, Bhattarai P, Shrestha R. Drug prescription in the department of medicine of a tertiary care hospital according to the World Health Organization/International Network for Rational Use of Drugs Core Indicators: a descriptive cross-sectional survey. J Nepal Med Assoc. 2021;59(240):745-8.
29. Wendie TF, Ahmed A, Mohammed SA. Drug use pattern using WHO core drug use indicators in public health centers of Dessie, North-East Ethiopia. Bmc Med Inform Decis. 2021;21(1):197.
30. de Guzman BG, Pauwels I, Versporten A, Goossens H, De Los RM, Gler MT. Implementation of a multidisciplinary antimicrobial stewardship programme in a Philippine tertiary care hospital: an evaluation by repeated point prevalence surveys. J Glob Antimicrob Re. 2021;26:157-65.
31. Magill SS, Hellinger W, Cohen J, Kay R, Bailey C, Boland B, et al. Prevalence of healthcare-associated infections in acute care hospitals in Jacksonville, Florida. Infect Cont Hosp Ep. 2012;33(3):283-91.
32. Chandelkar U, Rataboli P. A study of drug prescribing pattern using WHO prescribing indicators in the state of Goa, India. International Journal of Basic & Clinical Pharmacology. 2014;3(6):1057.
33. Momanyi L, Opanga S, Nyamu D, Oluka M, Kurdi A, Godman B. Antibiotic prescribing patterns at a leading referral hospital in Kenya: a point prevalence survey. J Res Pharm Pract. 2019;8(3):149-54.

34. Mama M, Mamo A, Usman H, Hussen B, Hussen A, Morka G. Inappropriate antibiotic use among inpatients attending Madda Walabu University Goba Referral Hospital, southeast Ethiopia: implication for future use. Infect Drug Resist. 2020;13:1403-9.

35. Rachina S, Belkova Y, Kozlov R, Versporten A, Pauwels I, Goossens H, et al. Longitudinal point prevalence survey of antimicrobial consumption in Russian hospitals: results of the Global-PPS project. Antibiotics-Basel. 2020;9(8):446.

36. Barchitta M, Maugeri A, La Rosa MC, La Mastra C, Murolo G, Agodi A. Three-year trends of healthcare-associated infections and antibiotic use in acute care hospitals: findings from 2016-2018 point prevalence surveys in Sicily, Italy. Antibiotics-Basel. 2020;10(1):1.

37. Afriyie DK, Sefah IA, Sneddon J, Malcolm W, McKinney R, Cooper L, et al. Antimicrobial point prevalence surveys in two Ghanaian hospitals: opportunities for antimicrobial stewardship. Jac-Antimicrob Resis. 2020;2(1):1-9.

38. Liang JJ, Rudnick W, Mitchell R, Brooks J, Bush K, Conly J, et al. Antimicrobial use in Canadian acute-care hospitals: findings from three national point-prevalence surveys between 2002 and 2017. Infect Cont Hosp Ep. 2022;43(11):1558-64.

39. Soltani J, Pouladfar G, Versporten A, Sharland M, Soleimani N. Point prevalence survey of antimicrobial prescription and infection in pediatric and neonatal wards of two Iranian teaching hospitals. 2019;41(1):25-32.

40. Holen O, Alberg T, Blix HS, Smith I, Neteland MI, Eriksen HM. Broad-spectrum antibiotics in Norwegian hospitals. Tidsskr Norske Laege. 2017;137(5):362-6.

41. Ahoyo TA, Bankole HS, Adeoti FM, Gbohoun AA, Assavedo S, Amoussou-Guenou M, et al. Prevalence of nosocomial infections and anti-infective therapy in Benin: results of the first nationwide survey in 2012. Antimicrob Resist in. 2014;3(17):1-6.

42. Charani E, de Barra E, Rawson TM, Gill D, Gilchrist M, Naylor NR, et al. Antibiotic prescribing in general medical and surgical specialties: a prospective cohort study. Antimicrob Resist in. 2019;8(151):1-10.

43. Ughasoro MD, Nwakoby IC, Onwujekwe OE, Odike AI. Drug pooling: a cost-saving strategy to enhance antibiotics availability for pediatric in-patient in Nigeria. Niger J Clin Pract. 2019;22(2):232-7.

44. Ofori-Adjei YA, Fiakpornoo M. The influence of physicians' specialty on prescribing patterns at a general medicine out-patients clinic. Ghana Med J. 2019;53(3):204-9.

45. Gürtler N, Erba A, Giehl C, Tschudin-Sutter S, Bassetti S, Osthoff M. Appropriateness of antimicrobial prescribing in a Swiss tertiary care hospital: a repeated point prevalence survey. Swiss Med Wkly. 2019;149:w20135.

46. Umeokonkwo CD, Madubueze UC, Onah CK, Okedo-Alex IN, Adeke AS, Versporten A, et al. Point prevalence survey of antimicrobial prescription in a tertiary hospital in South East Nigeria: a call for improved antibiotic stewardship. J Glob Antimicrob Re. 2019;17:291-5.

47. Hufnagel M, Versporten A, Bielicki J, Drapier N, Sharland M, Goossens H. High rates of prescribing antimicrobials for prophylaxis in children and neonates: results from the antibiotic resistance and prescribing in European children point prevalence survey. J Pediat Inf Dis Soc. 2019;8(2):143-51.

48. Zhang Y, Zhong ZF, Chen SX, Zhou DR, Li ZK, Meng Y, et al. Prevalence of healthcare-associated infections and antimicrobial use in China: results from the 2018 point prevalence survey in 189 hospitals in Guangdong Province. Int J Infect Dis. 2019;89:179-84.

49. Ciofi DAM, D'Amore C, Ceradini J, Paolini V, Ciliento G, Chessa G, et al. Prevalence of antibiotic use in a tertiary care hospital in Italy, 2008-2016. Ital J Pediatr. 2019;45(1):63.

50. Sheng T, Wijayaratne GB, Dabrera TM, Drew RJ, Nagahawatte A, Bodinayake CK, et al. Point-prevalence study of antimicrobial use in public hospitals in southern Sri Lanka identifies opportunities for improving prescribing practices. Infect Cont Hosp Ep. 2019;40(2):224-7.

51. Tersigni C, Montagnani C, D'Argenio P, Duse M, Esposito S, Hsia Y, et al. Antibiotic prescriptions in Italian hospitalised children after serial point prevalence surveys (or pointless prevalence surveys): has anything actually changed over the years? Ital J Pediatr. 2019;45(1):127.

52. Saleem Z, Hassali MA, Versporten A, Godman B, Hashmi FK, Goossens H, et al. A multicenter point prevalence survey of antibiotic use in Punjab, Pakistan: findings and implications. Expert Rev Anti-Infe. 2019;17(4):285-93.

53. Thaulow CM, Berild D, Eriksen BH, Myklebust TA, Blix HS. Potential for more rational use of antibiotics in hospitalized children in a country with low resistance: data from eight point prevalence surveys. Pediatr Infect Dis J. 2019;38(4):384-9.

54. Huerta-Gutierrez R, Braga L, Camacho-Ortiz A, Diaz-Ponce H, Garcia-Mollinedo L, Guzman-Blanco M, et al. One-day point prevalence of healthcare-associated infections and antimicrobial use in four countries in Latin America. Int J Infect Dis. 2019;86:157-66.

55. Labi AK, Obeng-Nkrumah N, Owusu E, Bjerrum S, Bediako-Bowan A, Sunkwa-Mills G, et al. Multi-centre point-prevalence survey of hospital-acquired infections in Ghana. J Hosp Infect. 2019;101(1):60-8.

56. Ripabelli G, Salzo A, Mariano A, Sammarco ML, Tamburro M. Healthcare-associated infections point prevalence survey and antimicrobials use in acute care hospitals (PPS 2016-2017) and long-term care facilities (HALT-3): a comprehensive report of the first experience in Molise Region, Central Italy, and targeted intervention strategies. J Infect Public Heal. 2019;12(4):509-15.

57. Luthander J, Bennet R, Nilsson A, Eriksson M. Antimicrobial use in a Swedish pediatric hospital: results from eight point-prevalence surveys over a 15-year period (2003-2017). Pediatr Infect Dis J. 2019;38(9):929-33.

58. Dlamini NN, Meyer JC, Kruger D, Kurdi A, Godman B, Schellack N. Feasibility of using point prevalence surveys to assess antimicrobial utilisation in public hospitals in South Africa: a pilot study and implications. Hosp Pract (1995). 2019;47(2):88-95.

59. Li H, Yan S, Li D, Gong Y, Lu Z, Yin X. Trends and patterns of outpatient and inpatient antibiotic use in China's hospitals: data from the Center for Antibacterial Surveillance, 2012-16. J Antimicrob Chemoth. 2019;74(6):1731-40.

60. Zingg W, Metsini A, Gardiol C, Balmelli C, Behnke M, Troillet N, et al. Antimicrobial use in acute care hospitals: national point prevalence survey on

healthcare-associated infections and antimicrobial use, Switzerland, 2017. Eurosurveillance. 2019;24(33):1900015.

61. Singh SK, Sengupta S, Antony R, Bhattacharya S, Mukhopadhyay C, Ramasubramanian V, et al. Variations in antibiotic use across India: multi-centre study through Global Point Prevalence Survey. J Hosp Infect. 2019;103(3):280-3.

62. Dyer AP, Dodds AE, Anderson DJ, Sarubbi C, Wrenn R, Hicks LA, et al. Total duration of antimicrobial therapy resulting from inpatient hospitalization. Infect Cont Hosp Ep. 2019;40(8):847-54.

63. Peralta N, Camou BI, Leszczuk K, Arcidiacono D, Lerena RG, Herrera P, et al. Prevalence of hospital antibiotic use in Argentina, 2018. Infect Cont Hosp Ep. 2019;40(11):1301-4.

64. Al MM, Enani M, Binsaleh G, Roushdy H, Alokaili D, Al BA, et al. Point prevalence survey of antibiotic use in 26 Saudi hospitals in 2016. J Infect Public Heal. 2019;12(1):77-82.

65. Komagamine J, Yabuki T, Hiraiwa T. A trend in prevalence of antimicrobial use and appropriateness of antimicrobial therapy in an acute care hospital from 2018 to 2019: repeated prevalence surveys in Japan. Bmc Res Notes. 2019;12(1):811.

66. Yimenu DK, Emam A, Elemineh E, Atalay W. Assessment of antibiotic prescribing patterns at outpatient pharmacy using World Health Organization prescribing indicators. J Prim Care Communit. 2019;10:922934690.

67. EmyInumaru F, Silva A, Soares AS, Schuelter-Trevisol F. Profile and appropriate use of antibiotics among children in a general hospital in southern Brazil. Rev Paul Pediatr. 2019;37(1):27-33.

68. Komagamine J, Yabuki T, Kobayashi M, Okabe T. Prevalence of antimicrobial use and active healthcare-associated infections in acute care hospitals: a multicentre prevalence survey in Japan. Bmj Open. 2019;9(6):e27604.

69. Lakoh S, Adekanmbi O, Jiba DF, Deen GF, Gashau W, Sevalie S, et al. Antibiotic use among hospitalized adult patients in a setting with limited laboratory infrastructure in Freetown Sierra Leone, 2017-2018. Int J Infect Dis. 2020;90:71-6.

70. Porto A, Goossens H, Versporten A, Costa SF. Global point prevalence survey of antimicrobial consumption in Brazilian hospitals. J Hosp Infect. 2020;104(2):165-71.

71. Resurreccion-Delgado C, Chiappe-Gonzalez A, Bolarte-Espinoza J, Martinez-Dionisio L, Munante-Meneses R, Vicente-Lozano Y, et al. Use of antibiotics in inpatients from a national hospital in Lima, Peru. Rev Peru Med Exp Salud Publica. 2020;37(4):620-6.

72. Fowotade A, Fasuyi T, Aigbovo O, Versporten A, Adekanmbi O, Akinyemi O, et al. Point prevalence survey of antimicrobial prescribing in a Nigerian hospital: findings and implications on antimicrobial resistance. West Afr J Med. 2020;37(3):216-20.

73. Tribble AC, Lee BR, Flett KB, Handy LK, Gerber JS, Hersh AL, et al. Appropriateness of antibiotic prescribing in United States children's hospitals: a national point prevalence survey. Clin Infect Dis. 2020;71(8):e226-34.

74. Hafeez M, Saleem Z, Bukhari NA, Hussain K, Shamim R, Hussain A, et al. Off-label antibiotic use in a specialized children care hospital in Punjab, Pakistan: findings and implications. J Infect Dev Countr. 2020;14(5):540-4.
75. Komagamine J, Kobayashi M, Mori T. Prevalence of and rationale for antimicrobial prescription during ambulatory care visits in Japan: a prospective, multicentre, cross-sectional study. Bmj Open. 2020;10(8):e39329.
76. Masich AM, Vega AD, Callahan P, Herbert A, Fwoloshi S, Zulu PM, et al. Antimicrobial usage at a large teaching hospital in Lusaka, Zambia. Plos One. 2020;15(2):e228555.
77. Frenette C, Sperlea D, German GJ, Afra K, Boswell J, Chang S, et al. The 2017 global point prevalence survey of antimicrobial consumption and resistance in Canadian hospitals. Antimicrob Resist in. 2020;9(1):104.
78. Roberts AA, Fajolu I, Oshun P, Osuagwu C, Awofeso O, Temiye E, et al. Feasibility study of prospective audit, intervention and feedback as an antimicrobial stewardship strategy at the Lagos University Teaching Hospital. Niger Postgrad Med J. 2020;27(1):54-8.
79. Tham D, Abubakar U, Tangiisuran B. Prevalence and predictors of antibiotic use among children visiting the emergency department in a tertiary hospital in Malaysia. Eur J Pediatr. 2020;179(5):743-8.
80. Maina M, Mwaniki P, Odira E, Kiko N, McKnight J, Schultsz C, et al. Antibiotic use in Kenyan public hospitals: prevalence, appropriateness and link to guideline availability. Int J Infect Dis. 2020;99:10-8.
81. Griffith HG, Dantuluri K, Thurm C, Williams DJ, Banerjee R, Howard LM, et al. Considerable variability in antibiotic use among US children's hospitals in 2017-2018. Infect Cont Hosp Ep. 2020;41(5):571-8.
82. Chautrakarn S, Anugulruengkitt S, Puthanakit T, Rattananupong T, Hiransuthikul N. Antimicrobial prescription patterns in a tertiary-care pediatric unit in Thailand. Pediatr Int. 2020;62(6):683-7.
83. Khan Z, Ahmed N, Zafar S, Ullah KF, Ur RA, Parreiras MM, et al. A pilot study on the rational use of medicines in four tertiary care hospitals through validated World Health Organization prescribing drugs indicators. Ann Ig Med Prev Comu. 2020;32(4):368-75.
84. Yilma Z, Mekonnen T, Siraj EA, Agmassie Z, Yehualaw A, Debasu Z, et al. Assessment of prescription completeness and drug use pattern in Tibebe-Ghion Comprehensive Specialized Hospital, Bahir Dar, Ethiopia. Biomed Res Int. 2020;2020:8842515.
85. Yilma Z, Liben M. Assessment of drug prescription pattern in Mekelle General Hospital, Mekelle, Ethiopia, using World Health Organization prescribing indicators. Biomed Res Int. 2020;2020:3809157.
86. Nepal A, Hendrie D, Robinson S, Selvey LA. Analysis of patterns of antibiotic prescribing in public health facilities in Nepal. J Infect Dev Countr. 2020;14(1):18-27.
87. Frost HM, Knepper BC, Shihadeh KC, Jenkins TC. A novel approach to evaluate antibiotic utilization across the spectrum of inpatient and ambulatory care and

implications for prioritization of antibiotic stewardship efforts. Clin Infect Dis. 2020;70(8):1675-82.
88. Miao R, Wan C, Wang Z, Zhu Y, Zhao Y, Zhang L, et al. Inappropriate antibiotic prescriptions among pediatric inpatients in different type hospitals. Medicine. 2020;99(2):e18714.
89. Bimba HV, Roy V, Batta A, Daga MK. Drug utilization, rationality, and cost analysis of antimicrobial medicines in a tertiary care teaching hospital of Northern India: a prospective, observational study. Indian J Pharmacol. 2020;52(3):179-88.
90. Seni J, Mapunjo SG, Wittenauer R, Valimba R, Stergachis A, Werth BJ, et al. Antimicrobial use across six referral hospitals in Tanzania: a point prevalence survey. Bmj Open. 2020;10(12):e42819.
91. Karabay O, Ince N, Aypak A, Guclu E, Bodur H. Antibiotic usage in hospitalized patients: a one-day point prevalence study. J Chemotherapy. 2020;32(4):188-92.
92. Abubakar U. Antibiotic use among hospitalized patients in northern Nigeria: a multicenter point-prevalence survey. Bmc Infect Dis. 2020;20(1):86.
93. Horumpende PG, Mshana SE, Mouw EF, Mmbaga BT, Chilongola JO, de Mast Q. Point prevalence survey of antimicrobial use in three hospitals in North-Eastern Tanzania. Antimicrob Resist in. 2020;9(1):149.
94. Vandael E, Latour K, Goossens H, Magerman K, Drapier N, Catry B, et al. Point prevalence survey of antimicrobial use and healthcare-associated infections in Belgian acute care hospitals: results of the Global-PPS and ECDC-PPS 2017. Antimicrob Resist in. 2020;9(1):13.
95. Mohammed ZA, Mukhopadhyay C, Varma M, Kalwaje EV. Identifying opportunities for antimicrobial stewardship through a point prevalence survey in an Indian tertiary-care teaching hospital. J Glob Antimicrob Re. 2020;23:315-20.
96. Jaggi P, Hamdy RF, Lee B, Hersh AL, Gerber JS, Sharland M, et al. Use of antimicrobial agents in hospitalized children for noninfectious indications. J Pediat Inf Dis Soc. 2020;9(4):490-3.
97. Thamlikitkul V, Rattanaumpawan P, Sirijatuphat R, Wangchinda W. Integrated one-day surveillance of antimicrobial use, antimicrobial consumption, antimicrobial resistance, healthcare-associated infection, and antimicrobial resistance burden among hospitalized patients in Thailand. J Infection. 2020;81(1):98-106.
98. Al-Tawfiq JA, Al-Homoud AH. Pattern of systemic antibiotic use among hospitalized patients in a general hospital in Saudi Arabia. Travel Med Infect Di. 2020;36:101605.
99. Antonioli P, Bolognesi N, Valpiani G, Morotti C, Bernardini D, Bravi F, et al. A 2-year point-prevalence surveillance of healthcare-associated infections and antimicrobial use in Ferrara University Hospital, Italy. Bmc Infect Dis. 2020;20(1):75.
100. Loftus MJ, Curtis SJ, Naidu R, Cheng AC, Jenney A, Mitchell BG, et al. Prevalence of healthcare-associated infections and antimicrobial use among inpatients in a tertiary hospital in Fiji: a point prevalence survey. Antimicrob Resist in. 2020;9(1):146.
101. Gardiner SJ, Basevi AB, Hamilton NL, Metcalf SC, Chambers ST, Withington SG, et al. Point prevalence surveys of antimicrobial use in adult inpatients at Canterbury

District Health Board Hospitals. New Zeal Med J. 2020;133(1525):18-33.
102. Xu JJ, Gao J, Guo JH, Song LL. Analysis of antibiotic treatment of children in a Shanghai tertiary hospital based on point prevalence surveys. Bmc Infect Dis. 2020;20(1):804.
103. Olaru ID, Meierkord A, Godman B, Ngwenya C, Fitzgerald F, Dondo V, et al. Assessment of antimicrobial use and prescribing practices among pediatric inpatients in Zimbabwe. J Chemotherapy. 2020;32(8):456-9.
104. Wang CN, Huttner BD, Magrini N, Cheng Y, Tong J, Li S, et al. Pediatric antibiotic prescribing in China according to the 2019 World Health Organization Access, Watch, and Reserve (AWaRe) antibiotic categories. J Pediatr-Us. 2020;220:125-31.
105. Zhang M, Ma XY, Feng ZQ, Gao L. Survey of antibiotic use among hospitalised children in a hospital in Northeast China over a 4-year period. J Spec Pediatr Nurs. 2020;25(2):e12282.
106. Cusini A, Rampini SK, Bansal V, Ledergerber B, Kuster SP, Ruef C, et al. Different patterns of inappropriate antimicrobial use in surgical and medical units at a tertiary care hospital in Switzerland: a prevalence survey. Plos One. 2010;5(11):e14011.
107. Black E, Neville H, Losier M, Harrison M, Abbass K, Slayter K, et al. Antimicrobial use at acute care hospitals in Nova Scotia: a point prevalence survey. Can J Hosp Pharm. 2018;71(4):234-42.
108. Oduyebo OO, Olayinka AT, Iregbu KC, Versporten A, Ogunsola FT. A point prevalence survey of antimicrobial prescribing in four Nigerian tertiary hospitals. Ann Trop Pathol. 2017;8:42-6.
109. Monteiro L, Chauque A, Barros MP, Ira TR. Determinants of antibiotic prescription in paediatric patients: the case of two hospitals in Maputo, Mozambique. S Afr J Child Health. 2017;11(3):109-11.
110. Nair V, Sharma D, Sahni AK, Grover N, Shankar S, Jaiswal SS, et al. Antimicrobial use and antimicrobial resistance in nosocomial pathogens at a tertiary care hospital in Pune. Med J Armed Forces India. 2015;71(2):112-9.
111. Singh S, Jose T, Versporten A. A point prevalence surveillance study from pediatric and neonatal specialty hospitals in India. J Pediat Inf Dis-Ger. 2014;9(3):151-5.
112. Denny KJ, Gartside JG, Alcorn K, Cross JW, Maloney S, Keijzers G. Appropriateness of antibiotic prescribing in the emergency department. J Antimicrob Chemoth. 2019;74(2):515-20.
113. Sikkens JJ, Gerritse SL, Peters E, Kramer M, van Agtmael MA. The 'morning dip' in antimicrobial appropriateness: circumstances determining appropriateness of antimicrobial prescribing. J Antimicrob Chemoth. 2018;73(6):1714-20.
114. Croche SB, Campos AE, Sanchez CA, Marcos FL, Diaz FI, Vargas JC, et al. Appropriateness of antibiotic prescribing in paediatric patients in a hospital emergency department. An Pediatr (Engl Ed). 2018;88(5):259-65.

115. Labi AK, Obeng-Nkrumah N, Nartey ET, Bjerrum S, Adu-Aryee NA, Ofori-Adjei YA, et al. Antibiotic use in a tertiary healthcare facility in Ghana: a point prevalence survey. Antimicrob Resist in. 2018;7:15.
116. Olivier C, Kunneke H, O'Connell N, Von Delft E, Wates M, Dramowski A. Healthcare-associated infections in paediatric and neonatal wards: a point prevalence survey at four South African hospitals. Samj S Afr Med J. 2018;108(5):418-22.
117. Labi AK, Obeng-Nkrumah N, Sunkwa-Mills G, Bediako-Bowan A, Akufo C, Bjerrum S, et al. Antibiotic prescribing in paediatric inpatients in Ghana: a multi-centre point prevalence survey. Bmc Pediatr. 2018;18(1):391.
118. Zhang JS, Liu G, Zhang WS, Shi HY, Lu G, Zhao CA, et al. Antibiotic usage in Chinese children: a point prevalence survey. World J Pediatr. 2018;14(4):335-43.
119. Shrestha B, Dixit SM. The assessment of drug use pattern using WHO prescribing indicators. J Nepal Health Res Counc. 2018;16(3):279-84.
120. Mengistu G, Misganaw D, Tsehay T, Alemu BK, Bogale K. Assessment of drug use pattern using WHO core prescribing indicators at outpatient settings of governmental hospitals in Dessie town. Drug Healthc Patient. 2020;12:237-44.
121. Plachouras D, Karki T, Hansen S, Hopkins S, Lyytikainen O, Moro ML, et al. Antimicrobial use in European acute care hospitals: results from the second point prevalence survey (PPS) of healthcare-associated infections and antimicrobial use, 2016 to 2017. Eurosurveillance. 2018;23(46):1800393.
122. Al-Taani GM, Scott M, Farren D, Gilmore F, Mccullagh B, Hibberd C, et al. Longitudinal point prevalence survey of antibacterial use in Northern Ireland using the European Surveillance of Antimicrobial Consumption (ESAC) PPS and Global-PPS tool. Epidemiol Infect. 2018;146(8):985-90.
123. Gutema G, Håkonsen H, Engidawork E, Toverud EL. Multiple challenges of antibiotic use in a large hospital in Ethiopia - a ward-specific study showing high rates of hospital-acquired infections and ineffective prophylaxis. Bmc Health Serv Res. 2018;18(1):326.
124. Sticchi C, Alberti M, Artioli S, Assensi M, Baldelli I, Battistini A, et al. Regional point prevalence study of healthcare-associated infections and antimicrobial use in acute care hospitals in Liguria, Italy. J Hosp Infect. 2018;99(1):8-16.
125. Elhajji FD, Al-Taani GM, Anani L, Al-Masri S, Abdalaziz H, Qabba'H SH, et al. Comparative point prevalence survey of antimicrobial consumption between a hospital in Northern Ireland and a hospital in Jordan. Bmc Health Serv Res. 2018;18(1):849.
126. Koopmans LR, Finlayson H, Whitelaw A, Decloedt EH, Dramowski A. Paediatric antimicrobial use at a South African hospital. Int J Infect Dis. 2018;74:16-23.
127. Okoth C, Opanga S, Okalebo F, Oluka M, Baker KA, Godman B. Point prevalence survey of antibiotic use and resistance at a referral hospital in Kenya: findings and implications. Hosp Pract (1995). 2018;46(3):128-36.
128. Metsini A, Vazquez M, Sommerstein R, Marschall J, Voide C, Troillet N, et al. Point prevalence of healthcare-associated infections and antibiotic use in three large Swiss acute-care hospitals. Swiss Med Wkly. 2018;148:w14617.
129. Morioka H, Nagao M, Yoshihara S, Ohge H, Kasahara K, Shigemoto N, et al. The first multi-centre point-prevalence survey in four Japanese university hospitals. J

Hosp Infect. 2018;99(3):325-31.
130. Amaha ND, Berhe YH, Kaushik A. Assessment of inpatient antibiotic use in Halibet National Referral Hospital using WHO indicators: a retrospective study. Bmc Res Notes. 2018;11(1):904.
131. Sviestina I, Mozgis D. Observational study of antibiotic usage at the Children's Clinical University Hospital in Riga, Latvia. Medicina-Lithuania. 2018;54(5):74.
132. Yan K, Xue M, Ye D, Yang C, Chang J, Jiang M, et al. Antibiotic prescribing practices in secondary and tertiary hospitals in Shaanxi province, western China, 2013-2015. Plos One. 2018;13(12):e207229.
133. Umar LW, Isah A, Musa S, Umar B. Prescribing pattern and antibiotic use for hospitalized children in a northern Nigerian teaching hospital. Ann Afr Med. 2018;17(1):26-32.
134. Chaw PS, Schlinkmann KM, Raupach-Rosin H, Karch A, Pletz MW, Huebner J, et al. Antibiotic use on paediatric inpatients in a teaching hospital in the Gambia, a retrospective study. Antimicrob Resist in. 2018;7:82.
135. Hussain S, Yadav SS, Sawlani KK, Khattri S. Assessment of drug prescribing pattern using world health organization indicators in a tertiary care teaching hospital. Indian J Public Hlth. 2018;62(2):156-8.
136. Cairns S, Gibbons C, Milne A, King H, Llano M, MacDonald L, et al. Results from the third Scottish national prevalence survey: is a population health approach now needed to prevent healthcare-associated infections? J Hosp Infect. 2018;99(3):312-7.
137. Wang S, Guan L, Dong C, Ji Y. Evaluation of the drug effect and rational use of antiseptic drugs in outpatient and emergency department of hospital. Pak J Pharm Sci. 2018;31(4(Special)):1701-5.
138. Wang J, Hu J, Harbarth S, Pittet D, Zhou M, Zingg W. Burden of healthcare-associated infections in China: results of the 2015 point prevalence survey in Dong Guan city. J Hosp Infect. 2017;96(2):132-8.
139. Chen Y, Zhao JY, Shan X, Han XL, Tian SG, Chen FY, et al. A point-prevalence survey of healthcare-associated infection in fifty-two Chinese hospitals. J Hosp Infect. 2017;95(1):105-11.
140. Ioannou P, Astrinaki E, Vitsaxaki E, Bolikas E, Christofaki D, Salvaraki A, et al. A point prevalence survey of healthcare-associated infections and antimicrobial use in public acute care hospitals in Crete, Greece. Antibiotics-Basel. 2022;11(9).
141. Zeng L, Hu D, Choonara I, Mu D, Zhang L, Li X, et al. A prospective study of the use of antibiotics in the emergency department of a Chinese university hospital. Int J Pharm Pract. 2017;25(1):89-92.
142. Kebede HK, Gesesew HA, Woldehaimanot TE, Goro KK. Antimicrobial use in paediatric patients in a teaching hospital in Ethiopia. Plos One. 2017;12(3):e173290.
143. Goycochea-Valdivia WA, Moreno-Ramos F, Pano-Pardo JR, Aracil-Santos FJ, Baquero-Artigao F, Del RT, et al. Identifying priorities to improve paediatric in-hospital

antimicrobial use by cross-sectional evaluation of prevalence and appropriateness of prescription. Enferm Infec Micr Cl. 2017;35(9):556-62.
144. Sisay M, Mengistu G, Molla B, Amare F, Gabriel T. Evaluation of rational drug use based on World Health Organization core drug use indicators in selected public hospitals of eastern Ethiopia: a cross sectional study. Bmc Health Serv Res. 2017;17(1):161.
145. Cai Y, Venkatachalam I, Tee NW, Tan TY, Kurup A, Wong SY, et al. Prevalence of healthcare-associated infections and antimicrobial use among adult inpatients in Singapore acute-care hospitals: results from the first national point prevalence survey. Clin Infect Dis. 2017;64(suppl_2):S61-7.
146. Guclu E, Ogutlu A, Karabay O, Demirdal T, Erayman I, Hosoglu S, et al. Antibiotic consumption in Turkish hospitals; a multi-centre point prevalence study. J Chemotherapy. 2017;29(1):19-24.
147. Liao XP, Chipenda-Dansokho S, Lewin A, Abdelouahab N, Wei SQ. Advanced neonatal medicine in China: a national baseline database. Plos One. 2017;12(1):e169970.
148. Fleming-Dutra KE, Hersh AL, Shapiro DJ, Bartoces M, Enns EA, File TJ, et al. Prevalence of inappropriate antibiotic prescriptions among US ambulatory care visits, 2010-2011. Jama-J Am Med Assoc. 2016;315(17):1864-73.
149. Lee ML, Cho CY, Hsu CL, Chen CJ, Chang LY, Lee YS, et al. Recent trends in antibiotic prescriptions for acute respiratory tract infections in pediatric ambulatory care in Taiwan, 2000-2009: a nationwide population-based study. J Microbiol Immunol. 2016;49(4):554-60.
150. Versporten A, Bielicki J, Drapier N, Sharland M, Goossens H. The Worldwide Antibiotic Resistance and Prescribing in European Children (ARPEC) point prevalence survey: developing hospital-quality indicators of antibiotic prescribing for children. J Antimicrob Chemoth. 2016;71(4):1106-17.
151. Morioka H, Hirabayashi A, Iguchi M, Tomita Y, Kato D, Sato N, et al. The first point prevalence survey of health care-associated infection and antimicrobial use in a Japanese university hospital: a pilot study. Am J Infect Control. 2016;44(7):e119-23.
152. Fahimzad A, Eydian Z, Karimi A, Shiva F, Sayyahfar S, Kahbazi M, et al. Surveillance of antibiotic consumption point prevalence survey 2014: antimicrobial prescribing in pediatrics wards of 16 Iranian hospitals. Arch Iran Med. 2016;19(3):204-9.
153. Gharbi M, Doerholt K, Vergnano S, Bielicki JA, Paulus S, Menson E, et al. Using a simple point-prevalence survey to define appropriate antibiotic prescribing in hospitalised children across the UK. Bmj Open. 2016;6(11):e12675.
154. Ren N, Zhou P, Wen X, Li C, Huang X, Guo Y, et al. Point prevalence survey of antimicrobial use in Chinese hospitals in 2012. Am J Infect Control. 2016;44(3):332-9.
155. De Luca M, Dona D, Montagnani C, Lo VA, Romanengo M, Tagliabue C, et al. Antibiotic prescriptions and prophylaxis in Italian children. Is it time to change? Data from the ARPEC project. Plos One. 2016;11(5):e154662.
156. Stefkovicova M, Litvova S, Melus V, Kristufkova Z, Brazinova A. Point prevalence study of antimicrobial usage in acute care hospitals in the Slovak Republic. J Hosp Infect. 2016;93(4):403-9.

157. Kiguba R, Karamagi C, Bird SM. Extensive antibiotic prescription rate among hospitalized patients in Uganda: but with frequent missed-dose days. J Antimicrob Chemoth. 2016;71(6):1697-706.
158. Bilal AI, Osman ED, Mulugeta A. Assessment of medicines use pattern using World Health Organization's prescribing, patient care and health facility indicators in selected health facilities in eastern Ethiopia. Bmc Health Serv Res. 2016;16:144.
159. Knezevic B, Sprigg D, Seet J, Trevenen M, Trubiano J, Smith W, et al. The revolving door: antibiotic allergy labelling in a tertiary care centre. Intern Med J. 2016;46(11):1276-83.
160. Segagni LL, Blacky A, Starzengruber P, Diab-Elschahawi M, Wrba T, Presterl E. A national point prevalence study on healthcare-associated infections and antimicrobial use in Austria. Wien Klin Wochenschr. 2016;128(3-4):89-94.
161. Antonioli P, Manzalini MC, Stefanati A, Bonato B, Verzola A, Formaglio A, et al. Temporal trends of healthcare associated infections and antimicrobial use in 2011-2013, observed with annual point prevalence surveys in Ferrara University Hospital, Italy. J Prev Med Hyg. 2016;57(3):E135-41.
162. Vercheval C, Gillet M, Maes N, Albert A, Frippiat F, Damas P, et al. Quality of documentation on antibiotic therapy in medical records: evaluation of combined interventions in a teaching hospital by repeated point prevalence survey. Eur J Clin Microbiol. 2016;35(9):1495-500.
163. Marani A, Napoli C, Berdini S, Montesano M, Ferretti F, Di Ninno F, et al. Point prevalence surveys on healthcare acquired infections in medical and surgical wards of a teaching hospital in Rome. Ann Ig Med Prev Comu. 2016;28(4):274-81.
164. Zhang Y, Zhang J, Wei D, Yang Z, Wang Y, Yao Z. Annual surveys for point-prevalence of healthcare-associated infection in a tertiary hospital in Beijing, China, 2012-2014. Bmc Infect Dis. 2016;16:161.
165. Atif M, Azeem M, Saqib A, Scahill S. Investigation of antimicrobial use at a tertiary care hospital in southern Punjab, Pakistan using WHO methodology. Antimicrob Resist in. 2017;6:41.
166. Atif M, Sarwar MR, Azeem M, Umer D, Rauf A, Rasool A, et al. Assessment of WHO/INRUD core drug use indicators in two tertiary care hospitals of Bahawalpur, Punjab, Pakistan. J Pharm Policy Pract. 2016;9:27.
167. Akhtar M. Drug prescribing practices in paediatric department of a north Indian university teaching hospital. Asian J Pharm Clin Res. 2012;1(5):146-9.
168. Henry CN. Drug use pattern with standard indicators in Jos University Teaching Hospital Nigeria. West African Journal of Pharmacy. 2013;4(12):88-93.
169. Angamo MT, Wabe NT, Raju NJ. Assessment of patterns of drug use by using World Health Organization's prescribing, patient care and health facility indicators in selected health facilities in southwest Ethiopia. Journal of applied pharmaceutical science. 2011;1(7):62-6.
170. Woldu MA. Retrospective study of the pattern of antibiotic use in Hawassa University Referral Hospital pediatric ward, southern Ethiopia. Journal of Applied Pharmaceutical Science. 2013;2(3):93-8.

171. Al-Niemat SI, Aljbouri TM, Goussous LS, Efaishat RA, Salah RK. Antibiotic prescribing patterns in outpatient emergency clinics at Queen Rania Al Abdullah II Children's Hospital, Jordan, 2013. Oman Med J. 2014;29(4):250-4.
172. Mohajer KA, Al-Yami SM, Al-Jeraisy MI, Abolfotouh MA. Antibiotic prescribing in a pediatric emergency setting in central Saudi Arabia. Saudi Med J. 2011;32(2):197-8.
173. Wang H, Li N, Zhu H, Xu S, Lu H, Feng Z. Prescription pattern and its influencing factors in Chinese county hospitals: a retrospective cross-sectional study. Plos One. 2013;8(5):e63225.
174. Osowicki J, Gwee A, Noronha J, Britton PN, Isaacs D, Lai TB, et al. Australia-wide point prevalence survey of antimicrobial prescribing in neonatal units: how much and how good? Pediatr Infect Dis J. 2015;34(8):e185-90.
175. Amadeo B, Zarb P, Muller A, Drapier N, Vankerckhoven V, Rogues AM, et al. European Surveillance of Antibiotic Consumption (ESAC) point prevalence survey 2008: paediatric antimicrobial prescribing in 32 hospitals of 21 European countries. J Antimicrob Chemoth. 2010;65(10):2247-52.
176. Magill SS, Edwards JR, Beldavs ZG, Dumyati G, Janelle SJ, Kainer MA, et al. Prevalence of antimicrobial use in US acute care hospitals, May-September 2011. Jama-J Am Med Assoc. 2014;312(14):1438-46.
177. Aldeyab MA, Kearney MP, McElnay JC, Magee FA, Conlon G, MacIntyre J, et al. A point prevalence survey of antibiotic use in four acute-care teaching hospitals utilizing the European Surveillance of Antimicrobial Consumption (ESAC) audit tool. Epidemiol Infect. 2012;140(9):1714-20.
178. Talaat M, Saied T, Kandeel A, El-Ata GA, El-Kholy A, Hafez S, et al. A point prevalence survey of antibiotic use in 18 hospitals in Egypt. Antibiotics-Basel. 2014;3(3):450-60.
179. Akhloufi H, Streefkerk RH, Melles DC, de Steenwinkel JE, Schurink CA, Verkooijen RP, et al. Point prevalence of appropriate antimicrobial therapy in a Dutch university hospital. Eur J Clin Microbiol. 2015;34(8):1631-7.
180. Sinatra I, Carubia L, Marchese V, Aprea L, D'Alessandro N, Mammina C, et al. Prevalence survey of healthcare-associated infections and antimicrobial use at the University Hospital "Paolo Giaccone", Palermo, Italy. J Prev Med Hyg. 2013;54(4):200-4.
181. Gizework A, Seyfe AA. Assessment of the pattern of antibiotics use in pediatrics ward of Dessie Referral Hospital, North East Ethiopia. International Journal of Medicine and Medical Sciences. 2015;7(1):1-7.
182. Gandra S, Singh SK, Jinka DR, Kanithi R, Chikkappa AK, Sharma A, et al. Point prevalence surveys of antimicrobial use among hospitalized children in six hospitals in India in 2016. Antibiotics-Basel. 2017;6(3).
183. Nsofor CA, Amadi ES, Obijuru CE, Ohalete CV, Ukwandu N. Prevalence of antimicrobial use in major hospitals in Owerri, Nigeria. EC Microbiology. 2016;3(5):522-7.

184. Mulwa NC, Osanjo GO, Ndwigah S, Kaburi AN, Muriuki G. Patterns of prescribing practices in Makueni County Referral Hospital, Kenya. The African Journal of Pharmacology and Therapeutics. 2015;4(4):161-8.
185. Bashrahil KA. Indicators of rational drug use and health services in Hadramout, Yemen. E Mediterr Health J. 2010;16(2):151-5.
186. Mudenda W, Chikatula E, Chambula E, Mwanashimbala B, Chikuta M, Masaninga F, et al. Prescribing patterns and medicine use at the University Teaching Hospital, Lusaka, Zambia. Zambia Medical Association. 2016;43(2):94-102.
187. Joda AE, Aderemi-Williams RI. A comparative study of prescribing patterns in two tertiary care teaching hospitals in Lagos, Nigeria. International Journal of Pharmacy and Pharmacology.;2(1):41-6.
188. Choudhury DK, Bezbaruah BK. Antibiotic prescriptions pattern in paediatric in-patient department gauhati medical college and hospital, Guwahati. Journal of Applied Pharmaceutical Science. 2013;3(8):144-8.
189. José MW, Jean-Marie LI, Divine MM, Sabine KK, Takaisi- K. Point prevalence study of antibiotic use in hospitals in Butembo. International Journal of Medicine and Medical Sciences. 2016;8(12):133-9.
190. Assen A, Abrha S. Assessment of drug prescribing pattern in Dessie Referral Hospital, Dessie. International Journal of Pharma Sciences & Research. 2014;5(11):777-81.
191. Li C, Ren N, Wen X, Zhou P, Huang X, Gong R, et al. Changes in antimicrobial use prevalence in China: results from five point prevalence studies. Plos One. 2013;8(12):e82785.
192. Versporten A, Sharland M, Bielicki J, Drapier N, Vankerckhoven V, Goossens H. The antibiotic resistance and prescribing in European children project: a neonatal and pediatric antimicrobial web-based point prevalence survey in 73 hospitals worldwide. Pediatr Infect Dis J. 2013;32(6):e242-53.
193. Sviestina I, Aston J, Lorrot M, Mozgis D. A comparison of antibiotic use in three specialist paediatric hospitals in France, Latvia and the UK. Eur J Hosp Pharm. 2015;22(3).
194. Sviestina I, Aston J, Mozgis D. Comparison of antimicrobial prescribing between two specialist paediatric centres in the UK and Latvia. Eur J Hosp Pharm. 2013;20(3):180-4.
195. Bergicho M, Mohammed MA, Wabe N. Assessment of the pattern of drug prescribing in pediatrics ward in tertiary setting hospital in Addis Ababa, Ethiopia. Gaziantep Medical Journal. 2012;18:61-5.
196. Xie DS, Xiang LL, Hu Q, Fu XY, Wang HF, Lai RP, et al. Antibiotic use in Chinese hospitals: a multicenter point-prevalence study. Public Health. 2015;129(5):576-8.
197. Fadare J, Olatunya O, Oluwayemi O, Ogundare O. Drug prescribing pattern for under-fives in a paediatric clinic in south-western Nigeria. Ethiop J Health Sci. 2015;25(1):73-8.

198. Xie DS, Xiang LL, Li R, Hu Q, Luo QQ, Xiong W. A multicenter point-prevalence survey of antibiotic use in 13 Chinese hospitals. J Infect Public Heal. 2015;8(1):55-61.
199. Alfandari S, Robert J, Pean Y, Rabaud C, Bedos JP, Varon E, et al. Antibiotic use and good practice in 314 French hospitals: the 2010 SPA2 prevalence study. Med Maladies Infect. 2015;45(11-12):475-80.
200. van Spreuwel PC, Blok H, Langelaar MF, Kullberg BJ, Mouton JW, Natsch S. Identifying targets for quality improvement in hospital antibiotic prescribing. Neth J Med. 2015;73(4):161-8.
201. Cotta MO, Robertson MS, Upjohn LM, Marshall C, Liew D, Buising KL. Using periodic point-prevalence surveys to assess appropriateness of antimicrobial prescribing in Australian private hospitals. Intern Med J. 2014;44(3):240-6.
202. Sviestina I, Mozgis D. Antimicrobial usage among hospitalized children in Latvia: a neonatal and pediatric antimicrobial point prevalence survey. Medicina-Lithuania. 2014;50(3):175-81.
203. Osowicki J, Gwee A, Noronha J, Palasanthiran P, McMullan B, Britton PN, et al. Australia-wide point prevalence survey of the use and appropriateness of antimicrobial prescribing for children in hospital. Med J Australia. 2014;201(11):657-62.
204. Bozkurt F, Kaya S, Tekin R, Gulsun S, Deveci O, Dayan S, et al. Analysis of antimicrobial consumption and cost in a teaching hospital. J Infect Public Heal. 2014;7(2):161-9.
205. Muyu G, Mbakaya C, Makokha A. Outpatient prescribing practices at Mbagathi District Hospital-Nairobi county. East Afr Med J. 2013;90(12):387-95.
206. Desalegn AA. Assessment of drug use pattern using WHO prescribing indicators at Hawassa University Teaching and Referral Hospital, south Ethiopia: a cross-sectional study. Bmc Health Serv Res. 2013;13(1):170.
207. Sozen H, Gonen I, Sozen A, Kutlucan A, Kalemci S, Sahan M. Application of ATC/DDD methodology to evaluate of antibiotic use in a general hospital in Turkey. Ann Clin Microb Anti. 2013;12:23.
208. Behnke M, Hansen S, Leistner R, Diaz LA, Gropmann A, Sohr D, et al. Nosocomial infection and antibiotic use: a second national prevalence study in Germany. Dtsch Arztebl Int. 2013;110(38):627-33.
209. Mabiala BJ, Ollandzobo IL, Mbika CA, Moyen G. Prescription of antibiotic drugs for children at the Brazzaville University Hospital Center (Congo). Med Sante Trop. 2013;23(2):189-92.
210. Borras NC, Hernandez BS, Garcia GJ. Prescribing of antibiotics in patients admitted from emergency departments: a multicenter study. An Pediatr. 2013;79(1):15-20.
211. Zarb P, Coignard B, Griskeviciene J, Muller A, Vankerckhoven V, Weist K, et al. The European Centre for Disease Prevention and Control (ECDC) pilot point prevalence survey of healthcare-associated infections and antimicrobial use. Eurosurveillance. 2012;17(46):20316.

212. Thu TA, Rahman M, Coffin S, Harun-Or-Rashid M, Sakamoto J, Hung NV. Antibiotic use in Vietnamese hospitals: a multicenter point-prevalence study. Am J Infect Control. 2012;40(9):840-4.
213. Ingram PR, Seet JM, Budgeon CA, Murray R. Point-prevalence study of inappropriate antibiotic use at a tertiary Australian hospital. Intern Med J. 2012;42(6):719-21.
214. Robert J, Pean Y, Varon E, Bru JP, Bedos JP, Bertrand X, et al. Point prevalence survey of antibiotic use in French hospitals in 2009. J Antimicrob Chemoth. 2012;67(4):1020-6.
215. Levy ER, Swami S, Dubois SG, Wendt R, Banerjee R. Rates and appropriateness of antimicrobial prescribing at an academic children's hospital, 2007-2010. Infect Cont Hosp Ep. 2012;33(4):346-53.
216. Sharma M, Eriksson B, Marrone G, Dhaneria S, Lundborg CS. Antibiotic prescribing in two private sector hospitals; one teaching and one non-teaching: a cross-sectional study in Ujjain, India. Bmc Infect Dis. 2012;12(1):155.
217. Baktygul K, Marat B, Ashirali Z, Harun-Or-rashid M, Sakamoto J. An assessment of antibiotics prescribed at the secondary health-care level in the Kyrgyz Republic. Nagoya J Med Sci. 2011;73(3-4):157-68.
218. Carneiro M, Ferraz T, Bueno M, Koch BE, Foresti C, Lena VF, et al. Antibiotic prescription in a teaching hospital: a brief assessment. Rev Assoc Med Bras. 2011;57(4):414-7.
219. Pathak A, Mahadik K, Dhaneria SP, Sharma A, Eriksson B, Lundborg CS. Antibiotic prescribing in outpatients: hospital and seasonal variations in Ujjain, India. Scand J Infect Dis. 2011;43(6-7):479-88.
220. Xie DS, Xiong W, Xiang LL, Fu XY, Yu YH, Liu L, et al. Point prevalence surveys of healthcare-associated infection in 13 hospitals in Hubei province, China, 2007-2008. J Hosp Infect. 2010;76(2):150-5.
221. Ceyhan M, Yildirim I, Ecevit C, Aydogan A, Ornek A, Salman N, et al. Inappropriate antimicrobial use in Turkish pediatric hospitals: a multicenter point prevalence survey. Int J Infect Dis. 2010;14(1):e55-61.
222. Willemsen I, van der Kooij T, van Benthem B, Wille J, Kluytmans J. Appropriateness of antimicrobial therapy: a multicentre prevalence survey in the Netherlands, 2008-2009. Eurosurveillance. 2010;15(46):19715.
223. Kolyva S, Gkentzi D, Koulouri A, Dimitriou G. Antibiotic prescribing in the pediatric emergency department. J Chemotherapy. 2017;29(4):257-60.
224. Larru B, Sulieman SE, Localio R, Ross RK, Sharland M, Zaoutis TE, et al. Frontline clinician knowledge of antimicrobial prescribing in an academic tertiary children's hospital: a point prevalence study. J Pediat Inf Dis Soc. 2016;5(4):462-4.
225. Sharma S, Bowman C, Alladin-Karan B, Singh N. Antibiotic prescribing patterns in the pediatric emergency department at Georgetown Public Hospital Corporation: a retrospective chart review. Bmc Infect Dis. 2016;16(1):170.

226. Aly NY, Omar AA, Badawy DA, Al-Mousa HH, Sadek AA. Audit of physicians' adherence to the antibiotic policy guidelines in Kuwait. Med Prin Pract. 2012;21(4):310-7.
227. Gailiene G, Gierasimovic Z, Petruseviciene D, Macijauskiene A. The prevalence of health care-associated infections and risk factors in a university hospital. Medicina-Lithuania. 2012;48(8):399-403.
228. Askarian M, Yadollahi M, Assadian O. Point prevalence and risk factors of hospital acquired infections in a cluster of university-affiliated hospitals in Shiraz, Iran. J Infect Public Heal. 2012;5(2):169-76.
229. Dryden M, Saeed K, Townsend R, Winnard C, Bourne S, Parker N, et al. Antibiotic stewardship and early discharge from hospital: impact of a structured approach to antimicrobial management. J Antimicrob Chemoth. 2012;67(9):2289-96.
230. Gomez-Gomez J, Garcia-Vazquez E, Bonillo C, Hernandez-Torres A, Canteras-Jordana M. Use of antibiotics at a university clinic hospital: effect of protocolized antibiotic treatment in the evolution of hospital patients with infections. Rev Esp Quim. 2015;28(6):302-9.
231. Gugliotta C, Deiana G, Dettori M, Sotgiu G, Azara A, Castiglia P. Prevalence study on health-care associated infections and on the use of antimicrobials carried out with the light protocol of the European Centre for Disease Prevention and Control. Ann Ig Med Prev Comu. 2020;32(4):357-67.
232. Ababneh MA, Jaber M, Rababa'H A, Alabweny E. Prevalence of antimicrobial use in a tertiary academic hospital: a venue for antimicrobial stewardship programs. Expert Rev Anti-Infe. 2021;19(8):1047-51.
233. Mijovic G, Cizmovic L, Vukovic MN, Stamatovic S, Lopicic M. Antibiotic consumption in hospitals and resistance rate of Klebsiella pneumoniae and Escherichia coli in Montenegro. Acta Clin Croat. 2020;59(3):469-79.
234. Wushouer H, Wang Z, Tian Y, Zhou Y, Zhu D, Vuillermin D, et al. The impact of physicians' knowledge on outpatient antibiotic use: evidence from China's county hospitals. Medicine. 2020;99(3):e18852.
235. Chui C, Cowling BJ, Lim WW, Hui C, Chan EW, Wong I, et al. Patterns of inpatient antibiotic use among public hospitals in Hong Kong from 2000 to 2015. Drug Safety. 2020;43(6):595-606.
236. Murni IK, Duke T, Kinney S, Daley AJ, Soenarto Y. Reducing hospital-acquired infections and improving the rational use of antibiotics in a developing country: an effectiveness study. Arch Dis Child. 2015;100(5):454-9.
237. Korinteli IG, Mchedlishvili I, Javakhadze M, Versporten A, Goossens H, Phagava H, et al. The global point prevalence survey (PPS) of antimicrobial use and antimicrobial resisrance among hospitalized children in Georgia. Georgian Med News. 2019;No 7-8(292-293):72-5.
238. Gibbons CL, Malcolm W, Sneddon J, Doherty C, Cairns S, Milne A, et al. Establishing a baseline for a national paediatric antimicrobial stewardship programme. J Antimicrob Chemoth. 2019;74(10):3104-10.

239. Okoro RN, Nmeka C, Erah PO. Antibiotics prescription pattern and determinants of utilization in the national health insurance scheme at a tertiary hospital in Nigeria. Afr Health Sci. 2019;19(3):2356-64.
240. Cole CP, Routledge P. An evaluation of rational prescribing in hospital outpatient practice in Sierra Leone and assessment of affordability of a prescription as an outcome. Pan Afr Med J. 2018;31(174):16729.
241. Arnoldo L, Smaniotto C, Celotto D, Brunelli L, Cocconi R, Tignonsini D, et al. Monitoring healthcare-associated infections and antimicrobial use at regional level through repeated point prevalence surveys: what can be learnt? J Hosp Infect. 2019;101(4):447-54.
242. Mao W, Huang Y, Chen W. An analysis on rational use and affordability of medicine after the implementation of National Essential Medicines Policy and Zero Mark-up Policy in Hangzhou, China. Plos One. 2019;14(3):e213638.
243. Nguyen S, Lefebure A, Lescure FX, Arnaud P, Rioux C. Evaluation of broad-spectrum beta-lactam prescriptions (except carbapenems) in a French teaching hospital. Med Maladies Infect. 2018;48(8):509-15.
244. Palms DL, Hicks LA, Bartoces M, Hersh AL, Zetts R, Hyun DY, et al. Comparison of antibiotic prescribing in retail clinics, urgent care centers, emergency departments, and traditional ambulatory care settings in the United States. Jama Intern Med. 2018;178(9):1267-9.
245. Naughton C, Hennessy Y, Mannion C, Philbin M. A comparison of antibiotic point prevalence survey data from four Irish regional/general hospitals. Irish J Med Sci. 2011;180(2):457-61.
246. Al-Azayzih A, Al-Azzam SI, Alzoubi KH, Shawaqfeh M, Masadeh MM. Evaluation of drug-prescribing based on the WHO prescribing indicators at outpatient clinics of five hospitals in Jordan: a cross-sectional study. Int J Clin Pharm Th. 2017;55(5):425-32.
247. Gerber JS, Newland JG, Coffin SE, Hall M, Thurm C, Prasad PA, et al. Variability in antibiotic use at children's hospitals. Pediatrics. 2010;126(6):1067-73.
248. Ider BE, Clements A, Adams J, Whitby M, Muugolog T. Prevalence of hospital-acquired infections and antibiotic use in two tertiary Mongolian hospitals. J Hosp Infect. 2010;75(3):214-9.
249. Arcavi L, Okasha D, Trepp S, Nehemya M, Kassis I, Haddad S, et al. Appropriate antibiotic prescribing pattern in hospitalized children. Curr Drug Saf. 2010;5(3):194-202.
250. Ahmed AM, Awad AI. Drug use practices at pediatric hospitals of Khartoum State, Sudan. Ann Pharmacother. 2010;44(12):1986-93.
251. Mohlala G, Peltzer K, Phaswana-Mafuya N, Ramlagan S. Drug prescription habits in public and private health facilities in 2 provinces in South Africa. E Mediterr Health J. 2010;16(3):324-8.
252. Evirgen O, Onlen Y, Ertan O. The intensity of antibiotic usage in the university hospital and the investigation of an inappropriate use of antibiotics. Bratisl Med J. 2011;112(10):595-8.

253. Etienne P, Roger PM, Brofferio P, Labate C, Blanc V, Tiger F, et al. Antimicrobial stewardship program and quality of antibiotic prescriptions. Med Maladies Infect. 2011;41(11):608-12.
254. Gwebu P, Meyer J, Schellack N, Matsebula-Myeni Z, Godman B. A web-based point prevalence survey of antimicrobial use and quality indicators at Raleigh Fitkin Memorial Hospital in Eswatini and the implications. Pharmacoepidem Dr S. 2022;50(6):214-21.
255. Gulmez SE, Guldan M, Ucar EA, Karakus MB, Sahin SM, Sisman U, et al. Antibacdus-pan: antibacterial utilization among adult patients at Koc University Hospital before and during COVID-19 pandemic within 12-months period: a hospital pharmacoepidemiology study. Pharmacoepidem Dr S. 2022;4(1):30-9.
256. Guezeloglu E, Karaci M. Antibiotic-associated adverse drug events in hospitalized children. J Pediatr Infect. 2022;16(3):E198-204.
257. Park SY, Moon SM, Kim B, Lee MJ, Park JY, Hwang S, et al. Appropriateness of antibiotic prescriptions during hospitalization and ambulatory care: a multicentre prevalence survey in Korea. J Glob Antimicrob Re. 2022;29:253-8.
258. Kitt E, Hayes M, Ballester L, Sewawa KB, Mulale U, Mazhani L, et al. Assessing antibiotic utilization among pediatric patients in Gaborone, Botswana. Sage Open Med. 2022;10:1-10.
259. Yang Q, Yuan F, Li L, Jin J, He J. Effects of monthly evaluations on the rates of irrational antimicrobial prescription in the outpatient and emergency departments at Ningbo No. 6 Hospital, Ningbo, China. Eur J Med Res. 2022;27(1):98-104.
260. Talaat M, Tolba S, Abdou E, Sarhan M, Gomaa M, Hutin YJ. Over-prescription and overuse of antimicrobials in the Eastern Mediterranean Region: the urgent need for Antimicrobial Stewardship Programs with Access, Watch, and Reserve adoption. Antibiotics-Basel. 2022;11(12):1773.
261. Sheikh S, Vishwas G, Aggarwal M, Bhattacharya S, Kumari P, Parashar L, et al. Antibiotic point prevalence survey at a tertiary healthcare hospital in India: identifying strategies to improve the antibiotic stewardship program immediately after a COVID-19 wave. Infection prevention in practice. 2022;4(4):100253.
262. Zhang J, Zhang W, Ma X, Tang L, Tian D, Wu K, et al. Antimicrobial prescribing for children in China: data from point prevalence surveys in 18 tertiary centres in China in 2016-2017. Bmj Open. 2022;12(9).
263. Shaikh Q, Sarfaraz S, Rahim A, Hussain A, Behram S, Kazi AS, et al. WHO point prevalence survey to describe the use of antimicrobials at a tertiary care center in Pakistan: a situation analysis for establishing an antimicrobial stewardship program. Antibiotics-Basel. 2022;11(11):1555.
264. Oghuvwu SO, Isah A. Prescription patterns and patient care practices in two tertiary hospitals in south-south Nigeria. West African journal of medicine. 2023;40(1):78-83.
265. Ranasinghe P, Liyanage CK, Meegoda J, Jayakody RL, Galappatthy P. National survey on World Health Organization/International Network of Rational Use of Drugs core drug use indicators in the outpatient setting and availability of medicines in a developing South Asian country. J Pharm Health Serv. 2022;13(2):158-65.
266. Akkawi ME, Taffour RM, AL-Shami AM. Evaluation of antibiotic prescribing pattern and appropriateness among hospitalized pediatric patients: findings from a

Malaysian teaching hospital. Infect Dis Rep. 2022;14(6):889-99.
267. Nunez-Nunez M, Perez-Galera S, Antonio Giron-Ortega J, Sandoval Fernandez-Del-Castillo S, Beltran-Garcia M, De Cueto M, et al. Predictors of inappropriate antimicrobial prescription: eight-year point prevalence surveys experience in a third level hospital in Spain. Front Pharmacol. 2022;13:1018158.
268. Omulo S, Oluka M, Achieng L, Osoro E, Kinuthia R, Guantai A, et al. Point-prevalence survey of antibiotic use at three public referral hospitals in Kenya. Plos One. 2022;17(6):e270048.
269. Gutiérrez-Urbón JM, Arenere-Mendoza M, Fernández-de-Gamarra-Martínez E, Fernández-Polo A, González-Suárez S, Nicolás-Picó J, et al. PAUSATE study: prevalence and appropriateness of the use of antimicrobials in Spanish hospitals. Farm Hosp. 2022;46(5):271-81.
270. Joshi R, Medhi B, Prakash A, Chandy S, Ranjalkar J, Bright HR, et al. Assessment of prescribing pattern of drugs and completeness of prescriptions as per the World Health Organization prescribing indicators in various Indian tertiary care centers: a multicentric study by rational use of medicines centers-Indian Council of Medical Research Network under National Virtual Centre Clinical Pharmacology activity. Indian J Pharmacol. 2022;54(5):321-8.
271. Nguyen HQ, Nguyen-Thi HY, Huynh PT, Le NDT, Nguyen NT, Hsia Y. Effectiveness of an enhanced antibiotic stewardship programme among paediatric patients in a tertiary hospital in Vietnam. J Hosp Infect. 2022;127:121-8.
272. Sharif M, Aslam S, Saleem Z. Point prevalence survey to estimate antimicrobial use in a tertiary care university hospital in Pakistan using WHO methodology: findings and implications. Infect Dis-Nor. 2022;54(9):698-701.
273. Versporten A, Zarb P, Caniaux I, Gros MF, Drapier N, Miller M, et al. Antimicrobial consumption and resistance in adult hospital inpatients in 53 countries: results of an internet-based global point prevalence survey. Lancet Glob Health. 2018;6(6):e619-29.
274. Saleem Z, Haseeb A, Godman B, Batool N, Altaf U, Ahsan U, et al. Point prevalence survey of antimicrobial use during the COVID-19 Pandemic among different hospitals in Pakistan: findings and implications. Antibiotics-Basel. 2022;12(1):70.
275. Limato R, Nelwan EJ, Mudia M, de Brabander J, Guterres H, Enty E, et al. A multicentre point prevalence survey of patterns and quality of antibiotic prescribing in Indonesian hospitals. Jac-Antimicrob Resis. 2021;3(2):b47.
276. Ishibashi N, Pauwels I, Tomori Y, Gu Y, Yamaguchi T, Handa T, et al. Point prevalence surveys of antimicrobial prescribing in a non-acute care hospital in Saitama Prefecture, Japan. Can J Infect Dis Med. 2022;2022:2497869.
277. Jamaluddin N, Periyasamy P, Lau CL, Ponnampalavanar S, Lai P, Ramli R, et al. Point prevalence survey of antimicrobial use in a Malaysian tertiary care university hospital. Antibiotics-Basel. 2021;10(5):531.
278. Ashour RH, Abdelkader EA, Hamdy O, Elmetwally M, Laimon W, Abd-Elaziz MA. The pattern of antimicrobial prescription at a tertiary health center in Egypt: a point survey and implications. Infect Drug Resist. 2022;15:6365-78.

279. Panditrao AM, Shafiq N, Chatterjee S, Pathak A, Trivedi N, Sadasivam B, et al. A multicentre point prevalence survey (PPS) of antimicrobial use amongst admitted patients in tertiary care centres in India. J Antimicrob Chemoth. 2021;76(4):1094-101.
280. Rashid MM, Akhtar Z, Chowdhury S, Islam MA, Parveen S, Ghosh PK, et al. Pattern of antibiotic use among hospitalized patients according to WHO Access, Watch, Reserve (AWaRe) classification: findings from a point prevalence survey in Bangladesh. Antibiotics-Basel. 2022;11(6).
281. Ankrah D, Owusu H, Aggor A, Osei A, Ampomah A, Harrison M, et al. Point prevalence survey of antimicrobial utilization in Ghana's premier hospital: implications for antimicrobial stewardship. Antibiotics-Basel. 2021;10(12):1528.
282. D'Arcy N, Ashiru-Oredope D, Olaoye O, Afriyie D, Akello Z, Ankrah D, et al. Antibiotic prescribing patterns in Ghana, Uganda, Zambia and Tanzania hospitals: results from the Global Point Prevalence Survey (G-PPS) on antimicrobial use and stewardship interventions implemented. Antibiotics-Basel. 2021;10(9):1122.
283. Kiggundu R, Wittenauer R, Waswa JP, Nakambale HN, Kitutu FE, Murungi M, et al. Point prevalence survey of antibiotic use across 13 hospitals in Uganda. Antibiotics-Basel. 2022;11(2):199.
284. Wang CN, Tong J, Yi B, Huttner BD, Cheng Y, Li S, et al. Antibiotic use among hospitalized children and neonates in China: results from quarterly point prevalence surveys in 2019. Front Pharmacol. 2021;12:601561.
285. Rocke T, El ON, Quiros RE, Hsieh J, Ramon-Pardo P. Reporting on antibiotic use patterns using the WHO Access, Watch, Reserve classification in the Caribbean. Rev Panam Salud Publ. 2022;46:e186.
286. Oo WT, Carr SD, Marchello CS, San MM, Oo AT, Oo KM, et al. Point-prevalence surveys of antimicrobial consumption and resistance at a paediatric and an adult tertiary referral hospital in Yangon, Myanmar. Infect Prev Pract. 2022;4(1):100197.
287. Spernovasilis N, Kritsotakis EI, Mathioudaki A, Vouidaski A, Markaki I, Psaroudaki D, et al. Antimicrobial prescribing before and after the implementation of a carbapenem-focused antimicrobial stewardship program in a Greek tertiary hospital during the COVID-19 Pandemic. Antibiotics-Basel. 2022;12(1):39.
288. Okoye BI, Udemba JC, Ndugba CA, Okonkwo JI, Obed EA. Evaluation of rational prescribing in a hospital paediatric outpatient clinic in Nigeria. Bmj Paediatr Open. 2022;6(1):e1585.
289. El-Dahiyat F, Salah D, Alomari M, Elrefae A, Jairoun AA. Antibiotic prescribing patterns for outpatient pediatrics at a private hospital in Abu Dhabi: a clinical audit study. Antibiotics-Basel. 2022;11(12):1676.
290. Mittal N, Mittal R, Goel N, Parmar A, Bahl A, Kaur S, et al. WHO-Point Prevalence Survey of antibiotic use among inpatients at a core national antimicrobial consumption network site in north India: findings and implications. Microbial drug resistance (Larchmont, N.Y.). 2023;29(1):1-9.
291. Sidamo T, Deboch A, Abdi M, Debebe F, Dayib K, Balcha Balla T. Assessment of polypharmacy, drug use patterns, and associated factors at the Edna Adan University Hospital, Hargeisa, Somaliland. J Trop Med-Us. 2022;2022:2858987.

292. Tadesse TY, Molla M, Yimer YS, Tarekegn BS, Kefale B. Evaluation of antibiotic prescribing patterns among inpatients using World Health Organization indicators: a cross-sectional study. Sage Open Med. 2022;10(2):1-10.
293. Sodhi B, Basu S. Antibiotic prescription audits among pediatric outpatients with acute ailments in a secondary care hospital during the COVID-19 omicron wave in northern India. Cureus J Med Science. 2022;14(11):e32017.
294. Amponsah OKO, Nagaraja SB, Ayisi-Boateng NK, Nair D, Muradyan K, Asense PS, et al. High levels of outpatient antibiotic prescription at a district hospital in Ghana: results of a cross sectional study. Int J Env Res Pub He. 2022;19(16):10286.
295. Skosana PP, Schellack N, Godman B, Kurdi A, Bennie M, Kruger D, et al. A national, multicentre, web-based point prevalence survey of antimicrobial use and quality indices among hospitalised paediatric patients across South Africa. J Glob Antimicrob Re. 2022;29:542-50.
296. Yimer YS, Addis GT, Alemu MA. Evaluation of prescription completeness, rational drug-use patterns using WHO prescribing, patient-care and facility indicators in Debre Tabor Comprehensive Specialized Hospital, Ethiopia: a cross-sectional study. Sage Open Med. 2022;10(4).
297. Deiana G, Arghittu A, Gentili D, Dettori M, Palmieri A, Masia MD, et al. Impact of the COVID-19 Pandemic on the prevalence of HAIs and the use of antibiotics in an Italian university hospital. Healthcare-Basel. 2022;10(9):1597.
298. Meenakshi R, Selvaraj N, Anandabaskar N, Dhamodharan A, Badrinath AK, Rajamohammad MA. Prescription audit of a teaching hospital in south India using World Health Organization core prescribing indicators - a cross-sectional study. Perspectives in clinical research. 2022;13(3):132-6.
299. Abu Farha R, Awwad O, Abdurazaq B, Abu Hammour K, Akour A. Evaluation of drug use pattern in adults' outpatient clinics in a tertiary teaching hospital using WHO core prescribing indicators. J Pharm Health Serv. 2022;13(4):357-63.
300. Alnajjar MS, Jawhar DS, Aburuz S, Saeed DA, Ibrahim AH. Point prevalence survey of antibiotic utilization in secondary care hospital in the United Arab Emirates. Pharm Pract-Granada. 2022;20(3):1-6.
301. Anugulruengkitt S, Charoenpong L, Kulthanmanusorn A, Thienthong V, Usayaporn S, Kaewkhankhaeng W, et al. Point prevalence survey of antibiotic use among hospitalized patients across 41 hospitals in Thailand. Jac-Antimicrob Resis. 2022;5(1):c140.
302. Parathoduvil AA, Sujatha MB, Venugopal S. Prescription pattern of antibiotics in admitted patients of a tertiary care government teaching hospital, Kerala, India. J Clin Diagn Res. 2022;16(5):C1-7.
303. Kalungia AC, Mukosha M, Mwila C, Banda D, Mwale M, Kagulura S, et al. Antibiotic use and stewardship indicators in the first- and second-level hospitals in Zambia: findings and implications for the future. Antibiotics-Basel. 2022;11(11):1626.
304. Catho G, Sauser J, Coray V, Da Silva S, Elzi L, Harbarth S, et al. Impact of interactive computerised decision support for hospital antibiotic use (COMPASS): an open-label, cluster-randomised trial in three Swiss hospitals. Lancet Infect Dis. 2022;22(10):1493-502.

305. Chansamouth V, Chommanam D, Roberts T, Keomany S, Paphasiri V, Phamisith C, et al. Evaluation of trends in hospital antimicrobial use in the Lao PDR using repeated point-prevalence surveys-evidence to improve treatment guideline use. Lancet Reg Health-W. 2022;27:100531.
306. Usluer G, Ozgunes I, Leblebicioglu H. A multicenter point-prevalence study: antimicrobial prescription frequencies in hospitalized patients in Turkey. Ann Clin Microb Anti. 2005;4(1):16.
307. Ufer M, Radosevic N, Vogt A, Palcevski G, Francetic I, Reinalter SC, et al. Antimicrobial drug use in hospitalised paediatric patients: a cross-national comparison between Germany and Croatia. Pharmacoepidem Dr S. 2005;14(10):735-9.
308. McGregor JC, Weekes E, Forrest GN, Standiford HC, Perencevich EN, Furuno JP, et al. Impact of a computerized clinical decision support system on reducing inappropriate antimicrobial use: a randomized controlled trial. J Am Med Inform Assn. 2006;13(4):378-84.
309. Avci IY, Kilic S, Acikel CH, Ucar M, Hasde M, Eyigun CP, et al. Outpatient prescription of oral antibiotics in a training hospital in Turkey: trends in the last decade. J Infection. 2006;52(1):9-14.
310. Likic R, Francetic I, Bilusic M, Erdeljic V, Makar-Ausperger K, Junacko C, et al. Antibiotic use optimization program in the largest Croatian university hospital--benefits of restrictions on unlimited antibiotic use. Coll Antropol. 2007;31(1):241-6.
311. Seaton RA, Nathwani D, Burton P, McLaughlin C, MacKenzie AR, Dundas S, et al. Point prevalence survey of antibiotic use in Scottish hospitals utilising the Glasgow Antimicrobial Audit Tool (GAAT). Int J Antimicrob Ag. 2007;29(6):693-9.
312. Lee MK, Chiu CS, Chow VC, Lam RK, Lai RW. Prevalence of hospital infection and antibiotic use at a university medical center in Hong Kong. J Hosp Infect. 2007;65(4):341-7. Medline:17275959 doi:10.1016/j.jhin.2006.12.013
313. Vlahovic-Palcevski V, Dumpis U, Mitt P, Gulbinovic J, Struwe J, Palcevski G, et al. Benchmarking antimicrobial drug use at university hospitals in five European countries. Clin Microbiol Infec. 2007;13(3):277-83.
314. Al-Niemat SI, Bloukh DT, Al-Harasis MD, Al-Fanek AF, Salah RK. Drug use evaluation of antibiotics prescribed in a Jordanian hospital outpatient and emergency clinics using WHO prescribing indicators. Saudi Med J. 2008;29(5):743-8.
315. Akande TM, Ologe MO. Prescription pattern at a secondary health care facility in Ilorin, Nigeria. Usmanu Danfodiyo University Teaching Hospital. 2007;6(4):186-9.
316. Oshikoya KA, Ojo OI. Medication errors in paediatric outpatient prescriptions of a teaching hospital in Nigeria. Nig Q J Hosp Med. 2007;17(2):74-8.
317. Danchaivijitr S, Judaeng T, Sripalakij S, Naksawas K, Plipat T. Prevalence of nosocomial infection in Thailand 2006. J Med Assoc Thai. 2007;90(8):1524-9.
318. Hajdu A, Samodova OV, Carlsson TR, Voinova LV, Nazarenko SJ, Tjurikov AV, et al. A point prevalence survey of hospital-acquired infections and antimicrobial use in a paediatric hospital in north-western Russia. J Hosp Infect. 2007;66(4):378-84.
319. Tunger O, Karakaya Y, Cetin CB, Dinc G, Borand H. Rational antibiotic use. J Infect Dev Countr. 2009;3(2):88-93.

320. Dimina E, Kula M, Caune U, Vigante D, Liepins M, Zeidaka L, et al. Repeated prevalence studies on antibiotic use in Latvia, 2003-2007. Eurosurveillance. 2009;14(33):19307.
321. de With K, Bestehorn H, Steib-Bauert M, Kern WV. Comparison of defined versus recommended versus prescribed daily doses for measuring hospital antibiotic consumption. Infection. 2009;37(4):349-52.
322. Ciofi DAM, Raponi M, Tozzi AE, Ciliento G, Ceradini J, Langiano T. Point prevalence study of antibiotic use in a paediatric hospital in Italy. Eurosurveillance. 2008;13(41):19003.
323. Hariharan S, Pillai G, McIntosh D, Bhanji Z, Culmer L, Harper-McIntosh K. Prescribing patterns and utilization of antimicrobial drugs in a tertiary care teaching hospital of a Caribbean developing country. Fund Clin Pharmacol. 2009;23(5):609-15.
324. Dimri S, Tiwari P, Basu S, Parmar VR. Drug use pattern in children at a teaching hospital. Indian Pediatr. 2009;46(2):165-7.
325. Newman MJ. Nosocomial and community acquired infections in Korle Bu Teaching Hospital, Accra. West Afr J Med. 2009;28(5):300-3.
326. Ghimire S, Nepal S, Bhandari S, Nepal P, Palaian S. A prospective surveillance of drug prescribing and dispensing in a teaching hospital in western Nepal. J Pak Med Assoc. 2009;59(10):726-31.
327. Calligaris L, Panzera A, Arnoldo L, Londero C, Quattrin R, Troncon MG, et al. Errors and omissions in hospital prescriptions: a survey of prescription writing in a hospital. BMC Clin Pharmacol. 2009;9(1):9.
328. Ansari F, Erntell M, Goossens H, Davey P. The European Surveillance of Antimicrobial Consumption (ESAC) point-prevalence survey of antibacterial use in 20 European hospitals in 2006. Clin Infect Dis. 2009;49(10):1496-504.
329. Rivero M, Padilla B, Garcia-Lechuz J, Cruz MM, Rodriguez-Creixems M, Bouza E. Use and misuse of antimicrobial agents in a general hospital in the AIDS era. J Hosp Infect. 2000;46(3):230-5.
330. Erbay A, Colpan A, Bodur H, Cevik MA, Samore MH, Ergönül O. Evaluation of antibiotic use in a hospital with an antibiotic restriction policy. Int J Antimicrob Ag. 2003;21(4):308-12.
331. Potocki M, Goette J, Szucs TD, Nadal D. Prospective survey of antibiotic utilization in pediatric hospitalized patients to identify targets for improvement of prescription. Infection. 2003;31(6):398-403.
332. Mora Y, Avila-Aguero ML, Umana MA, Jimenez AL, Paris MM, Faingezicht I. Epidemiological observations of the judicious use of antibiotics in a pediatric teaching hospital. Int J Infect Dis. 2002;6(1):74-7.
333. Raveh D, Levy Y, Schlesinger Y, Greenberg A, Rudensky B, Yinnon AM. Longitudinal surveillance of antibiotic use in the hospital. Qjm-Int J Med. 2001;94(3):141-52.

334. Tunger O, Dinc G, Ozbakkaloglu B, Atman UC, Algun U. Evaluation of rational antibiotic use. Int J Antimicrob Ag. 2000;15(2):131-5.
335. Berild D, Ringertz SH, Lelek M. Appropriate antibiotic use according to diagnoses and bacteriological findings: report of 12 point-prevalence studies on antibiotic use in a university hospital. Scand J Infect Dis. 2002;34(1):56-60.
336. McDonald LC, Yu HT, Yin HC, Hsiung CA, Hung CC, Ho M. Correlates of antibiotic use in Taiwan hospitals. Infect Cont Hosp Ep. 2001;22(9):565-71.
337. Berild D, Ringertz SH, Aabyholm G, Lelek M, Fosse B. Impact of an antibiotic policy on antibiotic use in a paediatric department. Individual based follow-up shows that antibiotics were chosen according to diagnoses and bacterial findings. Int J Antimicrob Ag. 2002;20(5):333-8.
338. Apisarnthanarak A, Danchaivijitr S, Bailey TC, Fraser VJ. Inappropriate antibiotic use in a tertiary care center in Thailand: an incidence study and review of experience in Thailand. Infect Cont Hosp Ep. 2006;27(4):416-20.
339. Karande S, Sankhe P, Kulkarni M. Patterns of prescription and drug dispensing. Indian J Pediatr. 2005;72(2):117-21.
340. Fonseca LG, de Oliveira Conterno L. Audit of antibiotic use in a Brazilian university hospital. The Brazilian journal of infectious diseases. 2004;8(4):272-80.
341. Starakis I, Marangos M, Gikas A, Pediaditis I, Bassaris H. Repeated point prevalence survey of nosocomial infections in a Greek university hospital. J Chemotherapy. 2002;14(3):272-8.
342. Chukwuani CM, Onifade M, Sumonu K. Survey of drug use practices and antibiotic prescribing pattern at a general hospital in Nigeria. Pharm World Sci. 2002;24(5):188-95.
343. Gikas A, Pediaditis J, Papadakis JA, Starakis J, Levidiotou S, Nikolaides P, et al. Prevalence study of hospital-acquired infections in 14 Greek hospitals: planning from the local to the national surveillance level. J Hosp Infect. 2002;50(4):269-75.
344. Kanerva M, Ollgren J, Lyytikainen O. Antimicrobial use in Finnish acute care hospitals: data from national prevalence survey, 2005. J Antimicrob Chemoth. 2007;60(2):440-4.
345. Willemsen I, Groenhuijzen A, Bogaers D, Stuurman A, van Keulen P, Kluytmans J. Appropriateness of antimicrobial therapy measured by repeated prevalence surveys. Antimicrob Agents Ch. 2007;51(3):864-7.
346. Shankar PR, Upadhyay DK, Subish P, Dubey AK, Mishra P. Prescribing patterns among paediatric inpatients in a teaching hospital in western Nepal. Singap Med J. 2006;47(4):261-5.
347. Desai NM, Sadlowski JL, Mistry RD. Antibiotic prescribing for viral respiratory infections in the pediatric emergency department and urgent care. Pediatr Infect Dis J. 2020;39(5):406-10.
348. Anteneh DA, Kifle ZD, Mersha GB, Ayele TT. Appropriateness of antibiotics use and associated factors in hospitalized patients at University of Gondar Specialized Hospital, Amhara, Ethiopia: prospective follow-up study. Inquiry. 2021;58(2):1448312824.

349. Cotter JM, Florin TA, Moss A, Suresh K, Ramgopal S, Navanandan N, et al. Factors associated with antibiotic use for children hospitalized with pneumonia. Pediatrics. 2022;150(2):e2021054677.
350. Dilworth TJ, Hietpas K, Kram J, Baumgardner D. Impact of geodemographic factors on antibiotic prescribing for acute, uncomplicated bronchitis or upper respiratory tract infection. J Am Board Fam Med. 2022;35(4):733-41.
351. Kroening-Roche JC, Soroudi A, Castillo EM, Vilke GM. Antibiotic and bronchodilator prescribing for acute bronchitis in the emergency department. J Emerg Med. 2012;43(2):221-7.
352. Linder JA, Singer DE. Desire for antibiotics and antibiotic prescribing for adults with upper respiratory tract infections. J Gen Intern Med. 2003;18(10):795-801.
353. Kornblith AE, Fahimi J, Kanzaria HK, Wang RC. Predictors for under-prescribing antibiotics in children with respiratory infections requiring antibiotics. Am J Emerg Med. 2018;36(2):218-25.
354. Kozyrskyj AL, Dahl ME, Chateau DG, Mazowita GB, Klassen TP, Law BJ. Evidence-based prescribing of antibiotics for children: role of socioeconomic status and physician characteristics. Can Med Assoc J. 2004;171(2):139-45.
355. Curt AM, Lipsett SC, Neuman MI. Antibiotic prescribing and parent satisfaction for children with respiratory illness. Clin Pediatr. 2020;59(6):618-21.
356. Kumar R, Indira K, Rizvi A, Rizvi T, Jeyaseelan L. Antibiotic prescribing practices in primary and secondary health care facilities in Uttar Pradesh, India. J Clin Pharm Ther. 2008;33(6):625-34.
357. Cantrell R, Young AF, Martin BC. Antibiotic prescribing in ambulatory care settings for adults with colds, upper respiratory tract infections, and bronchitis. Clin Ther. 2002;24(1):170-82.
358. Arnold SR, To T, McIsaac WJ, Wang EE. Antibiotic prescribing for upper respiratory tract infection: the importance of diagnostic uncertainty. J Pediatr-Us. 2005;146(2):222-6.
359. Cadieux G, Abrahamowicz M, Dauphinee D, Tamblyn R. Are physicians with better clinical skills on licensing examinations less likely to prescribe antibiotics for viral respiratory infections in ambulatory care settings? Med Care. 2011;49(2):156-65.
360. Barnett ML, Linder JA. Antibiotic prescribing for adults with acute bronchitis in the United States, 1996-2010. Jama-J Am Med Assoc. 2014;311(19):2020-2.
361. Bergmark RW, Sedaghat AR. Antibiotic prescription for acute rhinosinusitis: emergency departments versus primary care providers. Laryngoscope. 2016;126(11):2439-44.
362. Depew RE, Gonzales G. Differences in US antibiotic prescription use by facility and patient characteristics: evidence from the National Ambulatory Medical Care Survey. Fam Pract. 2020;37(2):180-6.
363. Coco AS, Horst MA, Gambler AS. Trends in broad-spectrum antibiotic prescribing for children with acute otitis media in the United States, 1998-2004. Bmc Pediatr.

2009;9:41.
364. Copp HL, Shapiro DJ, Hersh AL. National ambulatory antibiotic prescribing patterns for pediatric urinary tract infection, 1998-2007. Pediatrics. 2011;127(6):1027-33.
365. Ababneh MA, Al-Azzam SI, Ababneh R, Rababa'H AM, Demour SA. Antibiotic prescribing for acute respiratory infections in children in Jordan. Int Health. 2017;9(2):124-30.
366. Chang LY, Lai CC, Chen CJ, Cho CY, Luo YC, Jeng MJ, et al. Recent trends in prescribing antibiotics for acute tonsillitis in pediatric ambulatory care in Taiwan, 2000-2009: a nationwide population-based study. J Microbiol Immunol. 2017;50(4):500-6.
367. Ardoino I, Mannucci PM, Nobili A, Franchi C. Antibiotic use and associated factors in a large sample of hospitalised older people. J Glob Antimicrob Re. 2019;19:167-72.
368. Covino M, Buonsenso D, Gatto A, Morello R, Curatole A, Simeoni B, et al. Determinants of antibiotic prescriptions in a large cohort of children discharged from a pediatric emergency department. Eur J Pediatr. 2022;181(5):2017-30.
369. Kourlaba G, Gkrania-Klotsas E, Kourkouni E, Mavrogeorgos G, Zaoutis TE. Antibiotic prescribing and expenditures in outpatient adults in Greece, 2010 to 2013: evidence from real-world practice. Eurosurveillance. 2016;21(26):30266.
370. Lin YC, Lin HC, Lin HC. Doctor characteristics and prescribing antibiotics for urinary tract infections: the experience of an Asian country. J Eval Clin Pract. 2010;16(6):1221-6.
371. Kronman MP, Hersh AL, Feng R, Huang YS, Lee GE, Shah SS. Ambulatory visit rates and antibiotic prescribing for children with pneumonia, 1994-2007. Pediatrics. 2011;127(3):411-8.
372. Forster CS, Almaazi A, Hamdy R, Harik N. Predictors of empiric antibiotic use in the emergency department in children without urinary tract infections. Pediatr Emerg Care. 2022;38(5):e1251-6.
373. Goodman KE, Baghdadi JD, Magder LS, Heil EL, Sutherland M, Dillon R, et al. Patterns, predictors, and intercenter variability in empiric Gram-Negative antibiotic use across 928 United States hospitals. Clin Infect Dis. 2023;76(3):e1224-35.
374. Hadi U, Duerink DO, Lestari ES, Nagelkerke NJ, Keuter M, Huis IVD, et al. Audit of antibiotic prescribing in two governmental teaching hospitals in Indonesia. Clin Microbiol Infec. 2008;14(7):698-707.
375. Hersh AL, Shapiro DJ, Pavia AT, Shah SS. Antibiotic prescribing in ambulatory pediatrics in the United States. Pediatrics. 2011;128(6):1053-61.
376. Kawanami GH, Fortaleza CM. Factors predictive of inappropriateness in requests for parenteral antimicrobials for therapeutic purposes: a study in a small teaching hospital in Brazil. Scand J Infect Dis. 2011;43(6-7):528-35.
377. Jewell MJ, Leyenaar J, Shieh MS, Pekow PS, Stefan M, Lindenauer PK. Unnecessary antibiotic prescribing in children hospitalised for asthma exacerbation: a

retrospective national cohort study. Bmj Qual Saf. 2021;30(4):292-9.
378. Hashimoto H, Matsui H, Sasabuchi Y, Yasunaga H, Kotani K, Nagai R, et al. Antibiotic prescription among outpatients in a prefecture of Japan, 2012-2013: a retrospective claims database study. Bmj Open. 2019;9(4):e26251.
379. Hadi U, Duerink DO, Lestari ES, Nagelkerke NJ, Werter S, Keuter M, et al. Survey of antibiotic use of individuals visiting public healthcare facilities in Indonesia. Int J Infect Dis. 2008;12(6):622-9.
380. Stone S, Gonzales R, Maselli J, Lowenstein SR. Antibiotic prescribing for patients with colds, upper respiratory tract infections, and bronchitis: a national study of hospital-based emergency departments. Ann Emerg Med. 2000;36(4):320-7.
381. Vanderweil SG, Tsai CL, Pelletier AJ, Espinola JA, Sullivan AF, Blumenthal D, et al. Inappropriate use of antibiotics for acute asthma in United States emergency departments. Acad Emerg Med. 2008;15(8):736-43.
382. Shapiro DJ, Hicks LA, Pavia AT, Hersh AL. Antibiotic prescribing for adults in ambulatory care in the USA, 2007-09. J Antimicrob Chemoth. 2014;69(1):234-40.
383. Sencan I, Cag Y, Karabay O, Kurtaran B, Guclu E, Ogutlu A, et al. Antibiotic use and influencing factors among hospitalized patients with COVID-19: a multicenter point-prevalence study from Turkey. Balk Med J. 2022;39(3):209-17.
384. Shin SM, Shin JY, Kim MH, Lee SH, Choi S, Park BJ. Prevalence of antibiotic use for pediatric acute upper respiratory tract infections in Korea. J Korean Med Sci. 2015;30(5):617-24.
385. Zhang Z, Hu Y, Zou G, Lin M, Zeng J, Deng S, et al. Antibiotic prescribing for upper respiratory infections among children in rural China: a cross-sectional study of outpatient prescriptions. Global Health Action. 2017;10(1):1287334.
386. Teixeira RA, Ferreira M, Pineiro-Lamas M, Falcao A, Figueiras A, Herdeiro MT. Determinants of physician antibiotic prescribing behavior: a 3 year cohort study in Portugal. Curr Med Res Opin. 2016;32(5):949-57.
387. Velasco E, Espelage W, Faber M, Noll I, Ziegelmann A, Krause G, et al. A national cross-sectional study on socio-behavioural factors that influence physicians' decisions to begin antimicrobial therapy. Infection. 2011;39(4):289-97.
388. Havers FP, Hicks LA, Chung JR, Gaglani M, Murthy K, Zimmerman RK, et al. Outpatient antibiotic prescribing for acute respiratory infections during influenza seasons. Jama Netw Open. 2018;1(2):e180243.
389. Salzo A, Ripabelli G, Sammarco ML, Mariano A, Niro C, Tamburro M. Healthcare-associated infections and antibiotics consumption: a comparison of point prevalence studies and intervention strategies. Hosp Top. 2021;99(3):140-50.
390. Opoku MM, Bonful HA, Koram KA. Antibiotic prescription for febrile outpatients: a health facility-based secondary data analysis for the Greater Accra region of Ghana. Bmc Health Serv Res. 2020;20(1):978.

391. Morley VJ, Firgens E, Vanderbilt RR, Zhou Y, Zook M, Read AF, et al. Factors associated with antibiotic prescribing for acute bronchitis at a university health center. Bmc Infect Dis. 2020;20(1):177.

392. McKay R, Patrick DM, McGrail K, Law MR. Antibiotic prescribing for pediatric respiratory infections: what explains a large variation among physicians? Can Fam Physician. 2019;65(6):e278-91.

393. Manne M, Deshpande A, Hu B, Patel A, Taksler GB, Misra-Hebert AD, et al. Provider variation in antibiotic prescribing and outcomes of respiratory tract infections. South Med J. 2018;111(4):235-42.

394. Paul IM, Maselli JH, Hersh AL, Boushey HA, Nielson DW, Cabana MD. Antibiotic prescribing during pediatric ambulatory care visits for asthma. Pediatrics. 2011;127(6):1014-21.

395. Moro ML, Marchi M, Gagliotti C, Di Mario S, Resi D. Why do paediatricians prescribe antibiotics? Results of an Italian regional project. Bmc Pediatr. 2009;9:69.

396. Osatakul S, Puetpaiboon A. Appropriate use of empirical antibiotics in acute diarrhoea: a cross-sectional survey in southern Thailand. Ann Trop Paediatr. 2007;27(2):115-22.

397. Rutschmann OT, Domino ME. Antibiotics for upper respiratory tract infections in ambulatory practice in the United States, 1997-1999: does physician specialty matter? J Am Board Fam Pract. 2004;17(3):196-200.

398. Nadeem AM, Muyot MM, Begum S, Smith P, Little C, Windemuller FJ. Antibiotic prescription pattern for viral respiratory illness in emergency room and ambulatory care settings. Clin Pediatr. 2010;49(6):542-7.

399. Aspinall SL, Good CB, Metlay JP, Mor MK, Fine MJ. Antibiotic prescribing for presumed nonbacterial acute respiratory tract infections. Am J Emerg Med. 2009;27(5):544-51.

400. Ahmad A, Nor J, Abdullah AA, Tuan KT, Yazid MB. Patient factors in inappropriate antibiotic prescribing for upper respiratory tract infection in the emergency department. Malays J Med Sci. 2021;28(2):72-83.

401. Sawaya RD, El ZT, Mrad S, Abdul MC, Shaya S, Makki M, et al. Comparing febrile children presenting on and off antibiotics to the emergency department: a retrospective cohort study. Bmc Pediatr. 2020;20(1):117.

402. Steinberg MB, Akincigil A, Kim EJ, Shallis R, Delnevo CD. Tobacco smoking as a risk factor for increased antibiotic prescription. Am J Prev Med. 2016;50(6):692-8.

403. Zhao H, Bian J, Han X, Zhang M, Zhan S. Outpatient antibiotic use associated with acute upper respiratory infections in China: a nationwide cross-sectional study. Int J Antimicrob Ag. 2020;56(6):106193.

Supplementary Table 6. Results of Quality evaluation

Table S6. Quality evaluation of included studies

Study	Q1	Q2	Q3	Q4	Q5	Q6	Q7	Q8	Q9	Total
Fentie AM et al., 2022	1	1	0	1	1	1	0	1	1	7
Levy HG et al., 2022	1	1	0	1	1	1	1	1	1	8
Moulin E et al., 2022	1	0	0	1	1	1	0	1	1	6
Magill SS et al., 2021	1	1	1	1	1	0	1	1	1	8
Mustafa ZU et al., 2022	1	1	0	1	1	1	1	1	1	8
Muro FJ et al., 2022	1	1	0	1	1	1	0	1	1	7
Yi S et al., 2022	1	1	0	1	1	1	0	1	1	7
Xavier SP et al., 2022	1	0	1	1	1	1	0	1	1	7
Haseeb A et al., 2021	1	1	0	1	1	1	1	1	1	8
Ogunleye OO et al., 2022	1	0	0	1	1	1	0	1	1	6
Zhao H et al., 2021	1	1	0	1	1	1	1	1	1	8
Da Silva RMR et al., 2021	0	1	1	1	1	1	0	1	1	7
Boone K et al., 2021	1	0	0	1	1	1	1	1	1	7
Obura B et al., 2021	1	0	0	1	1	1	0	1	1	6
Kruger D et al., 2021	1	0	0	1	1	1	1	1	1	7
Kurdi A et al., 2021	1	0	0	1	1	1	1	1	1	7
Skosana PP et al., 2021	1	1	1	1	1	1	1	1	1	9
Arif S et al., 2021	1	1	0	1	1	0	0	1	1	6
Suljagic V et al., 2021	1	1	0	1	1	1	0	1	1	7
German GJ et al., 2021	1	1	0	1	1	1	0	1	1	7
Almansoori N et al., 2021	1	1	0	1	1	1	0	1	1	7
Blackburn J et al., 2021	1	1	0	1	1	1	1	1	1	8
Pauwels I et al., 2021	0	1	0	1	1	1	1	1	1	7
Oguz E et al., 2021	1	1	0	0	1	1	0	1	1	6
Tassew SG et al., 2021	0	1	1	1	1	1	0	1	1	7
Yehualaw A et al., 2021	1	1	1	1	1	1	0	1	0	7
Atal S et al., 2021	1	1	0	1	1	1	0	1	0	6
Shrestha JTM et al., 2021	1	0	0	1	1	1	0	1	1	6
Wendie TF et al., 2021	0	1	0	1	1	1	0	1	1	6
de Guzman Betito G et al., 2021	0	1	0	1	1	1	0	1	1	6
Magill SS et al., 2012	1	1	0	1	1	1	0	1	1	7
Chandelkar U et al., 2014	0	0	0	0	1	1	0	1	1	4
Momanyi L et al., 2019	1	0	0	1	1	1	1	1	0	6
Mama M et al., 2020	1	0	0	1	1	1	0	1	1	6
Rachina S et al., 2020	0	1	0	1	1	1	1	1	1	7
Barchitta M et al., 2020	0	1	0	1	1	1	1	1	0	6
Afriyie DK et al., 2020	0	0	0	0	1	1	1	1	0	4

Study	Q1	Q2	Q3	Q4	Q5	Q6	Q7	Q8	Q9	Total
Liang JJ et al., 2022	0	1	0	1	1	1	0	1	1	6
Soltani J et al., 2018	1	0	0	1	1	1	0	1	1	6
Holen O et al., 2017	0	1	0	0	1	1	0	1	1	5
Ahoyo TA et al., 2014	0	1	0	1	1	1	0	1	1	6
Charani E et al., 2019	1	1	0	1	1	1	0	1	1	7
Ughasoro MD et al., 2019	1	0	0	1	1	1	0	1	1	6
Ofori-Adjei YA et al., 2019	1	1	0	0	1	1	0	1	0	5
Gürtler N et al., 2019	1	1	1	1	1	1	0	1	1	8
Umeokonkwo CD et al., 2019	0	0	1	0	1	1	0	1	1	5
Hufnagel M et al., 2019	0	1	1	1	1	1	1	1	1	8
Zhang Y et al., 2019	0	1	1	1	1	0	0	1	1	6
Ciofi Degli Atti ML et al., 2019	1	1	0	1	1	1	1	1	1	8
Sheng T et al., 2019	1	1	0	1	1	1	0	1	1	7
Tersigni C et al., 2019	0	1	0	0	1	1	0	1	1	5
Saleem Z et al., 2019	1	1	1	1	1	1	1	1	1	9
Thaulow CM et al., 2019	1	1	0	1	1	1	0	1	1	7
Huerta-Gutiérrez R et al., 2019	1	1	0	1	1	1	0	1	1	7
Labi AK et al., 2019	1	1	1	1	1	1	0	1	1	8
Ripabelli G et al., 2019	1	0	0	1	1	1	0	1	1	6
Luthander J et al., 2019	1	1	0	1	1	1	1	1	1	8
Dlamini NN et al., 2019	1	0	0	1	1	1	1	1	1	7
Li H et al., 2019	1	1	1	1	1	1	1	1	0	8
Zingg W et al., 2019	1	1	0	1	1	1	1	1	1	8
Singh SK et al., 2019	0	1	0	0	1	1	1	1	1	6
Dyer AP et al., 2019	1	1	0	1	1	1	1	1	1	8
Peralta N et al., 2019	1	1	0	1	1	1	1	1	1	8
Al Matar M et al., 2019	1	1	0	1	1	1	0	1	1	7
Komagamine J et al., 2019	1	0	0	1	1	1	0	1	1	6
Yimenu DK et al., 2019	1	1	1	1	1	1	0	1	0	7
EmyInumaru F et al., 2019	1	1	0	1	1	1	0	1	1	7
Komagamine J et al., 2019	1	1	0	1	1	1	1	1	1	8
Lakoh S et al., 2020	1	1	0	1	1	1	1	1	1	8
Porto APM et al., 2020	1	1	0	1	1	1	1	1	1	8
Resurreccion-Delgado C et al., 2020	1	0	0	1	1	1	0	1	1	6
Fowotade A et al., 2020	1	0	0	1	1	1	0	1	1	6
Tribble AC et al., 2020	1	1	0	1	1	1	0	1	1	7
Hafeez M et al., 2020	1	1	0	1	1	1	0	1	1	7
Komagamine J et al., 2020	1	1	0	1	1	1	0	1	1	7
Masich AM et al., 2020	1	0	0	1	1	1	0	1	1	6
Frenette C et al., 2020	1	1	0	1	1	1	0	1	1	7
Roberts AA et al., 2020	1	0	0	0	1	1	0	1	1	5
Tham DWJ et al., 2020	1	0	0	1	1	1	0	1	1	6

Study	Q1	Q2	Q3	Q4	Q5	Q6	Q7	Q8	Q9	Total
Maina M et al., 2020	0	1	0	1	1	1	1	1	1	7
Griffith HG et al., 2020	1	1	0	1	1	1	0	1	1	7
Chautrakarn S et al., 2020	0	1	0	1	1	1	0	1	1	6
Khan Z et al., 2020	0	1	1	0	1	0	1	1	1	6
Yilma Z et al., 2020	0	1	1	0	1	1	0	1	1	6
Yilma Z et al., 2020	0	0	1	0	1	1	0	1	1	5
Nepal A et al., 2020	1	1	0	1	1	1	0	1	1	7
Frost HM et al., 2020	1	1	0	1	1	1	0	1	1	7
Miao R et al., 2020	0	1	0	1	1	1	1	1	1	7
Bimba HV et al., 2020	1	1	0	1	1	1	0	1	1	7
Seni J et al., 2020	1	1	0	1	1	1	1	1	1	8
Karabay O et al., 2020	0	1	1	1	1	1	0	1	1	7
Abubakar U et al., 2020	1	0	0	1	1	1	1	1	1	7
Horumpende PG et al., 2020	1	0	0	1	1	1	0	1	1	6
Vandael E et al., 2020	1	1	1	1	1	1	1	1	1	9
Mohammed ZA et al., 2020	1	0	1	1	1	1	0	1	0	6
Jaggi P et al., 2020	0	1	0	1	1	1	0	1	1	6
Thamlikitkul V et al., 2020	1	1	1	1	1	1	1	1	1	9
Al-Tawfiq JA et al., 2020	0	0	0	1	1	1	0	1	1	5
Antonioli P et al., 2020	1	1	0	1	1	1	1	1	1	8
Loftus MJ et al., 2020	1	0	0	1	1	1	0	1	1	6
Gardiner SJ et al., 2020	1	1	0	1	1	1	0	1	1	7
Xu JJ et al., 2020	1	1	1	1	1	1	0	1	1	8
Olaru ID et al., 2020	1	0	0	1	1	1	0	1	1	6
Wang CN et al., 2020	0	1	0	1	1	1	1	1	1	7
Zhang M et al., 2020	0	1	0	1	1	1	0	1	1	6
Cusini A et al., 2010	1	1	0	1	1	1	1	1	1	8
Black E et al., 2018	1	1	0	1	1	1	1	1	1	8
Oduyebo OO et al., 2017	0	1	0	1	1	0	0	1	1	5
Monteiro LGS et al., 2017	1	0	0	0	1	1	0	1	1	5
Nair V et al., 2015	1	1	0	1	1	1	0	1	1	7
Singh S et al., 2014	1	0	0	1	1	0	0	1	1	5
Denny KJ et al., 2019	1	1	0	1	1	1	0	1	1	7
Sikkens JJ et al., 2018	1	1	0	1	1	1	0	1	1	7
Croche Santander B et al., 2018	1	1	1	1	1	0	0	1	1	7
Labi AK et al., 2018	1	1	0	1	1	1	1	1	1	8
Olivier C et al., 2018	1	0	0	1	1	1	0	1	1	6
Labi AK et al., 2018	1	1	1	1	1	1	1	1	1	9
Zhang JS et al., 2018	0	1	0	1	1	1	0	1	1	6
Shrestha B et al., 2018	0	1	1	1	1	1	0	1	1	7
Mengistu G et al., 2020	0	1	1	1	1	1	0	1	1	7
Plachouras D et al., 2018	1	1	1	1	1	1	1	1	1	9

Study	Q1	Q2	Q3	Q4	Q5	Q6	Q7	Q8	Q9	Total
Al-Taani GM et al., 2018	1	1	0	1	1	0	1	1	1	7
Gutema G et al., 2018	1	1	1	1	1	1	1	1	1	9
Sticchi C et al., 2018	1	1	0	1	1	1	1	1	1	8
Elhajji FD et al., 2018	1	0	1	1	1	1	1	1	1	8
Koopmans LR et al., 2018	1	1	0	1	1	1	0	1	1	7
Okoth C et al., 2018	1	0	1	1	1	1	0	1	1	7
Metsini A et al., 2018	1	1	0	1	1	0	0	1	1	6
Morioka H et al., 2018	1	1	0	1	1	1	0	1	1	7
Amaha ND et al., 2018	0	0	1	1	1	1	0	1	1	6
Sviestina I et al., 2018	1	1	0	1	1	1	1	1	1	8
Yan K et al., 2018	0	1	1	1	1	1	1	1	0	7
Umar LW et al., 2018	1	1	0	1	1	1	0	1	1	7
Chaw PS et al., 2018	1	1	0	1	1	1	1	1	1	8
Hussain S et al., 2018	0	1	0	1	1	1	0	1	1	6
Cairns S et al., 2018	1	1	1	1	1	1	1	1	1	9
Wang S et al., 2018	0	1	0	0	1	0	0	1	1	4
Wang J et al., 2017	1	1	1	1	1	1	0	1	1	8
Chen Y et al., 2017	1	1	1	1	1	1	0	1	1	8
Ioannou P et al., 2022	1	1	0	1	1	1	0	1	1	7
Zeng L et al., 2017	1	0	0	1	1	1	1	1	1	7
Kebede HK et al., 2017	1	1	1	1	1	1	0	1	1	8
Goycochea-Valdivia WA et al., 2017	1	0	0	1	1	1	0	1	1	6
Sisay M et al., 2017	0	1	1	1	1	1	0	1	1	7
Cai Y et al., 2017	1	1	0	1	1	1	1	1	1	8
Guclu E et al., 2017	1	1	0	1	1	1	0	1	1	7
Liao XP et al., 2017	1	1	0	1	1	0	0	1	0	5
Fleming-Dutra KE et al., 2016	0	1	1	1	1	1	0	1	0	6
Lee ML et al., 2016	1	1	1	1	1	1	0	1	1	8
Versporten A et al., 2016	1	1	0	1	1	1	1	1	1	8
Morioka H et al., 2016	1	1	0	1	1	1	0	1	1	7
Fahimzad A et al., 2016	1	1	1	0	1	0	1	1	1	7
Gharbi M et al., 2016	1	1	0	1	1	1	1	1	1	8
Ren N et al., 2016	0	1	0	1	1	1	0	1	0	5
De Luca M et al., 2016	1	1	0	1	1	1	0	1	1	7
Stefkovicova M et al., 2016	1	1	1	1	1	1	1	1	1	9
Kiguba R et al., 2016	1	1	0	1	1	1	1	1	1	8
Bilal AI et al., 2016	0	1	1	1	1	1	0	1	1	7
Knezevic B et al., 2016	1	1	0	0	1	1	0	1	1	6
Segagni Lusignani L et al., 2016	1	1	0	1	1	1	1	1	1	8
Antonioli P et al., 2016	1	1	0	1	1	0	1	1	1	7
Vercheval C et al., 2016	1	1	0	1	1	1	1	1	1	8
Marani A et al., 2016	1	1	0	1	1	1	0	1	1	7

Study	Q1	Q2	Q3	Q4	Q5	Q6	Q7	Q8	Q9	Total
Zhang YW et al., 2016	1	1	0	1	1	1	0	1	1	7
Atif M et al., 2017	1	1	1	1	1	1	1	1	1	9
Atif M et al., 2016	0	1	1	1	1	1	0	1	0	6
Akhtar M et al., 2012	1	0	0	1	1	1	0	1	0	5
Henry CN et al., 2013	0	1	1	1	1	1	0	1	0	6
Angamo MT et al., 2011	1	1	1	1	1	1	0	1	1	8
Woldu MA et al., 2013	1	1	1	1	1	1	0	1	1	8
Al-Niemat SI et al., 2014	1	1	1	1	1	1	0	1	1	8
Mohajer KA et al., 2011	0	1	0	1	1	1	0	1	1	6
Wang H et al., 2013	1	1	1	0	1	1	0	1	0	6
Osowicki J et al., 2015	1	0	0	1	1	1	0	1	1	6
Amadeo B et al., 2010	1	1	0	1	1	1	1	1	1	8
Magill SS et al., 2014	1	1	1	1	1	1	1	1	1	9
Aldeyab MA et al., 2012	1	1	0	1	1	1	1	1	1	8
Talaat M et al., 2014	1	1	0	1	1	1	0	1	1	7
Akhloufi H et al., 2015	1	1	0	1	1	1	1	1	1	8
Sinatra I et al., 2013	1	0	0	1	1	0	1	1	1	6
Gizework A et al., 2015	1	0	0	1	1	0	0	1	0	4
Gandra S et al., 2017	1	1	0	1	1	1	0	1	1	7
Nsofor CA et al., 2016	1	1	0	1	1	1	0	1	1	7
Mulwa Charles N et al., 2015	1	1	1	1	1	1	0	1	0	7
Bashrahil KA et al., 2010	0	0	1	0	1	0	0	1	1	4
Mudenda W et al., 2016	1	1	1	1	1	0	0	1	0	6
Joda AE et al., 2013	1	1	1	0	1	0	0	1	1	6
Choudhury DK et al., 2013	1	0	0	0	1	1	0	1	1	5
Jose MW et al., 2016	1	1	0	1	1	1	0	1	1	7
Assen A et al., 2014	1	0	0	1	1	1	0	1	0	5
Li C et al., 2013	1	1	0	1	1	1	0	1	0	6
Versporten A et al., 2013	0	1	0	1	1	1	1	1	1	7
Sviestina I et al., 2015	1	1	0	1	1	1	1	1	1	8
Sviestina I et al., 2013	1	1	0	1	1	1	0	1	1	7
Bergicho M et al., 2012	1	0	1	1	1	1	0	1	1	7
Xie DS et al., 2015	0	1	0	1	1	1	0	1	1	6
Fadare J et al., 2015	0	0	1	1	1	1	0	1	1	6
Xie DS et al., 2015	1	1	0	1	1	1	0	1	1	7
Alfandari S et al., 2015	0	1	0	1	1	0	1	1	1	6
van Spreuwel PC et al., 2015	0	0	0	1	1	1	1	1	1	6
Cotta MO et al., 2014	1	1	0	1	1	0	0	1	1	6
Sviestina I et al., 2014	1	0	0	1	1	1	1	1	1	7
Osowicki J et al., 2014	1	1	0	1	1	1	0	1	1	7
Bozkurt F et al., 2014	0	1	0	1	1	1	1	1	1	7
Muyu G et al., 2013	0	0	0	1	1	1	0	1	1	5

Study	Q1	Q2	Q3	Q4	Q5	Q6	Q7	Q8	Q9	Total
Desalegn AA et al., 2013	1	1	1	1	1	1	0	1	1	8
Sozen H et al., 2013	0	0	0	1	1	1	1	1	1	6
Behnke M et al., 2013	1	1	1	1	1	0	1	1	1	8
Mabiala Babela JR et al., 2013	1	1	0	1	1	0	1	1	1	7
Borras Novell C et al., 2013	1	1	0	1	1	1	0	1	1	7
Zarb P et al., 2012	0	1	0	1	1	1	1	1	1	7
Thu TA et al., 2012	1	1	1	1	1	1	1	1	1	9
Ingram PR et al., 2012	0	0	0	1	1	1	0	1	1	5
Robert J et al., 2012	1	1	1	1	1	1	1	1	1	9
Levy ER et al., 2012	1	1	0	1	1	1	0	1	1	7
Sharma M et al., 2012	1	1	0	1	1	1	1	1	1	8
Baktygul K et al., 2011	1	1	0	1	1	1	1	1	1	8
Carneiro M et al., 2011	1	1	0	1	1	1	0	1	1	7
Pathak A et al., 2011	1	1	0	1	1	1	1	1	1	8
Xie DS et al., 2010	1	1	0	1	1	1	0	1	1	7
Ceyhan M et al., 2010	0	1	0	1	1	1	0	1	1	6
Willemsen I et al., 2010	1	1	0	1	1	1	0	1	1	7
Kolyva S et al., 2017	1	1	0	1	1	0	0	1	1	6
Larru B et al., 2016	0	0	0	1	1	1	0	1	1	5
Sharma S et al., 2016	1	1	1	1	1	1	0	1	1	8
Aly NY et al., 2012	1	1	1	1	1	1	0	1	1	8
Gailiene G et al., 2012	1	1	0	1	1	1	0	1	1	7
Askarian M et al., 2012	1	1	0	1	1	1	0	1	1	7
Dryden M et al., 2012	1	1	0	1	1	0	0	1	1	6
Gomez-Gomez J et al., 2015	1	1	0	0	1	1	0	1	1	6
Gugliotta C et al., 2020	0	0	0	1	1	1	0	1	1	5
Ababneh MA et al., 2021	1	1	0	1	1	1	1	1	1	8
Mijovic G et al., 2020	0	1	0	0	1	0	1	1	1	5
Wushouer H et al., 2020	0	1	1	1	1	1	0	1	1	7
Chui CSL et al., 2020	1	1	0	1	1	1	0	1	0	6
Murni IK et al., 2015	1	1	1	1	1	1	0	1	1	8
Korinteli IG et al., 2019	0	1	0	0	1	1	0	1	1	5
Gibbons CL et al., 2019	1	0	0	1	1	1	0	1	1	6
Okoro RN et al., 2019	0	1	1	1	1	1	0	1	1	7
Cole CP et al., 2018	1	1	1	0	1	0	0	1	0	5
Arnoldo L et al., 2019	1	1	0	1	1	0	0	1	1	6
Mao W et al., 2019	0	1	1	1	1	1	0	1	1	7
Nguyen S et al., 2018	1	1	0	1	1	0	0	1	1	6
Palms DL et al., 2018	0	1	0	1	1	1	0	1	1	6
Naughton C et al., 2011	0	1	0	1	1	1	0	1	1	6
Al-Azayzih A et al., 2017	1	1	0	1	1	1	0	1	1	7
Gerber JS et al., 2010	1	1	0	1	1	0	0	1	1	6

Study	Q1	Q2	Q3	Q4	Q5	Q6	Q7	Q8	Q9	Total
Ider BE et al., 2010	1	1	0	1	1	1	0	1	1	7
Arcavi L et al., 2010	1	1	0	1	1	0	1	1	1	7
Ahmed AM et al., 2010	0	1	1	1	1	1	0	1	0	6
Mohlala G et al., 2010	0	1	0	0	1	0	0	1	1	4
Evirgen O et al., 2011	1	0	0	0	1	0	1	1	1	5
Etienne P et al., 2011	1	0	0	0	1	0	1	1	1	5
Gwebu P et al., 2022	0	0	0	1	1	1	1	1	1	6
Gulmez SE et al., 2022	0	1	0	1	1	1	1	1	0	6
Guzeloglu E et al., 2022	1	1	0	1	1	1	0	1	1	7
Park SY et al., 2022	0	1	1	1	1	1	0	1	1	7
Kitt E et al., 2022	1	0	0	1	1	1	0	1	1	6
Yang Q et al., 2022	0	1	0	1	1	1	0	1	1	6
Talaat M et al., 2022	0	1	0	1	1	1	1	1	1	7
Sheikh S et al., 2022	0	0	0	1	1	1	1	1	1	6
Zhang JS et al., 2022	0	1	1	1	1	1	1	1	1	8
Shaikh Q et al., 2022	1	0	0	1	1	1	0	1	1	6
Oghuvwu S et al., 2023	0	1	0	1	1	1	0	1	0	5
Ranasinghe P et al., 2022	0	1	1	1	1	1	0	1	1	7
Akkawi ME et al., 2022	1	1	0	1	1	1	0	1	1	7
Nunez-Nunez M et al., 2022	1	1	0	1	1	1	0	1	1	7
Omulo S et al., 2022	1	1	0	1	1	1	0	1	1	7
Gutiérrez-Urbón J et al., 2022	0	1	0	1	1	1	1	1	1	7
Joshi R et al., 2022	0	1	0	1	1	1	0	1	1	6
Nguyen HQ et al., 2022	1	1	0	1	1	1	1	1	1	8
Sharif M et al., 2022	1	1	0	0	1	0	0	1	1	5
Versporten A et al., 2018	0	1	0	1	1	1	1	1	0	6
Saleem Z et al., 2022	0	1	1	1	1	1	1	1	1	8
Limato R et al., 2021	1	1	0	1	1	1	1	1	1	8
Ishibashi N et al., 2022	0	1	0	1	1	1	1	1	1	7
Jamaluddin NAH et al., 2021	1	0	0	1	1	1	0	1	1	6
Ashour RH et al., 2022	1	0	0	1	1	1	0	1	1	6
Panditrao AM et al., 2021	0	1	0	1	1	1	1	1	1	7
Rashid MM et al., 2022	1	1	0	1	1	1	0	1	1	7
Ankrah D et al., 2021	1	1	0	1	1	1	1	1	1	8
D'Arcy N et al., 2021	1	1	0	0	1	1	1	1	1	7
Kiggundu R et al., 2022	0	1	0	1	1	1	0	1	1	6
Wang CN et al., 2021	1	1	0	1	1	1	1	1	1	8
Rocke T et al., 2022	0	0	0	1	1	1	0	1	1	5
Oo WT et al., 2022	0	1	0	1	1	1	1	1	1	7
Spernovasilis N et al., 2022	1	0	0	1	1	1	1	1	1	7
Okoye BI et al., 2022	0	1	0	1	1	1	0	1	1	6
El-Dahiyat F et al., 2022	1	0	0	1	1	1	0	1	0	5

Study	Q1	Q2	Q3	Q4	Q5	Q6	Q7	Q8	Q9	Total
Mittal N et al., 2023	1	1	0	1	1	1	0	1	1	7
Sidamo T et al., 2022	1	1	0	1	1	1	0	1	1	7
Tadesse TY et al., 2022	1	1	0	1	1	1	0	1	1	7
Sodhi B et al., 2022	1	0	0	1	1	1	0	1	1	6
Amponsah OKO et al., 2022	1	1	0	1	1	1	0	1	1	7
Skosana PP et al., 2022	1	1	0	1	1	1	1	1	1	8
Yimer YS et al., 2022	0	1	0	1	1	1	0	1	1	6
Deiana G et al., 2022	0	1	0	1	1	1	0	1	1	6
Meenakshi R et al., 2022	1	1	0	1	1	1	0	1	1	7
Abu Farha R et al., 2022	1	1	0	1	1	1	0	1	1	7
Alnajjar MS et al., 2022	1	0	0	1	1	1	0	1	1	6
Anugulruengkitt S et al., 2022	0	1	1	1	1	1	0	1	1	7
Parathoduvil AA et al., 2022	1	1	0	1	1	1	0	1	0	6
Kalungia AC et al., 2022	1	0	0	1	1	1	1	1	1	7
Catho G et al., 2022	1	1	0	1	1	1	0	1	1	7
Chansamouth V et al., 2022	1	1	1	1	1	1	0	1	1	8
Usluer G et al., 2005	0	1	1	0	1	0	0	1	0	4
Ufer M et al., 2005	0	1	0	0	1	0	1	1	1	5
McGregor JC et al., 2006	1	1	0	1	1	1	0	1	1	7
Avci IY et al., 2006	0	1	0	0	1	0	0	1	1	4
Likic R et al., 2007	0	1	0	0	1	1	0	1	0	4
Seaton RA et al., 2007	0	1	0	1	1	0	0	1	1	5
Lee MK et al., 2007	1	1	0	0	1	0	0	1	1	5
Vlahovic-Palcevski V et al., 2007	0	1	0	1	1	0	1	1	0	5
Al-Niemat SI et al., 2008	0	1	0	0	1	1	0	1	0	4
Akande TM et al., 2007	0	0	0	1	1	0	0	1	1	4
Oshikoya KA et al., 2007	1	1	0	0	1	0	0	1	1	5
Danchaivijitr S et al., 2007	1	1	1	0	1	0	0	1	0	5
Hajdu A et al., 2007	1	0	0	1	1	0	0	1	1	5
Tunger O et al., 2008	0	1	0	0	1	0	0	1	0	3
Dimina E et al., 2009	0	1	1	1	1	1	1	1	1	8
de With K et al., 2009	0	1	0	1	1	0	1	1	1	6
Ciofi Degli Atti ML et al., 2008	1	0	0	1	1	0	0	1	1	5
Hariharan S et al., 2009	1	1	0	1	1	1	0	1	1	7
Dimri S et al., 2009	1	0	0	0	1	0	0	1	1	4
Newman MJ et al., 2009	0	1	0	0	1	0	0	1	0	3
Ghimire S et al., 2009	0	1	0	1	1	0	0	1	0	4
Calligaris L et al., 2009	0	1	0	1	1	1	1	1	1	7
Ansari F et al., 2009	1	1	0	1	1	0	1	1	1	7
Rivero M et al., 2000	1	1	0	0	1	0	0	1	1	5
Erbay A et al., 2003	1	1	0	0	1	0	0	1	1	5
Potocki M et al., 2003	1	0	0	0	1	0	0	1	1	4

Study	Q1	Q2	Q3	Q4	Q5	Q6	Q7	Q8	Q9	Total
Mora Y et al., 2002	0	0	0	0	1	0	0	1	1	3
Raveh D et al., 2001	1	1	0	0	1	0	0	1	1	5
Tunger O et al., 2000	0	1	0	0	1	0	0	1	1	4
Berild D et al., 2002	1	1	0	0	1	0	1	1	1	6
McDonald LC et al., 2001	1	1	0	0	1	0	0	1	1	5
Berild D et al., 2002	1	0	0	0	1	0	0	1	1	4
Apisarnthanarak A et al., 2006	1	0	0	0	1	0	0	1	1	4
Karande S et al., 2005	1	0	0	0	1	0	0	1	1	4
Fonseca LG et al., 2004	1	0	0	0	1	0	0	1	1	4
Starakis I et al., 2002	1	1	0	0	1	0	0	1	1	5
Chukwuani CM et al., 2002	1	0	0	1	1	0	0	1	0	4
Gikas A et al., 2002	0	1	0	1	1	0	0	1	1	5
Kanerva M et al., 2007	1	1	1	1	1	0	1	1	1	8
Willemsen I et al., 2007	1	1	0	0	1	0	0	1	1	5
Shankar PR et al., 2006	1	0	0	1	1	0	0	1	1	5
Desai NM et al., 2020	1	0	1	1	1	0	0	1	1	6
Anteneh DA et al., 2021	1	1	1	1	1	1	0	1	1	8
Cotter JM et al., 2022	1	0	1	1	1	1	0	1	1	7
Dilworth TJ et al., 2022	1	0	0	0	1	0	0	1	1	4
Kroening-Roche JC et al., 2012	1	0	0	1	1	1	0	1	0	5
Linder JA et al., 2003	1	1	0	1	1	1	0	1	1	7
Kornblith AE et al., 2018	1	1	1	1	1	1	1	1	1	9
Kozyrskyj AL et al., 2004	1	0	1	1	1	1	0	1	1	7
Curt AM et al., 2020	1	0	0	1	1	1	0	1	0	5
Kumar R et al., 2008	1	1	1	0	1	1	0	1	0	6
Cantrell R et al., 2002	1	0	1	1	1	1	1	1	1	8
Arnold SR et al., 2005	1	1	1	1	0	1	0	1	0	6
Cadieux G et al., 2011	1	0	1	1	1	1	1	1	1	8
Barnett ML et al., 2014	1	0	1	1	1	1	1	1	1	8
Bergmark RW et al., 2016	1	0	1	0	1	1	1	1	1	7
Depew RE et al., 2020	1	0	1	1	1	1	1	1	1	8
Coco AS et al., 2009	1	0	1	1	1	1	1	1	1	8
Copp HL et al., 2011	1	0	1	1	1	1	1	1	1	8
Ababneh MA et al., 2017	1	0	0	1	1	1	0	1	1	6
Chang LY et al., 2017	1	0	1	1	1	1	1	1	1	8
Ardoino I et al., 2019	1	0	1	1	1	1	1	1	1	8
Covino M et al., 2022	1	0	0	1	1	1	0	1	1	6
Kourlaba G et al., 2016	1	0	1	1	1	1	1	1	1	8
Lin YC et al., 2010	1	0	1	1	1	1	1	1	1	8
Kronman MP et al., 2011	1	0	1	1	1	1	1	1	1	8
Forster CS et al., 2022	1	0	1	1	1	1	1	1	1	8
Goodman KE et al., 2023	1	0	1	1	1	1	1	1	1	8

Study	Q1	Q2	Q3	Q4	Q5	Q6	Q7	Q8	Q9	Total
Hadi U et al., 2008	1	0	1	1	1	1	1	1	1	8
Hersh AL et al., 2011	1	0	1	1	1	1	1	1	1	8
Kawanami GH et al., 2011	1	0	0	1	1	1	0	1	1	6
Jewell MJ et al., 2021	1	0	1	1	1	1	1	1	1	8
Hashimoto H et al., 2019	1	0	1	1	1	1	1	1	1	8
Hadi U et al., 2008	1	0	0	1	1	1	0	1	1	6
Stone S et al., 2000	1	0	1	1	1	1	1	1	1	8
Vanderweil SG et al., 2008	1	0	1	1	1	1	1	1	1	8
Shapiro DJ et al., 2014	1	0	1	1	1	1	1	1	1	8
Sencan I et al., 2022	1	0	1	1	1	1	0	1	1	7
Shin SM et al., 2015	1	0	1	1	1	1	1	1	1	8
Zhang Z et al., 2017	1	1	1	1	1	1	0	1	1	8
Teixeira Rodrigues A et al., 2016	1	0	1	1	0	1	1	1	1	7
Velasco E et al., 2011	1	1	1	1	0	1	0	1	1	7
Havers FP et al., 2018	1	0	1	1	1	1	0	1	1	7
Salzo A et al., 2021	1	0	0	1	1	1	1	1	1	7
Opoku MM et al., 2020	1	0	0	1	1	1	1	1	1	7
Morley VJ et al., 2020	1	0	0	0	1	1	0	1	1	5
McKay R et al., 2019	1	0	1	1	1	1	1	1	1	8
Manne M et al., 2018	1	0	1	1	1	1	1	1	1	8
Paul IM et al., 2011	1	0	1	1	1	1	1	1	1	8
Moro ML et al., 2009	1	0	1	1	1	0	0	1	1	6
Osatakul S et al., 2007	1	1	1	1	1	1	0	1	1	8
Rutschmann OT et al., 2004	1	0	1	1	1	1	1	1	1	8
Nadeem Ahmed M et al., 2010	1	0	1	1	1	1	1	1	1	8
Aspinall SL et al., 2009	1	0	0	1	1	1	0	1	1	6
Ahmad A et al., 2021	1	0	0	1	0	1	0	1	1	5
Sawaya RD et al., 2020	1	0	0	1	1	1	0	1	1	6
Steinberg MB et al., 2016	1	0	1	1	1	1	1	1	1	8
Zhao H et al., 2020	1	0	1	1	1	1	1	1	1	8

Supplementary Table 7. Inappropriate antibiotic prescribing of included studies

Table S7. Inappropriate antibiotic prescribing of included studies

Study	Country	Standards for prescriptions evaluation	Medical setting	Events*	Total†
Fentie AM et al., 2022 [1]	Ethiopia	According to WHO's definition of guideline compliance.	Inpatient	525	1162
Levy HG et al., 2022 [2]	Cuba	Compliance with the prescribed antibiotics (drug, dose, interval, route of administration) with clinical practice guidelines (CPGs).	Inpatient	238	695
	Mexico			121	442
	El Salvador			405	1332
	Peru			251	873
	Paraguay			183	474
Moulin E et al., 2022 [3]	Switzerland	The appropriateness of antimicrobial use is assessed by indication, duration, route of administration, spectrum, and dose.	Inpatient	106	182
Xavier SP et al., 2022 [4]	Mozambique	Antibiotics were considered inappropriately used in five cases: (1) combination of antibiotics of the same class (duplication); (2) wrong antibiotic selected for the disease; (3) non-recommended route of administration; (4) wrong duration of antibiotic use; and (5) dosage error, in which the prescribed dose was higher or lower and short or long duration of treatment than recommended by international guidelines and databases.	Inpatient	162	464
Haseeb A et al., 2021 [5]	Saudi Arabia	Compliance was measured by assessing prescribing patterns against institutional antimicrobial prescribing guidelines.	Inpatient	548	773

Study	Country	Standards for prescriptions evaluation	Medical setting	Events*	Total†
Zhao H et al., 2021 [6]	China	Outpatient visits with antibiotic prescriptions were assigned into one of the four mutually exclusive categories as applied by Chua and colleagues: appropriate if associated with tier 1 diagnosis, potentially appropriate if associated with tier 2 but not tier 1 diagnosis, inappropriate if associated with only tier 3 diagnosis, and not linked to any diagnosis if not associated with any visit-level diagnosis.	Outpatient	9689937	18848864
Almansoori N et al., 2021 [7]	South Africa	To evaluate the prescriber adherence to guidelines, The Standard Treatment Guidelines and Essential Drug List (EDL) for South Africa Adult Hospital Level Edition 2015 were used as a reference.	Outpatient	94	189
Yehualaw A et al., 2021 [8]	Ethiopia	Data were collected using a pretested checklist. World Health Organization Guideline for Pediatric Illness and Ethiopia Standard Treatment Guideline for General Hospitals were used to assess the rational use and prescribing pattern of antibiotics.	Inpatient	196	692
Momanyi L et al., 2019 [9]	Kenya	Prescriptions are guided by the Kenyan Essential Medicine List and relevant international guidelines.	Inpatient	193	357
Mama M et al., 2020 [10]	Ethiopia	Antibiotic choice, dosage form, dose, frequency, and duration were compared against the Ethiopian national treatment guideline to check antibiotic appropriateness.	Inpatient	141	471
Rachina S et al., 2020 [11]	Russia	The prescription of AMD in clinical practice was evaluated by means of quality indicators specified by the Global-PPS international study protocol.	Inpatient	998	3542
Charani E et al., 2019 [12]	England	The assessment criteria are local empiric policies. Antibiotic therapy was flagged as non-compliant if it was not in line with the recommendations in the policy documenting the indication and if there was no input from the infection team.	Inpatient	65	304
Gürtler N et al., 2019 [13]	Switzerland	The appropriateness of antimicrobial use was evaluated according to previously published rules and local or national guidelines.	Inpatient	182	548
Sheng T et al., 2019 [14]	Sri Lanka	Potentially inappropriate antimicrobial use was defined as (1) antimicrobial use	Inpatient	290	935

Study	Country	Standards for prescriptions evaluation	Medical setting	Events*	Total†
		discordant with the Sri Lanka College of Microbiologists' guidelines for common indications and (2) redundant combinations of antimicrobials.			
Komagamine J et al., 2019 [15]	Japan	The appropriateness of antimicrobial drug use for treatment was determined by evaluating the following points: (1) Indication: Does the patient have an infection that needs antimicrobial drugs? (2) Dose and timing: Is the dose or timing of the antimicrobial drug appropriate? (3) Duration: Is the duration of antimicrobial therapy appropriate? (4) Choice: Is the selected antimicrobial drug effective? (5) Spectrum: Are there no alternative antimicrobial drugs that are equally effective and have narrower spectrum activity? Antimicrobial therapy was judged to be appropriate if the answer to all five questions was 'yes'.	Inpatient	62	163
Masich AM et al., 2020 [16]	Zambia	To evaluate antibiotic appropriateness as a composite measure of correct dose, frequency, route, duration, and spectrum of coverage for each indication, Infectious Diseases Society of America (IDSA) guidelines, WHO guidelines, and local infectious diseases clinical expertise were utilized to determine all possible effective antimicrobial treatment regimens based on indication, site of infection, and antimicrobial availability.	Inpatient	59	88
Maina M et al., 2020 [17]	Kenya	Treatment appropriateness is defined as any prescription which is in keeping with (1) treatment guidelines, (2) consensus of local expert opinion, or (3) bacterial speciation and antibiotic susceptibility tests.	Inpatient	697	1502
Chautrakarn S et al., 2020 [18]	Thailand	The appropriateness of the use was evaluated by two infectious diseases pediatricians based on available local hospital guidelines.	Inpatient	193	279
Mohammed ZA et al., 2020 [19]	India	The appropriateness of the antibiotic dose was decided based on standard recommendations by The Sanford Guide to antimicrobial therapy.	Inpatient	51	188
Gardiner SJ et al., 2020 [20]	New Zealand	The assessment of antimicrobial use is based on the Australian National Antimicrobial Prescribing Survey (http://naps.org.au) against local guidelines and	Inpatient	76	451

Study	Country	Standards for prescriptions evaluation	Medical setting	Events*	Total†
		national funding criteria.			
Cusini A et al., 2010 [21]	Switzerland	The appropriateness of antimicrobial prescriptions was evaluated according to local and international evidence-based guidelines, and considering local epidemiology of antimicrobial resistance, microbiological findings, if available, and co-morbidity.	Inpatient	406	1270
Denny KJ et al., 2019 [22]	Australia	The experts utilized evidence-based guidelines together with a pre-established antibiotic appropriateness assessment tool, the National Antibiotic Prescribing Survey (NAPS) table.	Outpatient	333	1019
Sikkens JJ et al., 2018 [23]	Netherlands	This study defined appropriate antimicrobial therapy as the prescription of an antimicrobial agent satisfying any one of the following three conditions on the day of the survey: (1) it followed relevant guidelines; (2) it deviated from the relevant guidelines but rational arguments for deviation were documented in the patient file or supplied by the ward physician; and (3) there was no relevant guideline but the prescription was considered a rational choice. A rational choice was defined as an effective antimicrobial drug covering relevant pathogens without an excessively broad antimicrobial spectrum, long duration, high or low dosage, or incorrect route of administration (e.g. omission to switch to oral therapy if adequate or possible).	Inpatient	123	351
Croche Santander B et al., 2018 [24]	Spain	To assess the suitability of antibiotic prescriptions, the clinical practice was compared with an evidence-based guideline especially designed for this study.	Outpatient	54	104
Wang S et al., 2018 [25]	China	To assess the suitability of antibiotic prescriptions, the clinical practice was compared with an evidence-based guideline especially designed for this study.	Outpatient	403	2089
Goycochea-Valdivia WA et al., 2017 [26]	Spain	Appropriateness of antimicrobial prescription (AAP) was defined as a correct indication plus an appropriate prescribing pattern (dose, spectrum, and interval).	Inpatient	91	161
Knezevic B et al., 2016 [27]	Australia	An internally validated scoring system.	Inpatient	84	279

Study	Country	Standards for prescriptions evaluation	Medical setting	Events*	Total†
Osowicki J et al., 2015 [28]	Australia	Prescriptions were deemed appropriate or inappropriate in accordance with clinical and microbiological findings and institutional guidelines (where available) and coded on a standard template across all participating centers considering: (1) the decision to prescribe an antimicrobial; (2) the choice of antimicrobial (compliance with hospital guidelines, appropriate antimicrobial spectrum) and (3) the application of the antimicrobial prescription in regards to dose, interval, route, and duration.	Inpatient	9	216
Akhloufi H et al., 2015 [29]	Netherlands	The appropriateness of antibiotic therapy was determined for each individual patient by both a clinical microbiologist and an infectious disease consultant, using the standardized method developed by Gyssens et al.	Inpatient	90	307
Cotta MO et al., 2014 [30]	Australia	Appropriateness' of antimicrobial therapy was evaluated by a team consisting of an infectious diseases physician and a specialist infectious diseases pharmacist based on clear criteria.	Inpatient	389	1444
Osowicki J et al., 2014 [31]	Australia	Appropriateness was assessed on the basis of the clinical scenario, including microbiological findings, institutional antimicrobial resistance patterns, and institutional treatment guidelines, where available.	Inpatient	177	631
Ingram PR et al., 2012 [32]	Australia	The appropriateness of antibiotic therapy was determined based on a standardized method developed by Willemsen et al.	Inpatient	123	262
Ceyhan M et al., 2010 [33]	Turkey	Antimicrobial drug use was considered inappropriate if the indication and choice of the drug(s) or the dose of the antimicrobials were wrong. Information from the guidelines of the American Academy of Pediatrics and classical textbooks was used in this evaluation.	Inpatient	332	711
Kolyva S et al., 2017 [34]	Greece	Guidelines for the treatment of various diseases.	Outpatient	76	940
Aly NY et al., 2012 [35]	Kuwait	Aspects of the prescribed antibiotic were benchmarked to the hospital antibiotic policy guidelines to evaluate adherence.	Inpatient	306	1112

Study	Country	Standards for prescriptions evaluation	Medical setting	Events*	Total†
Gomez-Gomez J et al., 2015 [36]	Austria	The Infectious Diseases Society of America (IDSA) and the Spanish Society of Infectious Diseases and Clinical Microbiology (SEIMC).	Inpatient	259	602
Murni IK et al., 2015 [37]	Indonesia	WHO Pocket Book of Hospital Care for Children.	Inpatient	336	780
Etienne P et al., 2011 [38]	France	Guidelines for the treatment of various diseases.	Inpatient	35	102
Kitt E et al., 2022 [39]	Botswana	Local, national, and international guidelines.	Inpatient	63	119
Yang Q et al., 2022 [40]	China	(1) Drug description.	Outpatient	1345	62289
		(2) Prescribing point review management practices (Trial), 2010, China.			
		(3) Scheme of special remediation activity on the clinical application of antimicrobial drugs, 2012, China.			
		Guidelines for clinical use of antimicrobial agents, 2015, China.			
		Clinical guidelines.			
		UpToDate database (Wolters Kluwer, Alphen aan den Rijn, The Netherlands).			
Akkawi ME et al., 2022 [41]	Malaysia	The national antibiotic guidelines (NAG) of 2014 and 2019 were used to assess the appropriateness of the prescribed antibiotics.	Inpatient	169	670
Nunez-Nunez M et al., 2022 [42]	Spain	Antibiotic prescriptions were classified as "inappropriate" if the drug, route, dose, and/or duration of the antibiotic prescription were incorrect according to the local guideline.	Inpatient	817	1600

* Events: The number of inappropriate prescriptions containing antibiotics or the number of patients prescribed inappropriate antibiotics.

† Total: Total number of prescriptions containing antibiotics or Total number of patients prescribed antibiotics.

References for included studies

1. Fentie AM, Degefaw Y, Asfaw G, Shewarega W, Woldearegay M, Abebe E, et al. Multicentre point-prevalence survey of antibiotic use and healthcare-associated infections in Ethiopian hospitals. Bmj Open. 2022;12(2):e54541.
2. Levy HG, Rojas-Cortes R, Molina LH, Dreser MA, Alfonso OI, Rizo-Amezquita JN, et al. Point prevalence survey of antibiotic use in hospitals in Latin American

countries. J Antimicrob Chemoth. 2022;77(3):807-15.
3. Moulin E, Boillat-Blanco N, Zanetti G, Plüss-Suard C, De Vallière S, Senn L. Point prevalence study of antibiotic appropriateness and possibility of early discharge from hospital among patients treated with antibiotics in a Swiss university hospital. Antimicrobial Resistance & Infection Control. 2022;11(1):1-8.
4. Xavier SP, Victor A, Cumaquela G, Vasco MD, Rodrigues O. Inappropriate use of antibiotics and its predictors in pediatric patients admitted at the Central Hospital of Nampula, Mozambique. Antimicrob Resist in. 2022;11(1):79.
5. Haseeb A, Faidah HS, Algethamy M, Alghamdi S, Alhazmi GA, Alshomrani AO, et al. Antimicrobial usage and resistance in Makkah region hospitals: a regional point prevalence survey of public hospitals. Int J Env Res Pub He. 2021;19(1):254.
6. Zhao H, Wei L, Li H, Zhang M, Cao B, Bian J, et al. Appropriateness of antibiotic prescriptions in ambulatory care in China: a nationwide descriptive database study. Lancet Infect Dis. 2021;21(6):847-57.
7. Almansoori N, Parag N. Antibiotic prescribing patterns in emergency department at regional hospital in South Africa. Afr Health Sci. 2021;21(4):1651-61.
8. Yehualaw A, Taferre C, Bantie AT, Demsie DG. Appropriateness and pattern of antibiotic prescription in pediatric patients at Adigart General Hospital, Tigray, Ethiopia. Biomed Res Int. 2021;2021:6640892.
9. Momanyi L, Opanga S, Nyamu D, Oluka M, Kurdi A, Godman B. Antibiotic prescribing patterns at a leading referral hospital in Kenya: a point prevalence survey. J Res Pharm Pract. 2019;8(3):149-54.
10. Mama M, Mamo A, Usman H, Hussen B, Hussen A, Morka G. Inappropriate antibiotic use among inpatients attending Madda Walabu University Goba Referral Hospital, southeast Ethiopia: implication for future use. Infect Drug Resist. 2020;13:1403-9.
11. Rachina S, Belkova Y, Kozlov R, Versporten A, Pauwels I, Goossens H, et al. Longitudinal point prevalence survey of antimicrobial consumption in Russian hospitals: results of the Global-PPS project. Antibiotics-Basel. 2020;9(8):446.
12. Charani E, de Barra E, Rawson TM, Gill D, Gilchrist M, Naylor NR, et al. Antibiotic prescribing in general medical and surgical specialties: a prospective cohort study. Antimicrob Resist in. 2019;8(151):1-10.
13. Gürtler N, Erba A, Giehl C, Tschudin-Sutter S, Bassetti S, Osthoff M. Appropriateness of antimicrobial prescribing in a Swiss tertiary care hospital: a repeated point prevalence survey. Swiss Med Wkly. 2019;149:w20135.
14. Sheng T, Wijayaratne GB, Dabrera TM, Drew RJ, Nagahawatte A, Bodinayake CK, et al. Point-prevalence study of antimicrobial use in public hospitals in southern Sri Lanka identifies opportunities for improving prescribing practices. Infect Cont Hosp Ep. 2019;40(2):224-7.
15. Komagamine J, Yabuki T, Hiraiwa T. A trend in prevalence of antimicrobial use and appropriateness of antimicrobial therapy in an acute care hospital from 2018 to 2019: repeated prevalence surveys in Japan. Bmc Res Notes. 2019;12(1):811.

16. Masich AM, Vega AD, Callahan P, Herbert A, Fwoloshi S, Zulu PM, et al. Antimicrobial usage at a large teaching hospital in Lusaka, Zambia. Plos One. 2020;15(2):e228555.
17. Maina M, Mwaniki P, Odira E, Kiko N, McKnight J, Schultsz C, et al. Antibiotic use in Kenyan public hospitals: prevalence, appropriateness and link to guideline availability. Int J Infect Dis. 2020;99:10-8.
18. Chautrakarn S, Anugulruengkitt S, Puthanakit T, Rattananupong T, Hiransuthikul N. Antimicrobial prescription patterns in a tertiary-care pediatric unit in Thailand. Pediatr Int. 2020;62(6):683-7.
19. Mohammed ZA, Mukhopadhyay C, Varma M, Kalwaje EV. Identifying opportunities for antimicrobial stewardship through a point prevalence survey in an Indian tertiary-care teaching hospital. J Glob Antimicrob Re. 2020;23:315-20.
20. Gardiner SJ, Basevi AB, Hamilton NL, Metcalf SC, Chambers ST, Withington SG, et al. Point prevalence surveys of antimicrobial use in adult inpatients at Canterbury District Health Board Hospitals. New Zeal Med J. 2020;133(1525):18-33.
21. Cusini A, Rampini SK, Bansal V, Ledergerber B, Kuster SP, Ruef C, et al. Different patterns of inappropriate antimicrobial use in surgical and medical units at a tertiary care hospital in Switzerland: a prevalence survey. Plos One. 2010;5(11):e14011.
22. Denny KJ, Gartside JG, Alcorn K, Cross JW, Maloney S, Keijzers G. Appropriateness of antibiotic prescribing in the emergency department. J Antimicrob Chemoth. 2019;74(2):515-20.
23. Sikkens JJ, Gerritse SL, Peters E, Kramer M, van Agtmael MA. The 'morning dip' in antimicrobial appropriateness: circumstances determining appropriateness of antimicrobial prescribing. J Antimicrob Chemoth. 2018;73(6):1714-20.
24. Croche SB, Campos AE, Sanchez CA, Marcos FL, Diaz FI, Vargas JC, et al. Appropriateness of antibiotic prescribing in paediatric patients in a hospital emergency department. An Pediatr (Engl Ed). 2018;88(5):259-65.
25. Wang S, Guan L, Dong C, Ji Y. Evaluation of the drug effect and rational use of antiseptic drugs in outpatient and emergency department of hospital. Pak J Pharm Sci. 2018;31(4(Special)):1701-5.
26. Goycochea-Valdivia WA, Moreno-Ramos F, Pano-Pardo JR, Aracil-Santos FJ, Baquero-Artigao F, Del RT, et al. Identifying priorities to improve paediatric in-hospital antimicrobial use by cross-sectional evaluation of prevalence and appropriateness of prescription. Enferm Infec Micr Cl. 2017;35(9):556-62.
27. Knezevic B, Sprigg D, Seet J, Trevenen M, Trubiano J, Smith W, et al. The revolving door: antibiotic allergy labelling in a tertiary care centre. Intern Med J. 2016;46(11):1276-83.
28. Osowicki J, Gwee A, Noronha J, Britton PN, Isaacs D, Lai TB, et al. Australia-wide point prevalence survey of antimicrobial prescribing in neonatal units: how much and how good? Pediatr Infect Dis J. 2015;34(8):e185-90.

29. Akhloufi H, Streefkerk RH, Melles DC, de Steenwinkel JE, Schurink CA, Verkooijen RP, et al. Point prevalence of appropriate antimicrobial therapy in a Dutch university hospital. Eur J Clin Microbiol. 2015;34(8):1631-7.
30. Cotta MO, Robertson MS, Upjohn LM, Marshall C, Liew D, Buising KL. Using periodic point-prevalence surveys to assess appropriateness of antimicrobial prescribing in Australian private hospitals. Intern Med J. 2014;44(3):240-6.
31. Osowicki J, Gwee A, Noronha J, Palasanthiran P, McMullan B, Britton PN, et al. Australia-wide point prevalence survey of the use and appropriateness of antimicrobial prescribing for children in hospital. Med J Australia. 2014;201(11):657-62.
32. Ingram PR, Seet JM, Budgeon CA, Murray R. Point-prevalence study of inappropriate antibiotic use at a tertiary Australian hospital. Intern Med J. 2012;42(6):719-21.
33. Ceyhan M, Yildirim I, Ecevit C, Aydogan A, Ornek A, Salman N, et al. Inappropriate antimicrobial use in Turkish pediatric hospitals: a multicenter point prevalence survey. Int J Infect Dis. 2010;14(1):e55-61.
34. Kolyva S, Gkentzi D, Koulouri A, Dimitriou G. Antibiotic prescribing in the pediatric emergency department. J Chemotherapy. 2017;29(4):257-60.
35. Aly NY, Omar AA, Badawy DA, Al-Mousa HH, Sadek AA. Audit of physicians' adherence to the antibiotic policy guidelines in Kuwait. Med Prin Pract. 2012;21(4):310-7.
36. Gomez-Gomez J, Garcia-Vazquez E, Bonillo C, Hernandez-Torres A, Canteras-Jordana M. Use of antibiotics at a university clinic hospital: effect of protocolized antibiotic treatment in the evolution of hospital patients with infections. Rev Esp Quim. 2015;28(6):302-9.
37. Murni IK, Duke T, Kinney S, Daley AJ, Soenarto Y. Reducing hospital-acquired infections and improving the rational use of antibiotics in a developing country: an effectiveness study. Arch Dis Child. 2015;100(5):454-9.
38. Etienne P, Roger PM, Brofferio P, Labate C, Blanc V, Tiger F, et al. Antimicrobial stewardship program and quality of antibiotic prescriptions. Med Maladies Infect. 2011;41(11):608-12.
39. Kitt E, Hayes M, Ballester L, Sewawa KB, Mulale U, Mazhani L, et al. Assessing antibiotic utilization among pediatric patients in Gaborone, Botswana. Sage Open Med. 2022;10:1-10.
40. Yang Q, Yuan F, Li L, Jin J, He J. Effects of monthly evaluations on the rates of irrational antimicrobial prescription in the outpatient and emergency departments at Ningbo No. 6 Hospital, Ningbo, China. Eur J Med Res. 2022;27(1):98-104.
41. Akkawi ME, Taffour RM, AL-Shami AM. Evaluation of antibiotic prescribing pattern and appropriateness among hospitalized pediatric patients: findings from a Malaysian teaching hospital. Infect Dis Rep. 2022;14(6):889-99.
42. Nunez-Nunez M, Perez-Galera S, Antonio Giron-Ortega J, Sandoval Fernandez-Del-Castillo S, Beltran-Garcia M, De Cueto M, et al. Predictors of inappropriate antimicrobial prescription: eight-year point prevalence surveys experience in a third level hospital in Spain. Front Pharmacol. 2022;13:1018158.

Supplementary Table 8. Antibiotic prescribing on the AWaRe classification

Table S8. Antibiotic prescribing on the AWaRe classification

Study	Medical setting	The AWaRe group	The number of prescriptions including antibiotics in the Access, Watch, or Reserve group	Total number of prescriptions
Levy HG et al., 2022 [1]	Inpatient	Access	2482	4302
		Watch	1720	
		Reserve	18	
Moulin E et al., 2022 [2]	Inpatient	Access	51	120
		Watch	42	
		Reserve	0	
Magill SS et al., 2021 [3]	Inpatient	Access	3323	18452
		Watch	8864	
		Reserve	291	
Mustafa ZU et al., 2022 [4]	Inpatient	Access	606	1224
		Watch	557	
		Reserve	0	
Yi S et al., 2022 [5]	Outpatient	Access	414	1212
		Watch	673	
		Reserve	0	
Haseeb A et al., 2021 [6]	Inpatient	Access	160	773
		Watch	365	
		Reserve	0	
Ogunleye OO et al., 2022 [7]	Inpatient	Access	122	744
		Watch	619	
		Reserve	1	
Boone K et al., 2021 [8]	Inpatient	Access	339	569
		Watch	224	
		Reserve	6	
Kurdi A et al., 2021 [9]	Inpatient	Access	75	266
		Watch	165	
		Reserve	26	
Skosana PP et al., 2021 [10]	Inpatient	Access	1203	2204
		Watch	666	
		Reserve	42	
Blackburn J et al., 2021 [11]	Inpatient	Access	213	489
		Watch	313	
		Reserve	13	
Pauwels I et al., 2021 [12]	Inpatient	Access	42509	106105

Study	Medical setting	The AWaRe group	The number of prescriptions including antibiotics in the Access, Watch, or Reserve group	Total number of prescriptions
		Watch	60501	
		Reserve	2099	
Tassew SG et al., 2021 [13]	Inpatient	Access	424	774
		Watch	350	
		Reserve	0	
Yehualaw A et al., 2021 [14]	Inpatient	Access	139	320
		Watch	182	
		Reserve	0	
Wendie TF et al., 2021 [15]	Outpatient	Access	402	660
		Watch	181	
		Reserve	0	
de Guzman Betito G et al., 2021 [16]	Inpatient	Access	247	1010
		Watch	746	
		Reserve	16	
Momanyi L et al., 2019 [17]	Inpatient	Access	165	357
		Watch	142	
		Reserve	0	
Soltani J et al., 2018 [18]	Inpatient	Access	127	391
		Watch	234	
		Reserve	0	
Saleem Z et al., 2019 [19]	Inpatient	Access	228	1943
		Watch	1715	
		Reserve	0	
Al Matar M et al., 2019 [20]	Inpatient	Access	776	2974
		Watch	2091	
		Reserve	106	
Komagamine J et al., 2019 [21]	Inpatient	Access	40	79
		Watch	16	
		Reserve	0	
Yimenu DK et al., 2019 [22]	Inpatient	Access	305	567
		Watch	264	
		Reserve	0	
Komagamine J et al., 2019 [23]	Inpatient	Access	42	163
		Watch	96	
		Reserve	0	
Komagamine J et al., 2020 [24]	Outpatient	Access	48	162
		Watch	69	
		Reserve	0	
Masich AM et al., 2020 [25]	Inpatient	Access	15	122
		Watch	107	

Study	Medical setting	The AWaRe group	The number of prescriptions including antibiotics in the Access, Watch, or Reserve group	Total number of prescriptions
		Reserve	0	
Khan Z et al., 2020 [26]	Outpatient	Access	90	350
		Watch	260	
		Reserve	0	
Yilma Z et al., 2020 [27]	Outpatient	Access	134	445
		Watch	281	
		Reserve	0	
Seni J et al., 2020 [28]	Inpatient	Access	425	1032
		Watch	589	
		Reserve	0	
Abubakar U et al., 2020 [29]	Inpatient	Access	148	449
		Watch	299	
		Reserve	0	
Horumpende PG et al., 2020 [30]	Inpatient	Access	132	330
		Watch	179	
		Reserve	0	
Mohammed ZA et al., 2020 [31]	Inpatient	Access	46	188
		Watch	133	
		Reserve	9	
Loftus MJ et al., 2020 [32]	Inpatient	Access	183	325
		Watch	70	
		Reserve	0	
Gardiner SJ et al., 2020 [33]	Inpatient	Access	240	408
		Watch	120	
		Reserve	0	
Xu JJ et al., 2020 [34]	Inpatient	Access	487	3975
		Watch	2743	
		Reserve	18	
Olaru ID et al., 2020 [35]	Inpatient	Access	144	264
		Watch	91	
		Reserve	0	
Wang CN et al., 2020 [36]	Outpatient	Access	9982	102412
		Watch	84176	
		Reserve	17	
Zhang M et al., 2020 [37]	Inpatient	Access	911	4795
		Watch	3884	
		Reserve	0	
Nair V et al., 2015 [38]	Inpatient	Access	414	1940
		Watch	938	
		Reserve	0	

Study	Medical setting	The AWaRe group	The number of prescriptions including antibiotics in the Access, Watch, or Reserve group	Total number of prescriptions
Labi AK et al., 2018 [39]	Inpatient	Access	272	611
		Watch	338	
		Reserve	0	
Shrestha B et al., 2018 [40]	Inpatient	Access	197	641
		Watch	347	
		Reserve	0	
Chaw PS et al., 2018 [41]	Inpatient	Access	564	917
		Watch	146	
		Reserve	0	
Kebede HK et al., 2017 [42]	Inpatient	Access	720	812
		Watch	50	
		Reserve	0	
Goycochea-Valdivia WA et al., 2017 [43]	Inpatient	Access	49	118
		Watch	67	
		Reserve	2	
Sisay M et al., 2017 [44]	Outpatient	Access	676	935
		Watch	245	
		Reserve	0	
Cai Y et al., 2017 [45]	Inpatient	Access	1448	3611
		Watch	1298	
		Reserve	0	
Stefkovicova M et al., 2016 [46]	Inpatient	Access	1019	3205
		Watch	1465	
		Reserve	0	
Kiguba R et al., 2016 [47]	Inpatient	Access	281	866
		Watch	585	
		Reserve	0	
Bilal AI et al., 2016 [48]	Outpatient	Access	526	636
		Watch	103	
		Reserve	0	
Atif M et al., 2017 [49]	Inpatient	Access	208	1143
		Watch	935	
		Reserve	0	
Nsofor CA et al., 2016 [50]	Inpatient	Access	463	533
		Watch	70	
		Reserve	0	
Jose MW et al., 2016 [51]	Inpatient	Access	427	667
		Watch	198	
		Reserve	0	
Assen A et al., 2014 [52]	Outpatient	Access	148	221

Study	Medical setting	The AWaRe group	The number of prescriptions including antibiotics in the Access, Watch, or Reserve group	Total number of prescriptions
		Watch	54	
		Reserve	0	
Desalegn AA et al., 2013 [53]	Outpatient	Access	714	841
		Watch	127	
		Reserve	0	
Borras Novell C et al., 2013 [54]	Inpatient	Access	248	467
		Watch	219	
		Reserve	0	
Dryden M et al., 2012 [55]	Inpatient	Access	335	556
		Watch	194	
		Reserve	7	
Okoro RN et al., 2019 [56]	Outpatient	Access	131	275
		Watch	127	
		Reserve	0	
Arnoldo L et al., 2019 [57]	Inpatient	Access	528	4009
		Watch	2319	
		Reserve	0	
Gwebu P et al., 2022 [58]	Inpatient	Access	72	103
		Watch	30	
		Reserve	0	
Talaat M et al., 2022 [59]	Inpatient	Access	5470	16071
		Watch	10308	
		Reserve	293	
Zhang J et al., 2022 [60]	Inpatient	Access	552	3680
		Watch	2814	
		Reserve	58	
Shaikh Q et al., 2022 [61]	Inpatient	Access	85	274
		Watch	176	
		Reserve	4	
Akkawi ME et al., 2022 [62]	Inpatient	Access	159	385
		Watch	224	
		Reserve	0	
Nguyen HQ et al., 2022 [63]	Inpatient	Access	7539	34719
		Watch	27128	
		Reserve	52	
Sharif M et al., 2022 [64]	Inpatient	Access	217	1054
		Watch	586	
		Reserve	0	
Limato R et al., 2021 [65]	Inpatient	Access	356	1273
		Watch	858	

Study	Medical setting	The AWaRe group	The number of prescriptions including antibiotics in the Access, Watch, or Reserve group	Total number of prescriptions
		Reserve	31	
Ishibashi N et al., 2022 [66]	Inpatient	Access	49	137
		Watch	80	
		Reserve	3	
Ashour RH et al., 2022 [67]	Inpatient	Access	156	300
		Watch	129	
		Reserve	15	
Panditrao AM et al., 2021 [68]	Inpatient	Access	1077	2834
		Watch	1641	
		Reserve	116	
Rashid MM et al., 2022 [69]	Inpatient	Access	752	2112
		Watch	1352	
		Reserve	2	
D'Arcy N et al., 2021 [70]	Inpatient	Access	1892	3298
		Watch	1335	
		Reserve	0	
Kiggundu R et al., 2022 [71]	Inpatient	Access	654	1387
		Watch	612	
		Reserve	0	
Wang CN et al., 2021 [72]	Inpatient	Access	2664	15845
		Watch	12915	
		Reserve	266	
Rocke T et al., 2022 [73]	Inpatient	Access	238	376
		Watch	138	
		Reserve	0	
El-Dahiyat F et al., 2022 [74]	Outpatient	Access	104	419
		Watch	315	
		Reserve	0	
Tadesse TY et al., 2022 [75]	Inpatient	Access	384	1444
		Watch	1060	
		Reserve	0	
Amponsah OKO et al., 2022 [76]	Outpatient	Access	28152	58841
		Watch	27395	
		Reserve	1045	
Abu Farha R et al., 2022 [77]	Outpatient	Access	68	207
		Watch	139	
		Reserve	0	
Anugulruengkitt S et al., 2022 [78]	Inpatient	Access	3111	6619
		Watch	3131	
		Reserve	377	

Study	Medical setting	The AWaRe group	The number of prescriptions including antibiotics in the Access, Watch, or Reserve group	Total number of prescriptions
Chansamouth V et al., 2022 [79]	Inpatient	Access	3244	6433
		Watch	3156	
		Reserve	0	

References for included studies

1. Levy HG, Rojas-Cortes R, Molina LH, Dreser MA, Alfonso OI, Rizo-Amezquita JN, et al. Point prevalence survey of antibiotic use in hospitals in Latin American countries. J Antimicrob Chemoth. 2022;77(3):807-15.
2. Moulin E, Boillat-Blanco N, Zanetti G, Plüss-Suard C, De Vallière S, Senn L. Point prevalence study of antibiotic appropriateness and possibility of early discharge from hospital among patients treated with antibiotics in a Swiss university hospital. Antimicrobial Resistance & Infection Control. 2022;11(1):1-8.
3. Magill SS, O'Leary E, Ray SM, Kainer MA, Evans C, Bamberg WM, et al. Antimicrobial use in US hospitals: comparison of results from emerging infections program prevalence surveys, 2015 and 2011. Clin Infect Dis. 2021;72(10):1784-92.
4. Mustafa ZU, Salman M, Yasir M, Godman B, Majeed HA, Kanwal M, et al. Antibiotic consumption among hospitalized neonates and children in Punjab province, Pakistan. Expert Rev Anti-Infe. 2022;20(6):931-9.
5. Yi S, Ramachandran A, Epps L, Mayah A, Burkholder TW, Jaung MS, et al. Emergency department antimicrobial use in a low-resource setting: results from a retrospective observational study at a referral hospital in Liberia. Bmj Open. 2022;12(4):e56709.
6. Haseeb A, Faidah HS, Algethamy M, Alghamdi S, Alhazmi GA, Alshomrani AO, et al. Antimicrobial usage and resistance in Makkah region hospitals: a regional point prevalence survey of public hospitals. Int J Env Res Pub He. 2021;19(1):254.
7. Ogunleye OO, Oyawole MR, Odunuga PT, Kalejaye F, Yinka-Ogunleye AF, Olalekan A, et al. A multicentre point prevalence study of antibiotics utilization in hospitalized patients in an urban secondary and a tertiary healthcare facilities in Nigeria: findings and implications. Expert Rev Anti-Infe. 2022;20(2):297-306.
8. Boone K, Morris SK, Doshi S, Black J, Mohsin M, Ahmed T, et al. Antimicrobial prescribing during infant hospital admissions in a birth cohort in Dhaka, Bangladesh. J Trop Pediatrics. 2021;67(3):a93.
9. Kurdi A, Hasan AJ, Baker KI, Seaton RA, Ramzi ZS, Sneddon J, et al. A multicentre point prevalence survey of hospital antibiotic prescribing and quality indices in the Kurdistan regional government of Northern Iraq: the need for urgent action. Expert Rev Anti-Infe. 2021;19(6):805-14.
10. Skosana PP, Schellack N, Godman B, Kurdi A, Bennie M, Kruger D, et al. A point prevalence survey of antimicrobial utilisation patterns and quality indices amongst hospitals in South Africa; findings and implications. Expert Rev Anti-Infe. 2021;19(10):1353-66.
11. Blackburn J, Barrowman N, Bowes J, Tsampalieros A, Le Saux N. Establishing benchmarks for antimicrobial use in Canadian children's hospitals: results from 2 national point prevalence surveys. Pediatr Infect Dis J. 2021;40(10):899-905.

12. Pauwels I, Versporten A, Drapier N, Vlieghe E, Goossens H. Hospital antibiotic prescribing patterns in adult patients according to the WHO Access, Watch and Reserve classification (AWaRe): results from a worldwide point prevalence survey in 69 countries. J Antimicrob Chemoth. 2021;76(6):1614-24.
13. Tassew SG, Abraha HN, Gidey K, Gebre AK. Assessment of drug use pattern using WHO core drug use indicators in selected general hospitals: a cross-sectional study in Tigray region, Ethiopia. Bmj Open. 2021;11(10):e45805.
14. Yehualaw A, Taferre C, Bantie AT, Demsie DG. Appropriateness and pattern of antibiotic prescription in pediatric patients at Adigart General Hospital, Tigray, Ethiopia. Biomed Res Int. 2021;2021:6640892.
15. Wendie TF, Ahmed A, Mohammed SA. Drug use pattern using WHO core drug use indicators in public health centers of Dessie, North-East Ethiopia. Bmc Med Inform Decis. 2021;21(1):197.
16. de Guzman BG, Pauwels I, Versporten A, Goossens H, De Los RM, Gler MT. Implementation of a multidisciplinary antimicrobial stewardship programme in a Philippine tertiary care hospital: an evaluation by repeated point prevalence surveys. J Glob Antimicrob Re. 2021;26:157-65.
17. Momanyi L, Opanga S, Nyamu D, Oluka M, Kurdi A, Godman B. Antibiotic prescribing patterns at a leading referral hospital in Kenya: a point prevalence survey. J Res Pharm Pract. 2019;8(3):149-54.
18. Soltani J, Pouladfar G, Versporten A, Sharland M, Soleimani N. Point prevalence survey of antimicrobial prescription and infection in pediatric and neonatal wards of two Iranian teaching hospitals. 2019;41(1):25-32.
19. Saleem Z, Hassali MA, Versporten A, Godman B, Hashmi FK, Goossens H, et al. A multicenter point prevalence survey of antibiotic use in Punjab, Pakistan: findings and implications. Expert Rev Anti-Infe. 2019;17(4):285-93.
20. Al MM, Enani M, Binsaleh G, Roushdy H, Alokaili D, Al BA, et al. Point prevalence survey of antibiotic use in 26 Saudi hospitals in 2016. J Infect Public Heal. 2019;12(1):77-82.
21. Komagamine J, Yabuki T, Hiraiwa T. A trend in prevalence of antimicrobial use and appropriateness of antimicrobial therapy in an acute care hospital from 2018 to 2019: repeated prevalence surveys in Japan. Bmc Res Notes. 2019;12(1):811.
22. Yimenu DK, Emam A, Elemineh E, Atalay W. Assessment of antibiotic prescribing patterns at outpatient pharmacy using World Health Organization prescribing indicators. J Prim Care Communit. 2019;10:922934690.
23. Komagamine J, Yabuki T, Kobayashi M, Okabe T. Prevalence of antimicrobial use and active healthcare-associated infections in acute care hospitals: a multicentre prevalence survey in Japan. Bmj Open. 2019;9(6):e27604.
24. Komagamine J, Kobayashi M, Mori T. Prevalence of and rationale for antimicrobial prescription during ambulatory care visits in Japan: a prospective, multicentre, cross-sectional study. Bmj Open. 2020;10(8):e39329.
25. Masich AM, Vega AD, Callahan P, Herbert A, Fwoloshi S, Zulu PM, et al. Antimicrobial usage at a large teaching hospital in Lusaka, Zambia. Plos One. 2020;15(2):e228555.
26. Khan Z, Ahmed N, Zafar S, Ullah KF, Ur RA, Parreiras MM, et al. A pilot study on the rational use of medicines in four tertiary care hospitals through validated World Health Organization prescribing drugs indicators. Ann Ig Med Prev Comu. 2020;32(4):368-75.
27. Yilma Z, Mekonnen T, Siraj EA, Agmassie Z, Yehualaw A, Debasu Z, et al. Assessment of prescription completeness and drug use pattern in Tibebe-Ghion Comprehensive Specialized Hospital,

Bahir Dar, Ethiopia. Biomed Res Int. 2020;2020:8842515.
28. Seni J, Mapunjo SG, Wittenauer R, Valimba R, Stergachis A, Werth BJ, et al. Antimicrobial use across six referral hospitals in Tanzania: a point prevalence survey. Bmj Open. 2020;10(12):e42819.
29. Abubakar U. Antibiotic use among hospitalized patients in northern Nigeria: a multicenter point-prevalence survey. Bmc Infect Dis. 2020;20(1):86.
30. Horumpende PG, Mshana SE, Mouw EF, Mmbaga BT, Chilongola JO, de Mast Q. Point prevalence survey of antimicrobial use in three hospitals in North-Eastern Tanzania. Antimicrob Resist in. 2020;9(1):149.
31. Mohammed ZA, Mukhopadhyay C, Varma M, Kalwaje EV. Identifying opportunities for antimicrobial stewardship through a point prevalence survey in an Indian tertiary-care teaching hospital. J Glob Antimicrob Re. 2020;23:315-20.
32. Loftus MJ, Curtis SJ, Naidu R, Cheng AC, Jenney A, Mitchell BG, et al. Prevalence of healthcare-associated infections and antimicrobial use among inpatients in a tertiary hospital in Fiji: a point prevalence survey. Antimicrob Resist in. 2020;9(1):146.
33. Gardiner SJ, Basevi AB, Hamilton NL, Metcalf SC, Chambers ST, Withington SG, et al. Point prevalence surveys of antimicrobial use in adult inpatients at Canterbury District Health Board Hospitals. New Zeal Med J. 2020;133(1525):18-33.
34. Xu JJ, Gao J, Guo JH, Song LL. Analysis of antibiotic treatment of children in a Shanghai tertiary hospital based on point prevalence surveys. Bmc Infect Dis. 2020;20(1):804.
35. Olaru ID, Meierkord A, Godman B, Ngwenya C, Fitzgerald F, Dondo V, et al. Assessment of antimicrobial use and prescribing practices among pediatric inpatients in Zimbabwe. J Chemotherapy. 2020;32(8):456-9.
36. Wang CN, Huttner BD, Magrini N, Cheng Y, Tong J, Li S, et al. Pediatric antibiotic prescribing in China according to the 2019 World Health Organization Access, Watch, and Reserve (AWaRe) antibiotic categories. J Pediatr-Us. 2020;220:125-31.
37. Zhang M, Ma XY, Feng ZQ, Gao L. Survey of antibiotic use among hospitalised children in a hospital in Northeast China over a 4-year period. J Spec Pediatr Nurs. 2020;25(2):e12282.
38. Nair V, Sharma D, Sahni AK, Grover N, Shankar S, Jaiswal SS, et al. Antimicrobial use and antimicrobial resistance in nosocomial pathogens at a tertiary care hospital in Pune. Med J Armed Forces India. 2015;71(2):112-9.
39. Labi AK, Obeng-Nkrumah N, Nartey ET, Bjerrum S, Adu-Aryee NA, Ofori-Adjei YA, et al. Antibiotic use in a tertiary healthcare facility in Ghana: a point prevalence survey. Antimicrob Resist in. 2018;7:15.
40. Shrestha B, Dixit SM. The assessment of drug use pattern using WHO prescribing indicators. J Nepal Health Res Counc. 2018;16(3):279-84.
41. Chaw PS, Schlinkmann KM, Raupach-Rosin H, Karch A, Pletz MW, Huebner J, et al. Antibiotic use on paediatric inpatients in a teaching hospital in the Gambia, a retrospective study. Antimicrob Resist in. 2018;7:82.
42. Kebede HK, Gesesew HA, Woldehaimanot TE, Goro KK. Antimicrobial use in paediatric patients in a teaching hospital in Ethiopia. Plos One. 2017;12(3):e173290.
43. Goycochea-Valdivia WA, Moreno-Ramos F, Pano-Pardo JR, Aracil-Santos FJ, Baquero-Artigao F, Del RT, et al. Identifying priorities to improve paediatric in-hospital antimicrobial use by cross-sectional evaluation of prevalence and appropriateness of prescription. Enferm Infec Micr Cl. 2017;35(9):556-62.

44. Sisay M, Mengistu G, Molla B, Amare F, Gabriel T. Evaluation of rational drug use based on World Health Organization core drug use indicators in selected public hospitals of eastern Ethiopia: a cross sectional study. Bmc Health Serv Res. 2017;17(1):161.
45. Cai Y, Venkatachalam I, Tee NW, Tan TY, Kurup A, Wong SY, et al. Prevalence of healthcare-associated infections and antimicrobial use among adult inpatients in Singapore acute-care hospitals: results from the first national point prevalence survey. Clin Infect Dis. 2017;64(suppl_2):S61-7.
46. Stefkovicova M, Litvova S, Melus V, Kristufkova Z, Brazinova A. Point prevalence study of antimicrobial usage in acute care hospitals in the Slovak Republic. J Hosp Infect. 2016;93(4):403-9.
47. Kiguba R, Karamagi C, Bird SM. Extensive antibiotic prescription rate among hospitalized patients in Uganda: but with frequent missed-dose days. J Antimicrob Chemoth. 2016;71(6):1697-706.
48. Bilal AI, Osman ED, Mulugeta A. Assessment of medicines use pattern using World Health Organization's prescribing, patient care and health facility indicators in selected health facilities in eastern Ethiopia. Bmc Health Serv Res. 2016;16:144.
49. Atif M, Azeem M, Saqib A, Scahill S. Investigation of antimicrobial use at a tertiary care hospital in southern Punjab, Pakistan using WHO methodology. Antimicrob Resist in. 2017;6:41.
50. Nsofor CA, Amadi ES, Obijuru CE, Ohalete CV, Ukwandu N. Prevalence of antimicrobial use in major hospitals in Owerri, Nigeria. EC Microbiology. 2016;3(5):522-7.
51. José MW, Jean-Marie LI, Divine MM, Sabine KK, Takaisi- K. Point prevalence study of antibiotic use in hospitals in Butembo. International Journal of Medicine and Medical Sciences. 2016;8(12):133-9.
52. Assen A, Abrha S. Assessment of drug prescribing pattern in Dessie Referral Hospital, Dessie. International Journal of Pharma Sciences & Research. 2014;5(11):777-81.
53. Desalegn AA. Assessment of drug use pattern using WHO prescribing indicators at Hawassa University Teaching and Referral Hospital, south Ethiopia: a cross-sectional study. Bmc Health Serv Res. 2013;13(1):170.
54. Borras NC, Hernandez BS, Garcia GJ. Prescribing of antibiotics in patients admitted from emergency departments: a multicenter study. An Pediatr. 2013;79(1):15-20.
55. Dryden M, Saeed K, Townsend R, Winnard C, Bourne S, Parker N, et al. Antibiotic stewardship and early discharge from hospital: impact of a structured approach to antimicrobial management. J Antimicrob Chemoth. 2012;67(9):2289-96.
56. Okoro RN, Nmeka C, Erah PO. Antibiotics prescription pattern and determinants of utilization in the national health insurance scheme at a tertiary hospital in Nigeria. Afr Health Sci. 2019;19(3):2356-64.
57. Arnoldo L, Smaniotto C, Celotto D, Brunelli L, Cocconi R, Tignonsini D, et al. Monitoring healthcare-associated infections and antimicrobial use at regional level through repeated point prevalence surveys: what can be learnt? J Hosp Infect. 2019;101(4):447-54.
58. Gwebu P, Meyer J, Schellack N, Matsebula-Myeni Z, Godman B. A web-based point prevalence survey of antimicrobial use and quality indicators at Raleigh Fitkin Memorial Hospital in Eswatini and the implications. Pharmacoepidem Dr S. 2022;50(6):214-21.
59. Talaat M, Tolba S, Abdou E, Sarhan M, Gomaa M, Hutin YJ. Over-prescription and overuse of antimicrobials in the Eastern Mediterranean Region: the urgent need for Antimicrobial Stewardship Programs with Access, Watch, and Reserve adoption. Antibiotics-Basel. 2022;11(12):1773.
60. Zhang J, Zhang W, Ma X, Tang L, Tian D, Wu K, et al. Antimicrobial prescribing for children in

China: data from point prevalence surveys in 18 tertiary centres in China in 2016-2017. Bmj Open. 2022;12(9).

61. Shaikh Q, Sarfaraz S, Rahim A, Hussain A, Behram S, Kazi AS, et al. WHO point prevalence survey to describe the use of antimicrobials at a tertiary care center in Pakistan: a situation analysis for establishing an antimicrobial stewardship program. Antibiotics-Basel. 2022;11(11):1555.

62. Akkawi ME, Taffour RM, AL-Shami AM. Evaluation of antibiotic prescribing pattern and appropriateness among hospitalized pediatric patients: findings from a Malaysian teaching hospital. Infect Dis Rep. 2022;14(6):889-99.

63. Nguyen HQ, Nguyen-Thi HY, Huynh PT, Le NDT, Nguyen NT, Hsia Y. Effectiveness of an enhanced antibiotic stewardship programme among paediatric patients in a tertiary hospital in Vietnam. J Hosp Infect. 2022;127:121-8.

64. Sharif M, Aslam S, Saleem Z. Point prevalence survey to estimate antimicrobial use in a tertiary care university hospital in Pakistan using WHO methodology: findings and implications. Infect Dis-Nor. 2022;54(9):698-701.

65. Limato R, Nelwan EJ, Mudia M, de Brabander J, Guterres H, Enty E, et al. A multicentre point prevalence survey of patterns and quality of antibiotic prescribing in Indonesian hospitals. Jac-Antimicrob Resis. 2021;3(2):b47.

66. Ishibashi N, Pauwels I, Tomori Y, Gu Y, Yamaguchi T, Handa T, et al. Point prevalence surveys of antimicrobial prescribing in a non-acute care hospital in Saitama Prefecture, Japan. Can J Infect Dis Med. 2022;2022:2497869.

67. Ashour RH, Abdelkader EA, Hamdy O, Elmetwally M, Laimon W, Abd-Elaziz MA. The pattern of antimicrobial prescription at a tertiary health center in Egypt: a point survey and implications. Infect Drug Resist. 2022;15:6365-78.

68. Panditrao AM, Shafiq N, Chatterjee S, Pathak A, Trivedi N, Sadasivam B, et al. A multicentre point prevalence survey (PPS) of antimicrobial use amongst admitted patients in tertiary care centres in India. J Antimicrob Chemoth. 2021;76(4):1094-101.

69. Rashid MM, Akhtar Z, Chowdhury S, Islam MA, Parveen S, Ghosh PK, et al. Pattern of antibiotic use among hospitalized patients according to WHO Access, Watch, Reserve (AWaRe) classification: findings from a point prevalence survey in Bangladesh. Antibiotics-Basel. 2022;11(6).

70. D'Arcy N, Ashiru-Oredope D, Olaoye O, Afriyie D, Akello Z, Ankrah D, et al. Antibiotic prescribing patterns in Ghana, Uganda, Zambia and Tanzania hospitals: results from the Global Point Prevalence Survey (G-PPS) on antimicrobial use and stewardship interventions implemented. Antibiotics-Basel. 2021;10(9):1122.

71. Kiggundu R, Wittenauer R, Waswa JP, Nakambale HN, Kitutu FE, Murungi M, et al. Point prevalence survey of antibiotic use across 13 hospitals in Uganda. Antibiotics-Basel. 2022;11(2):199.

72. Wang CN, Tong J, Yi B, Huttner BD, Cheng Y, Li S, et al. Antibiotic use among hospitalized children and neonates in China: results from quarterly point prevalence surveys in 2019. Front Pharmacol. 2021;12:601561.

73. Rocke T, El ON, Quiros RE, Hsieh J, Ramon-Pardo P. Reporting on antibiotic use patterns using the WHO Access, Watch, Reserve classification in the Caribbean. Rev Panam Salud Publ. 2022;46:e186.

74. El-Dahiyat F, Salah D, Alomari M, Elrefae A, Jairoun AA. Antibiotic prescribing patterns for outpatient pediatrics at a private hospital in Abu Dhabi: a clinical audit study. Antibiotics-Basel. 2022;11(12):1676.

75. Tadesse TY, Molla M, Yimer YS, Tarekegn BS, Kefale B. Evaluation of antibiotic prescribing patterns among inpatients using World Health Organization indicators: a cross-sectional study. Sage Open Med. 2022;10(2):1-10.
76. Amponsah OKO, Nagaraja SB, Ayisi-Boateng NK, Nair D, Muradyan K, Asense PS, et al. High levels of outpatient antibiotic prescription at a district hospital in Ghana: results of a cross sectional study. Int J Env Res Pub He. 2022;19(16):10286.
77. Abu Farha R, Awwad O, Abdurazaq B, Abu Hammour K, Akour A. Evaluation of drug use pattern in adults' outpatient clinics in a tertiary teaching hospital using WHO core prescribing indicators. J Pharm Health Serv. 2022;13(4):357-63.
78. Anugulruengkitt S, Charoenpong L, Kulthanmanusorn A, Thienthong V, Usayaporn S, Kaewkhankhaeng W, et al. Point prevalence survey of antibiotic use among hospitalized patients across 41 hospitals in Thailand. Jac-Antimicrob Resis. 2022;5(1):c140.
79. Chansamouth V, Chommanam D, Roberts T, Keomany S, Paphasiri V, Phamisith C, et al. Evaluation of trends in hospital antimicrobial use in the Lao PDR using repeated point-prevalence surveys-evidence to improve treatment guideline use. Lancet Reg Health-W. 2022;27:100531.

Supplementary Table 9. Other indicators of hospital antibiotic prescribing

Table S9-1. Other indicators of antibiotic prescribing in outpatient settings

	**Reports (n)**	**Percentage (%)**	**95% CI**	**I^2 (%)**
The percentage of antibiotics prescribed for combination	24	14.7	11.4–18.2	99.0
The percentage of antibiotics administered parenterally	12	17.4	7.0–31.2	99.7
The percentage of inappropriate antibiotic prescribing	14	26.1	13.8–40.7	>99.9
The condition of antibiotic prescribing based on AWaRe classification				
Access	14	48.5	34.5–62.7	>99.9
Watch	14	43.1	30.4–56.3	>99.9
Reserve	14	<0.1	NA	99.4

NA - Not Applicant.

Table S9-2. Other indicators of antibiotic prescribing in inpatient settings

	**Reports (n)**	**Percentage (%)**	**95% CI**	**I^2 (%)**
The percentage of antibiotics prescribed for combination	149	38.4	35.6–41.3	99.4
The percentage of empiric antibiotics	56	73.7	68.0–79.1	99.4
The percentage of antibiotics prescribed for prophylaxis	120	32.1	29.6–34.8	99.4
The percentage of antibiotics administered parenterally	114	76.2	73.4–78.8	99.4
The percentage of inappropriate antibiotic prescribing	53	37.8	33.8–41.9	97.9
The condition of antibiotic prescribing based on AWaRe classification				
Access	65	43.8	39.2–48.5	99.7
Watch	65	47.9	43.0–52.8	99.7
Reserve	65	0.4	0.2–0.6	98.3

Supplementary Table 10. Factors associated with hospital antibiotic prescribing

Table S10. Factors associated with hospital antibiotic prescribing

Study	Original description	Summary of relevant factors	Description of associated factors	OR (95% CI)
Ciofi Degli Atti ML et al., 2019 [1]	Sex	Gender of the patient	Male: Female	1.1 (1.0–1.3)
	Age	Age of the patient	3–11 months: ≤2 months	1.0 (0.8–1.3)
			1–5 years: ≤2 months	1.5 (1.2–1.9)
			6–11 years: ≤2 months	1.7 (1.4–2.2)
			≥12 years: ≤2 months	1.6 (1.3–2.1)
	Length of hospital stay (days)	Hospitalization duration	8–30: ≤7	1.4 (1.2–1.7)
			≥30: ≤7	1.0 (0.8–1.2)
Denny KJ et al., 2019 [2]	Age, years	Age of the patient	≥18: 43/188; <18: 290/785	
	Gender	Gender of the patient	Male: 175/499; Female: 158/474	
Desai NM et al., 2020 [3]	Presence of fever	Symptom (fever or not)	Yes: No	1.04 (0.98–1.10)
	Chest radiography performed	Performing chest radiography or not	Yes: No	1.88 (1.73–2.04)
	Past history of asthma	Asthma history	Yes: No	0.88 (0.79–0.97)
Anteneh DA et al., 2021 [4]	Patient gender	Gender of the patient	Male: Female	3.32 (1.39–7.89)
	Patient age category	Age of the patient	18–34 years: Above 50 years	0.78 (0.28–2.16)
			35–50 years: Above 50 years	1.24 (0.47–3.28)
	Residency	Settings	Rural: Urban	0.65 (0.29–1.46)
	Educational level	Educational level of the patient	Not read or write: High school and above	0.22 (0.07–0.67)
			Elementary school: High school and above	0.35 (0.14–0.88)
	Health assurance	Health insurance	Free services: Direct payment	3.42 (1.44–8.13)
	Prescriber gender	Gender of the physician	Male: Female	1.34 (0.56–3.22)
	Prescriber age	Age of the physician	20–34 years: 35–44 years	1.11 (0.36–3.40)

Study	Original description	Summary of relevant factors	Description of associated factors	OR (95% CI)
	Prescriber experience	Practice duration of the physician	Practionar (<1 y): Professional (≥1 y)	3.63 (1.53–8.64)
Boone K et al., 2021 [5]	Sex	Gender of the patient	Male: Female	0.89 (0.77–1.04)
	Age	Age of the patient	29 days to 3 months of age: 0 to ≤28 days	0.93 (0.73–1.17)
			3–12 months of age: 0 to ≤28 days	0.85 (0.73–0.99)
	Facility type	Facility type	Government: Private	0.70 (0.60–0.82)
			Charitable: Private	0.39 (0.29–0.53)
Akkawi ME et al., 2022 [6]	Gender	Gender of the patient	Male: Female	1.005 (0.598–1.687)
	Age categories	Age of the patient	1–3 years: <1 year	0.705 (0.343–1.450)
			3–6 years: <1 year	0.449 (0.199–1.015)
			>6 years: <1 year	0.619 (0.268–1.432)
	Days of hospitalization	Hospitalization duration	Longer: Shorter	1.041 (0.903–1.199)
Cotter JM et al., 2022 [7]	Age in years	Age of the patient	Older: Younger	1.01 (1.00 – 1.02)
	History of fever at home	Symptom (fever or not)	Yes: No	1.66 (1.22–2.27)
	Days of illness	Illness duration	Three or more days: less than three days	1.05 (0.91–1.22)
	History of asthma	Asthma history	Yes: No	0.98 (0.85–1.13)
Dilworth TJ et al., 2022 [8]	Patient gender	Gender of the patient	Male: Female	1.10 (1.06–1.14)
	Patient age	Age of the patient	Older: Younger	1.02 (1.02–1.02)
Kitt E et al., 2022 [9]	Age category (months)	Age of the patient	≤6: >6	0.790 (0.460–1.356)
	Comorbidity	Comorbidity status	Yes: No	0.869 (0.631–1.264)
	Sex	Gender of the patient	Female: Male	0.902 (0.631–1.288)
Kroening-Roche JC et al., 2012 [10]	Gender	Gender of the patient	Male*Antibiotic: 280<br>Male*No antibiotic: 106<br>Female*Antibiotic: 341<br>Female*No antibiotic: 108	

Study	Original description	Summary of relevant factors	Description of associated factors	OR (95% CI)
	Age	Age of the patient	≥ 50 years*Antibiotic: 270<br>≥ 50 years*No antibiotic: 67<br>< 50 years*Antibiotic: 352<br>< 50 years*No antibiotic: 147	
	With alcohol abuse or not	The patient is with alcohol abuse or not	Yes*Antibiotic: 64<br>Yes*No antibiotic: 23<br>No*Antibiotic: 276<br>No*No antibiotic: 84	
Linder JA et al., 2003 [11]	Wanting antibiotics or not	Patient demand	Yes: No	2.1 (1.1–4.4)
	The result of the lung exam	The result of the lung exam	Abnormal: Normal	11.9 (4.0–35.6)
	Temperature	Symptom (fever or not)	Yes: No	2.0 (1.3–3.0)
Kornblith AE et al., 2018 [12]	Age category (years)	Age of the patient	1–5: < 1	1.26 (1.07–1.48)
			6–10: < 1	1.18 (0.93–1.50)
			11–18: < 1	1.17 (0.97–1.51)
	Sex	Gender of the patient	Male: Female	0.96 (0.83–1.11)
Kozyrskyj AL et al., 2004 [13]	Age, yr (physician)	Age of the physician	≥50: <50	1,21 (1.07–1.38)
	Season of visit	Season of visit	Spring: Winter	0.88 (0.83–0.92)
			Summer: Winter	0.88 (0.84–0.94)
			Fall: Winter	0.95 (0.90–1.00)
	Patient age	Age of the patient	Older: Younger	1.02 (1.02–1.03)
	Patient sex	Gender of the patient	Male: Female	1.06 (1.02–1,10)
	Annual household income	Income level of the patient	Higher: Lower	0.99 (0.98–0.99)

Study	Original description	Summary of relevant factors	Description of associated factors	OR (95% CI)
Curt AM et al., 2020 [14]	Patient age	Age of the patient	2–11.9 months*Antibiotic: 48<br>2–11.9 months*No antibiotic: 76<br>1–4.9 years*Antibiotic: 182<br>1–4.9 years*No antibiotic: 313<br>5–18 years*Antibiotic: 103<br>5–18 years*No antibiotic: 164	
	Patient sex	Gender of the patient	Male*Antibiotic: 172<br>Male*No antibiotic: 106<br>Female*Antibiotic: 304<br>Female*No antibiotic: 249	
Kumar R et al., 2008 [15]	Settings	Settings	Urban: 957/1029<br>Rural: 1039/1231	
	Facility type	Facility type	Government: 976/1234<br>Private: 1020/1206	
	Fever	Symptom (fever or not)	Yes: 1833<br>Yes*Antibiotic: 1833*0.835<br>No: 607<br>No*Antibiotic: 607*0.779	
	Cough	Symptom (cough or not)	Yes: 1906<br>Yes*Antibiotic: 1906*0.823<br>No: 534<br>No*Antibiotic: 534*0.801	

Study	Original description	Summary of relevant factors	Description of associated factors	OR (95% CI)
	Comorbidity	Comorbidity status	Yes: 9<br>Yes*Antibiotic: 9*0.777<br>No: 2431<br>No*Antibiotic: 2431*0.818	
Cantrell R et al., 2002 [16]	Age	Age of the patient	45–64 y: 18–44 y	0.576 (0.350–0.940)
			>64 y: 18–44 y	0.420 (0.250–0.710)
	Sex	Gender of the patient	Female: Male	0.845 (0.560–1.270)
Arnold SR et al., 2005 [17]	Patient age	Age of the patient	<2 y: 231/1204<br>>2 y: 308/1987	
	Patient temperature	Symptom (fever or not)	>38.5 °C: 350/2003<br><38.5 °C: 189/1987	
	Gender	Gender of the patient	Male: 292/2245<br>Female: 319/1645	
	Duration of time in practice	Practice duration of the physician	0–5 y: 47/471<br>6–10 y: 92/730<br>11–15 y: 103/832<br>16–20 y: 68/518<br>>20 y: 202/1339	
	Age of physician	Age of the physician	Older: Younger	1.17 (1.11–1.24)
Cadieux G et al., 2011 [18]	Patient gender	Gender of the patient	Male: Female	1.00 (0.96–1.03)
	Patient age category (yr)	Age of the patient	4–18: 1–3	1.11 (1.03–1.19)
			19–40: 1–3	1.06 (0.94–1.19)
			40–64: 1–3	1.20 (1.06–1.36)
			>65: 1–3	1.16 (1.01–1.34)
	Patient geographical area	Settings	Rural: Urban	0.75 (0.49–1.12)

Study	Original description	Summary of relevant factors	Description of associated factors	OR (95% CI)
	Patient education	Educational level of the patient	11–17: <10	0.95 (0.90–1.00)
			18–29: <10	0.90 (0.83–0.98)
			>30: <10	0.83 (0.73–0.94)
	Patient household income	Income level of the patient	33296–41983: 0–33295	1.05 (0.98–1.11)
			41984–42080: 0–33295	1.11 (1.03–1.20)
			≥52081: 0–33295	0.67 (0.31–1.45)
	Comorbidity	Comorbidity status	Yes: No	1.03 (1.01–1.05)
Barnett ML et al., 2014 [19]	Age, y	Age of the patient	45–64: 18–44	0.99 (0.64–1.53)
	Gender	Gender of the patient	Male: Female	0.95 (0.64–1.40)
	Rural/Urban	Settings	Urban: Rural	1.23 (0.61–2.49)
Bergmark RW et al., 2016 [20]	Age (years)	Age of the patient	Older: Younger	1.01 (1.00–1.02)
	Gender	Gender of the patient	Female: Male	1.39 (0.83–2.31)
	Temperature	Symptom (fever or not)	Yes: No	0.82 (0.45–1.49)
Depew RE et al., 2020 [21]	Age, years	Age of the patient	35–49: 18–34	0.89 (0.80–0.99)
			50–64: 18–34	0.84 (0.74–0.99)
			65+: 18–34	0.66 (0.57–0.77)
	Sex	Gender of the patient	Male: Female	1.02 (0.95–1.13)
	Comorbid conditions	Comorbidity status	1 comorbidity: No comorbid conditions	1.25 (1.13–1.38)
			>1 comorbidity: No comorbid conditions	1.50 (1.25–1.80)
Coco AS et al., 2009 [22]	Sex	Gender of the patient	Male: Female	1.02 (0.92–1.12)
	Age	Age of the patient	≥ 2 years: < 2 years	1.05 (0.94–1.18)
	Fever	Symptom (fever or not)	Yes: No	0.96 (0.86–1.07)
Copp HL et al., 2011 [23]	Age, y	Age of the patient	<2: 13–17	6.4 (2.2–18.7)
			2–5: 13–17	1.6 (0.5–4.5)

Study	Original description	Summary of relevant factors	Description of associated factors	OR (95% CI)
			6–12: 13–17	1.4 (0.5–3.7)
	Gender	Gender of the patient	Female: Male	3.6 (1.6–8.5)
	Temperature	Symptom (fever or not)	Yes: No	2.9 (1.0–8.6)
Ababneh MA et al., 2017 [24]	Age (years)	Age of the patient	0–5: 6–12	1.52 (1.34–1.73)
			13–17: 6–12	1.46 (0.76–2.73)
	Gender	Gender of the patient	Male: Female	1.00 (0.90–1.12)
	Fever	Symptom (fever or not)	Yes: No	4.49 (3.84–5.27)
Chang LY et al., 2017 [25]	Age (y)	Age of the patient	0–2: 12–17	0.66 (0.60–0.71)
			3–5: 12–17	0.69 (0.64–0.74)
			6–11: 12–17	0.74 (0.69–0.79)
	Sex	Gender of the patient	Female: Male	0.96 (0.91–1.01)
	Season	Season of visit	Spring: Winter	1.06 (0.98–1.14)
			Summer: Winter	1.04 (0.96–1.12)
			Autumn: Winter	0.97 (0.90–1.04)
Ardoino I et al., 2019 [26]	Sex	Gender of the patient	Female: Male	0.92 (0.80–1.05)
	Age	Age of the patient	Older: Younger	1.01 (1.00–1.02)
	Previous hospitalisation	Hospitalization history	Yes: No	1.26 (1.09–1.46)
	Co-morbidities	Comorbidity status	Per one disease increase	1.05 (1.02–1.07)
Covino M et al., 2022 [27]	Age group	Age of the patient	2–5: <1	1.62 (1.53–1.73)
			6–10: <1	1.77 (1.64–1.91)
			11–18: <1	1.36 (1.25–1.49)
	Age group	Season of visit	Fall: Summer	0.93 (0.87–1.00)
			Winter: Summer	1.03 (0.96–1.09)
			Spring: Summer	0.98 (0.91–1.05)

Study	Original description	Summary of relevant factors	Description of associated factors	OR (95% CI)
	Pediatrician expertise	Practice duration of the physician	Longer: Shorter	1.22 (1.13–1.31)
	Fever on admission	Symptom (fever or not)	Yes: No	1.26 (1.11–1.42)
	Cough or not	Symptom (cough or not)	Yes: No	0.98 (0.94–1.03)
	Headache or not	Symptom (headache or not)	Yes: No	0.87 (0.76–0.99)
	Chest X-ray	Performing chest radiography or not	Negative: Not requested	1.82 (1.62–2.04)
			Positive: Not requested	4.47 (3.62–5.52)
Kourlaba G et al., 2016 [28]	Age (years)	Age of the patient	30–39: 20–29	1.18 (1.03–1.36)
			40–49: 20–29	1.09 (0.94–1.26)
			50–59: 20–29	1.17 (0.99–1.39)
			≥60: 20–29	1.12 (0.97–1.31)
	Sex	Gender of the patient	Male: Female	1.52 (1.38–1.67)
	Age	Age of the physician	41–50: <40	1.07 (0.86–1.33)
			51–60: <40	0.85 (0.70–1.05)
			≥60: <40	0.78 (0.64–0.97)
	Sex	Gender of the physician	Male: Female	0.99 (0.86–1.15)
Lin YC et al., 2010 [29]	Doctor age (years)	Age of the physician	41–50: <41	0.80 (0.74–0.86)
			>50: <41	0.90 (0.83–0.97)
	Doctor gender	Gender of the physician	Male: Female	1.03 (0.94–1.14)
	Patient age (years)	Age of the patient	45–64: 18–44	1.05 (0.98–1.11)
			>64: 18–44	1.11 (1.02–1.20)
Kronman MP et al., 2011 [30]	Patient age	Age of the patient	Older: Younger	1.2 (1.1–1.4)
	Obtaining a radiograph	Performing chest radiography or not	Yes: No	4.5 (1.6–12.6)
Lee ML et al., 2016 [31]	Sex	Gender of the patient	Female: Male	0.974 (0.954–0.994)
	Age (y)	Age of the patient	≤ 3–< 6: ≤ 0–< 3	0.996 (0.969–1.025)

Study	Original description	Summary of relevant factors	Description of associated factors	OR (95% CI)
			≤ 6–< 12: ≤ 0–< 3	0.865 (0.840–0.890)
			≤ 12–< 18: ≤ 0–< 3	0.841 (0.813–0.871)
	Season	Season of visit	Spring: Winter	1.027 (1.000–1.055)
			Summer: Winter	0.967 (0.939–0.997)
			Autumn: Winter	0.842 (0.818–0.867)
Fentie AM et al., 2022 [32]	Age in years	Age of the patient	18–39: 0–17	0.61 (0.38–0.86)
			40–64: 0–17	0.55 (0.39–0.93)
			≥65: 0–17	1.45 (0.31–3.59)
	Gender	Gender of the patient	Male: Female	1.18 (0.93–1.49)
	Hospitalisation history	Hospitalization history	No: Yes	0.85 (0.67–1.06)
	Length of hospitalisation in days	Hospitalization duration	Longer: Shorter	1.00 (0.99–1.01)
Forster CS et al., 2022 [33]	Gender	Gender of the patient	Male: Female	0.58 (0.28–1.21)
	Age	Age of the patient	≥36 mo: <36 mo	6.67 (3.53–12.92)
	Fever	Symptom (fever or not)	Yes: No	1.15 (0.64–2.12)
Goodman KE et al., 2023 [34]	Age group, y	Age of the patient	18–29: 50–59	0.67 (0.67–0.68)
			30–39: 50–59	0.83 (0.82–0.84)
			40–49: 50–59	1.01 (1.00–1.02)
			60–69: 50–59	0.96 (0.95–0.97)
			70–79: 50–59	0.85 (0.84–0.86)
			≥80: 50–59	0.73 (0.72–0.73)
	Publicly insured	Health insurance	Yes: No	1.00 (1.00–1.01)
	Sex	Gender of the patient	Male: Female	1.22 (1.22–1.23)
	Admission month	Season of visit	Fall/Winter: Spring/Summer	0.96 (0.96–0.97)
	Location	Settings	Urban: Rural	0.96 (0.88–1.05)

Study	Original description	Summary of relevant factors	Description of associated factors	OR (95% CI)
	Teaching status	Teaching status of facilities	Teaching: Non–teaching	0.88 (0.81–0.96)
	Bed size	Size of facilities	0–99: ≥500	0.91 (0.80–1.05)
			100–199: ≥500	0.96 (0.85–1.09)
			200–299: ≥500	1.03 (0.90–1.17)
			300–399: ≥500	0.98 (0.86–1.13)
			400–499: ≥500	0.85 (0.73–0.99)
Hadi U et al., 2008 [35]	Sex	Gender of the patient	Male: Female	1.08 (0.77–1.52)
	Age	Age of the patient	Adults: Children	0.63 (0.43–0.92)
	Residency	Settings	Urban: Rural	1.55 (1.09–2.20)
	Income	Income level of the patient	Lower: Higher	0.86 (0.62–1.20)
	Educational level	Educational level of the patient	Lower: Higher	1.10 (0.52–2.39)
	Insurance	Health insurance	No: Yes	1.31 (0.92–1.86)
Hersh AL et al., 2011 [36]	Age	Age of the patient	0–5: 6–12	1.27 (1.04–1.54)
			13–17: 6–12	1.16 (0.93–1.46)
	Insurance	Health insurance	Public: Private	0.79 (0.66–0.94)
Kawanami GH et al., 2011 [37]	Gender	Gender of the patient	Male: Female	0.91 (0.70–1.19)
	Age	Age of the patient	>60 y: ≤60 y	0.82 (0.62–1.07)
Jewell MJ et al., 2021 [38]	Age group (years)	Age of the patient	5–11: 12–17	0.93 (0.83–1.04)
			2–4: 12–17	0.80 (0.71–0.91)
	Gender	Gender of the patient	Female: Male	1.14 (1.05–1.24)
	Beds, n	Size of facilities	201–400: >400	1.49 (1.10–1.20)
			<200: >400	1.59 (1.13–2.24)
	Urban versus rural	Settings	Urban: Rural	0.74 (0.55–0.99)
	Teaching status	Teaching status of facilities	Teaching: Non–teaching	0.64 (0.48–0.84)

Study	Original description	Summary of relevant factors	Description of associated factors	OR (95% CI)
Hashimoto H et al., 2019 [39]	Patient age (AURI)	Age of the patient	0–9: ≥65	1.48 (1.46–1.50)
			10–19: ≥65	2.75 (2.69–2.82)
			20–64: ≥65	1.92 (1.89–1.94)
	Patient sex (AURI)	Gender of the patient	Male: Female	1.10 (1.08–1.11)
	Facility type (AURI)	Size of facilities	<200 beds: ≥500 beds	2.07 (1.97–2.18)
			200–499 beds : ≥500 beds	1.71 (1.62–1.80)
	Patient age (gastrointestinal tract infections, GTI)	Age of the patient	0–9: ≥65	1.76 (1.71–1.82)
			10–19: ≥65	1.92 (1.83–2.00)
			20–64: ≥65	1.55 (1.51–1.60)
	Patient sex (GTI)	Gender of the patient	Male: Female	1.04 (1.01–1.06)
	Facility type (GTI)	Size of facilities	<200 beds: ≥500 beds	1.17 (1.04–1.32)
			200–499 beds : ≥500 beds	0.93 (0.82–1.05)
Hadi U et al., 2008 [40]	Sex	Gender of the patient	Male: Female	1.23 (1.01–1.51)
	Age	Age of the patient	≥18: <18	0.34 (0.27–0.43)
	Geographic provenance	Settings	Urban: Rural	0.97 (0.79–1.19)
	Health insurance	Health insurance	No: Yes	0.78 (0.63–0.97)
	Education	Educational level of the patient	Lower: Higher	1.03 (0.70–1.53)
	Income	Income level of the patient	Lower: Higher	0.87 (0.71–1.06)
Stone S et al., 2000 [41]	Age	Age of the patient	<18: ≥18	0.32 (0.20–0.52)
	Sex	Gender of the patient	Female: Male	0.93 (0.60–1.40)
Vanderweil SG et al., 2008 [42]	Age group (NHAMCS)	Age of the patient	10–19: 2–9	1.1 (0.8–1.6)
			20–29: 2–9	1.3 (0.9–1.9)
			30–39: 2–9	1.4 (1.0–1.9)
			40–49: 2–9	1.3 (0.9–1.9)

Study	Original description	Summary of relevant factors	Description of associated factors	OR (95% CI)
			50–54: 2–9	1.6 (0.9–2.7)
	Gender (NHAMCS)	Gender of the patient	Female: Male	1.1 (0.9–1.4)
	Urban status (NHAMCS)	Settings	Urban: Rural	0.5 (0.4–0.7)
	Type of insurance (NHAMCS)	Health insurance	Public: Private	1.3 (1.0–1.6)
	Age group (NEDSS)	Age of the patient	20–29: 14–19	1.6 (1.1–2.3)
			30–39: 14–19	1.7 (1.1–2.7)
			40–49: 14–19	2.0 (1.4–2.8)
			50–54: 14–19	3.3 (2.1–5.0)
	Gender (NEDSS)	Gender of the patient	Female: Male	1.1 (0.8–1.3)
	Symptom onset to ED (NEDSS)	Time of symptoms last	1–3 days: <1 day	2.1 (1.6–2.8)
			4–7 days: <1 day	2.7 (1.9–3.8)
			>7 days: <1 day	3.2 (2.1–4.7)
Thu TA et al., 2012 [43]	Age group, years (total use)	Age of the patient	30–59: <30	0.8 (0.7–1.0)
			≥60: <30	0.7 (0.6–1.0)
	Sex (total use)	Gender of the patient	Male: Female	1.2 (1.1–1.3)
	Age, years (inappropriate use)	Age of the patient	30–59: <30	1.0 (0.8–1.1)
			≥60: <30	0.8 (0.7–1.0)
	Sex (inappropriate use)	Gender of the patient	Male: Female	1.6 (0.9–1.8)
Shapiro DJ et al., 2014 [44]	Age (years)	Age of the patient	40–59: 18–39	1.46 (1.29–1.66)
			≥60: 18–39	1.54 (1.31–1.80)
			Others: White	1.24 (0.92–1.66)
	Insurance	Health insurance	Public: Private	0.78 (0.67–0.89)
	Comorbidity	Comorbidity status	Yes: No	1.16 (1.01–1.34)
Sikkens JJ et al., 2018 [45]	Gender	Gender of the patient	Male: Female	0.86 (0.51–1.45)

Study	Original description	Summary of relevant factors	Description of associated factors	OR (95% CI)
	Clinical experience	Practice duration of the physician	>1: <1	2.09 (1.26–3.38)
Seni J et al., 2020 [46]	Age category (years)	Age of the patient	<2: ≥2	2.64 (1.78–3.94)
	Gender	Gender of the patient	Female: Male	0.73 (0.56–0.96)
	Previous hospitalisation	Hospitalization history	Yes: No	0.58 (0.37–0.91)
Sencan I et al., 2022 [47]	Gender	Gender of the patient	Male: Female	1.1 (0.8–1.3)
Xavier SP et al., 2022 [48]	Age	Age of the patient	28 days–12 months: 1–2 years	0.75 (0.382–1.482)
			3–5 years: 1–2 years	1.16 (0.526–2.574)
			6–10 years: 1–2 years	1.20 (0.432–3.332)
	Number of antibiotics per prescription	Number of antibiotics per prescription	2: 1	1.40 (0.819–2.406)
			≥3: 1	2.83 (1.245–6.462)
	Hospitalization time (days)	Hospitalization duration	Shorter (< 4): Longer (≥ 5)	1.88 (1.133–3.140)
Shin SM et al., 2015 [49]	Gender	Gender of the patient	Female: Male	0.93 (0.92–0.94)
	Age (yr)	Age of the patient	7–12: 2–6	0.85 (0.85–0.86)
			13–17: 2–6	0.83 (0.82–0.84)
Zhang Z et al., 2017 [50]	Gender	Gender of the patient	Male: Female	0.9 (0.8–1.0)
	Age	Age of the patient	6–14 years: 2–5 years	1.3 (1.2–1.5)
	Payment method	Health insurance	With insurance copayment: Without	5.0 (4.1–6.0)
Teixeira Rodrigues A et al., 2016 [51]	Age (years)	Age of the patient	Median	0.99 (0.95–1.03)
	Gender	Gender of the patient	Female: Male	0.72 (0.39–1.33)
	Patients per day	Daily patient volume	25th and 75th percentile /n	0.97 (0.94–1.00)
Velasco E et al., 2011 [52]	Sex	Gender of the patient	Male: Female	1.81 (1.42–2.31)
	Age group	Age of the patient	50–59: 20–29	1.56 (1.10–2.21)
Havers FP et al., 2018 [53]	Age group, y	Age of the patient	<5: 18–50	0.45 (0.36–0.55)
			5–18: 18–50	0.70 (0.58–0.83)

Study	Original description	Summary of relevant factors	Description of associated factors	OR (95% CI)
			50–65: 18–50	1.39 (1.18–1.64)
			65–80: 18–50	1.51 (1.24–1.83)
			≥80: 18–50	2.21 (1.53–2.94)
	Sex	Gender of the patient	Male: Female	1.02 (0.90–1.15)
	Time after symptom onset, d	Time of symptoms last	3–4: ≤2	1.22 (1.06–1.41)
			5–7: ≤2	1.31 (1.12–1.52)
Nunez-Nunez M et al., 2022 [54]	Gender	Gender of the patient	Female: Male	1.26 (1.03–1.53)
	Age	Age of the patient	Older: Younger	1.05 (1.01–1.02)
Salzo A et al., 2021 [55]	Gender	Gender of the patient	Female: Male	0.5 (0.3–0.9)
	Days of hospitalization	Hospitalization duration	4–7 days: 1–3 days	2.6 (1.1–5.8)
			8–14 days: 1–3 days	0.4 (0.1–0.9)
			≥2 weeks: 1–3 days	0.5 (0.2–1.4)
Opoku MM et al., 2020 [56]	Age of patient in years	Age of the patient	≥5: <5	0.40 (0.32–0.51)
	Sex of patient	Gender of the patient	Female: Male	1.05 (0.87–1.27)
	Prescriber’s years of practice	Practice duration of the physician	3–5: <3	1.12 (0.84–1.48)
			6–9: <3	2.97 (1.99–4.44)
			≥10: <3	1.60 (1.12–2.27)
	Cough or not	Symptom (cough or not)	Yes: No	3.54 (2.54–4.92)
Nepal A et al., 2020 [57]	Gender	Gender of the patient	Female: Male	0.863 (0.779–0.956)
	Age group	Age of the patient	5 to 14 years: Less than 5 years	0.515 (0.398–0.666)
			15 to 24 years: Less than 5 years	0.288 (0.225–0.370)
			25 to 44 years: Less than 5 years	0.273 (0.214–0.347)
			45 to 64 years: Less than 5 years	0.313 (0.244–0.402)
			65 and above: Less than 5 years	0.430 (0.330–0.560)

Study	Original description	Summary of relevant factors	Description of associated factors	OR (95% CI)
Morley VJ et al., 2020 [58]	Sex	Gender of the patient	Male: Female	1.68 (1.17–2.41)
McKay R et al., 2019 [59]	Season	Season of visit	Mar–May: Jun–Aug	0.99 (0.97–1.02)
			Sep–Nov: Jun–Aug	0.80 (0.77–0.82)
			Dec–Feb: Jun–Aug	0.95 (0.92–0.98)
	Patient sex	Gender of the patient	Female: Male	0.97 (0.96–0.98)
	Patient age	Age of the patient	Per y increase	1.08 (1.079–1.081)
	Physician sex	Gender of the physician	Female: Male	0.91 (0.86–0.96)
	Daily patient volume	Daily patient volume	2nd quartile: 1st quartile	0.99 (0.98–1.01)
			3rd quartile: 1st quartile	0.97 (0.95–0.99)
			4th quartile: 1st quartile	0.94 (0.92–0.97)
	Years since medical school graduation	Practice duration of the physician	6–10: 0–5	0.99 (0.94–1.04)
			11–15: 0–5	1.08 (1.02–1.15)
			16–20: 0–5	1.16 (1.09–1.24)
			21–25: 0–5	1.23 (1.15–1.31)
			26–30: 0–5	1.32 (1.23–1.41)
			31–35: 0–5	1.35 (1.26–1.46)
			36–40: 0–5	1.39 (1.29–1.51)
			>40: 0–5	1.46 (1.33–1.61)
Okoro RN et al., 2019 [60]	Age group (years)	Age of the patient	5–11: <5	0.70 (0.32–1.5)
			12–59: <5	0.44 (0.24–0.83)
			≥60: <5	0.21 (0.09–0.50)
	Gender	Gender of the patient	Male: Female	1.26 (0.92–1.74)
Monteiro LGS et al., 2017 [61]	Gender	Gender of the patient	Male: Female	1.244 (0.359–4.313)
	Age	Age of the patient	≥5: <5	5.496 (1.541–7.595)

Study	Original description	Summary of relevant factors	Description of associated factors	OR (95% CI)
Manne M et al., 2018 [62]	Age (y)	Age of the patient	≥65: <65	0.831 (0.779–0.887)
	Temperature, ° F	Symptom (fever or not)	≥ 100.4: < 100.4	1.663 (1.463–1.889)
	Asthma	Asthma history	Yes: No	1.121 (1.063–1.181)
Paul IM et al., 2011 [63]	Gender（1998-2007）	Gender of the patient	Female: Male	0.89 (0.60–1.34)
	Age, y（1998-2007）	Age of the patient	5–11: <5	0.87 (0.53–1.43)
			12–17: <5	0.65 (0.39–1.08)
	Season, month（1998-2007）	Season of visit	September to November: June to August	1.56 (0.86–2.85)
			December to February: June to August	1.92 (1.05–3.52)
			March to May: June to August	1.04 (0.54–2.00)
	Gender（2001-2007）	Gender of the patient	Female: Male	0.99 (0.56–1.77)
	Age, y（2001-2007）	Age of the patient	5–11: <5	0.87 (0.43–1.76)
			12–17: <5	0.76 (0.38–1.51)
	Season, month（2001-2007）	Season of visit	September to November: June to August	1.66 (0.64–4.30)
			December to February: June to August	1.73 (0.70–4.32)
			March to May: June to August	1.47 (0.56–3.88)
Moro ML et al., 2009 [64]	Child's age	Age of the patient	>2 years: ≤2 years	0.98 (0.80–1.19)
	Fever	Symptom (fever or not)	>38°C: ≤38°C	2.34 (1.97–2.79)
	Physician age	Age of the physician	For 5 years of age increase	1.02 (0.95–1.10)
Osatakul S et al., 2007 [65]	Duration of diarrhoea (days)	Illness duration	4–7: 1–3	0.719 (0.547–0.946)
	Body temperature (° C)	Symptom (fever or not)	37.6–38.5: 37.5	1.213 (0.957–1.538)
			38.6–39.5: 37.5	1.869 (1.170–2.986)
			>39.5: 37.5	1.533 (0.972–2.417)
Rutschmann OT et al., 2004 [66]	Age	Age of the patient	45–64: 18–44	0.96 (0.68–1.36)
			≥65: 18–44	0.80 (0.46–1.40)

Study	Original description	Summary of relevant factors	Description of associated factors	OR (95% CI)
	Female vs male	Gender of the patient	Female: Male	1.03 (0.73–1.44)
	Rural area vs urban	Settings	Rural: Urban	1.28 (0.80–2.05)
	X-ray during visit vs not X-ray	Performing chest radiography or not	Yes: No	1.06 (0.64–1.74)
Nsofor CA et al., 2016 [67]	Gender	Gender of the patient	Male: 424/750<br>Female: 462/835	
	Age	Age of the patient	0–12 years: 301/511<br>13–20 years: 125/224<br>21–50 years: 157/274<br>56–80 years: 303/576	
Nadeem Ahmed M et al., 2010 [68]	Age at visits	Age of the patient	3–5 years: 0–2 years	1.40 (0.68–2.88)
			6–10 years: 0–2 years	2.04 (1.01–4.14)
			16–10 years: 0–2 years	1.61 (0.78–3.30)
	Gender	Gender of the patient	Female: Male	0.75 (0.54–1.02)
	Health insurance	Health insurance	Government: Private	1.14 (0.76–1.72)
	Comorbidity	Comorbidity status	Yes: No	1.15 (0.71–1.85)
	Fever	Symptom (fever or not)	Yes: No	1.53 (1.10–2.13)
	Cough	Symptom (cough or not)	Yes: No	0.91 (0.63–1.31)
Aspinall SL et al., 2009 [69]	Comorbid condition	Comorbidity status	Yes: No	2.1 (1.2–3.5)
	Fever, self-report	Symptom (fever or not)	Yes: No	2.5 (1.4–4.4)
	Provider age	Age of the physician	≥30 y: <30 y	2.6 (1.1–6.3)
Tham DWJ et al., 2020 [70]	Gender	Gender of the patient	Male*Antibiotic: 134<br>Male*No antibiotic: 164<br>Female*Antibiotic: 105<br>Female*No antibiotic: 146	

Study	Original description	Summary of relevant factors	Description of associated factors	OR (95% CI)
	Fever	Symptom (fever or not)	Fever*Antibiotic: 203<br>Fever*No antibiotic: 207<br>Not fever*Antibiotic: 36<br>Not fever*No antibiotic: 103	
Ahmad A et al., 2021 [71]	Age	Age of the patient	Older: Younger	1.01 (0.99–1.03)
	Gender	Gender of the patient	Female: Male	1.05 (0.52–2.01)
	Fever	Symptom (fever or not)	Yes: No	1.71 (0.70–4.15)
	Cough	Symptom (cough or not)	Yes: No	1.87 (0.61–5.71)
	Duration of illness	Illness duration	≤ 1 day: > 1 day	8.63 (1.07–69.54)
Sawaya RD et al., 2020 [72]	Age	Age of the patient	Per month increase	1.04 (1.02–1.06)
	Gender	Gender of the patient	Female: Male	0.68 (0.47–0.98)
	Height of fever in the ED	Symptom (fever or not)	38–39.4°C: <38°C	0.47 (0.32–0.67)
Steinberg MB et al., 2016 [73]	Gender	Gender of the patient	Female: Male	0.92 (0.81–1.05)
	Age (years)	Age of the patient	25–44: 18–24	1.16 (0.86–1.56)
			45–64: 18–24	0.97 (0.73–1.29)
			≥65: 18–24	0.67 (0.48–0.92)
Zhao H et al., 2020 [74]	Age group (years)	Age of the patient	<6: 18–44	0.97 (0.97–0.98)
			6–17: 18–44	1.21 (1.21–1.22)
			45–64: 18–44	0.83 (0.82–0.83)
			≥65: 18–44	0.67 (0.67–0.67)
	Gender	Gender of the patient	Female: Male	0.92 (0.92–0.93)
	Payment type	Health insurance	Full out–of–pocket: Insurance	0.91 (0.91–0.91)
	Season of visit	Season of visit	Spring: Summer	1.00 (1.00–1.00)
			Autumn: Summer	0.97 (0.96–0.97)

Study	Original description	Summary of relevant factors	Description of associated factors	OR (95% CI)
			Winter: Summer	1.07 (1.07–1.07)

OR - odds ratio, CI - confidence interval.

References for included studies

1. Ciofi DAM, D'Amore C, Ceradini J, Paolini V, Ciliento G, Chessa G, et al. Prevalence of antibiotic use in a tertiary care hospital in Italy, 2008-2016. Ital J Pediatr. 2019;45(1):63.
2. Denny KJ, Gartside JG, Alcorn K, Cross JW, Maloney S, Keijzers G. Appropriateness of antibiotic prescribing in the emergency department. J Antimicrob Chemoth. 2019;74(2):515-20.
3. Desai NM, Sadlowski JL, Mistry RD. Antibiotic prescribing for viral respiratory infections in the pediatric emergency department and urgent care. Pediatr Infect Dis J. 2020;39(5):406-10.
4. Anteneh DA, Kifle ZD, Mersha GB, Ayele TT. Appropriateness of antibiotics use and associated factors in hospitalized patients at University of Gondar Specialized Hospital, Amhara, Ethiopia: prospective follow-up study. Inquiry. 2021;58(2):1448312824.
5. Boone K, Morris SK, Doshi S, Black J, Mohsin M, Ahmed T, et al. Antimicrobial prescribing during infant hospital admissions in a birth cohort in Dhaka, Bangladesh. J Trop Pediatrics. 2021;67(3):a93.
6. Akkawi ME, Taffour RM, AL-Shami AM. Evaluation of antibiotic prescribing pattern and appropriateness among hospitalized pediatric patients: findings from a Malaysian teaching hospital. Infect Dis Rep. 2022;14(6):889-99.
7. Cotter JM, Florin TA, Moss A, Suresh K, Ramgopal S, Navanandan N, et al. Factors associated with antibiotic use for children hospitalized with pneumonia. Pediatrics. 2022;150(2):e2021054677.
8. Dilworth TJ, Hietpas K, Kram J, Baumgardner D. Impact of geodemographic factors on antibiotic prescribing for acute, uncomplicated bronchitis or upper respiratory tract infection. J Am Board Fam Med. 2022;35(4):733-41.
9. Kitt E, Hayes M, Ballester L, Sewawa KB, Mulale U, Mazhani L, et al. Assessing antibiotic utilization among pediatric patients in Gaborone, Botswana. Sage Open Med. 2022;10:1-10.
10. Kroening-Roche JC, Soroudi A, Castillo EM, Vilke GM. Antibiotic and bronchodilator prescribing for acute bronchitis in the emergency department. J Emerg Med. 2012;43(2):221-7.

11. Linder JA, Singer DE. Desire for antibiotics and antibiotic prescribing for adults with upper respiratory tract infections. J Gen Intern Med. 2003;18(10):795-801.

12. Kornblith AE, Fahimi J, Kanzaria HK, Wang RC. Predictors for under-prescribing antibiotics in children with respiratory infections requiring antibiotics. Am J Emerg Med. 2018;36(2):218-25.

13. Kozyrskyj AL, Dahl ME, Chateau DG, Mazowita GB, Klassen TP, Law BJ. Evidence-based prescribing of antibiotics for children: role of socioeconomic status and physician characteristics. Can Med Assoc J. 2004;171(2):139-45.

14. Curt AM, Lipsett SC, Neuman MI. Antibiotic prescribing and parent satisfaction for children with respiratory illness. Clin Pediatr. 2020;59(6):618-21.

15. Kumar R, Indira K, Rizvi A, Rizvi T, Jeyaseelan L. Antibiotic prescribing practices in primary and secondary health care facilities in Uttar Pradesh, India. J Clin Pharm Ther. 2008;33(6):625-34.

16. Cantrell R, Young AF, Martin BC. Antibiotic prescribing in ambulatory care settings for adults with colds, upper respiratory tract infections, and bronchitis. Clin Ther. 2002;24(1):170-82.

17. Arnold SR, To T, McIsaac WJ, Wang EE. Antibiotic prescribing for upper respiratory tract infection: the importance of diagnostic uncertainty. J Pediatr-Us. 2005;146(2):222-6.

18. Cadieux G, Abrahamowicz M, Dauphinee D, Tamblyn R. Are physicians with better clinical skills on licensing examinations less likely to prescribe antibiotics for viral respiratory infections in ambulatory care settings? Med Care. 2011;49(2):156-65.

19. Barnett ML, Linder JA. Antibiotic prescribing for adults with acute bronchitis in the United States, 1996-2010. Jama-J Am Med Assoc. 2014;311(19):2020-2.

20. Bergmark RW, Sedaghat AR. Antibiotic prescription for acute rhinosinusitis: emergency departments versus primary care providers. Laryngoscope. 2016;126(11):2439-44.

21. Depew RE, Gonzales G. Differences in US antibiotic prescription use by facility and patient characteristics: evidence from the National Ambulatory Medical Care Survey. Fam Pract. 2020;37(2):180-6.

22. Coco AS, Horst MA, Gambler AS. Trends in broad-spectrum antibiotic prescribing for children with acute otitis media in the United States, 1998-2004. Bmc Pediatr. 2009;9:41.

23. Copp HL, Shapiro DJ, Hersh AL. National ambulatory antibiotic prescribing patterns for pediatric urinary tract infection, 1998-2007. Pediatrics. 2011;127(6):1027-33.

24. Ababneh MA, Al-Azzam SI, Ababneh R, Rababa'H AM, Demour SA. Antibiotic prescribing for acute respiratory infections in children in Jordan. Int Health. 2017;9(2):124-30.

25. Chang LY, Lai CC, Chen CJ, Cho CY, Luo YC, Jeng MJ, et al. Recent trends in prescribing antibiotics for acute tonsillitis in pediatric ambulatory care in Taiwan, 2000-2009: a nationwide population-based study. J Microbiol Immunol. 2017;50(4):500-6.

26. Ardoino I, Mannucci PM, Nobili A, Franchi C. Antibiotic use and associated factors in a large sample of hospitalised older people. J Glob Antimicrob Re. 2019;19:167-72.
27. Covino M, Buonsenso D, Gatto A, Morello R, Curatole A, Simeoni B, et al. Determinants of antibiotic prescriptions in a large cohort of children discharged from a pediatric emergency department. Eur J Pediatr. 2022;181(5):2017-30.
28. Kourlaba G, Gkrania-Klotsas E, Kourkouni E, Mavrogeorgos G, Zaoutis TE. Antibiotic prescribing and expenditures in outpatient adults in Greece, 2010 to 2013: evidence from real-world practice. Eurosurveillance. 2016;21(26):30266.
29. Lin YC, Lin HC, Lin HC. Doctor characteristics and prescribing antibiotics for urinary tract infections: the experience of an Asian country. J Eval Clin Pract. 2010;16(6):1221-6.
30. Kronman MP, Hersh AL, Feng R, Huang YS, Lee GE, Shah SS. Ambulatory visit rates and antibiotic prescribing for children with pneumonia, 1994-2007. Pediatrics. 2011;127(3):411-8.
31. Lee ML, Cho CY, Hsu CL, Chen CJ, Chang LY, Lee YS, et al. Recent trends in antibiotic prescriptions for acute respiratory tract infections in pediatric ambulatory care in Taiwan, 2000-2009: a nationwide population-based study. J Microbiol Immunol. 2016;49(4):554-60.
32. Fentie AM, Degefaw Y, Asfaw G, Shewarega W, Woldearegay M, Abebe E, et al. Multicentre point-prevalence survey of antibiotic use and healthcare-associated infections in Ethiopian hospitals. Bmj Open. 2022;12(2):e54541.
33. Forster CS, Almaazi A, Hamdy R, Harik N. Predictors of empiric antibiotic use in the emergency department in children without urinary tract infections. Pediatr Emerg Care. 2022;38(5):e1251-6.
34. Goodman KE, Baghdadi JD, Magder LS, Heil EL, Sutherland M, Dillon R, et al. Patterns, predictors, and intercenter variability in empiric Gram-Negative antibiotic use across 928 United States hospitals. Clin Infect Dis. 2023;76(3):e1224-35.
35. Hadi U, Duerink DO, Lestari ES, Nagelkerke NJ, Keuter M, Huis IVD, et al. Audit of antibiotic prescribing in two governmental teaching hospitals in Indonesia. Clin Microbiol Infec. 2008;14(7):698-707.
36. Hersh AL, Shapiro DJ, Pavia AT, Shah SS. Antibiotic prescribing in ambulatory pediatrics in the United States. Pediatrics. 2011;128(6):1053-61.
37. Kawanami GH, Fortaleza CM. Factors predictive of inappropriateness in requests for parenteral antimicrobials for therapeutic purposes: a study in a small teaching hospital in Brazil. Scand J Infect Dis. 2011;43(6-7):528-35.
38. Jewell MJ, Leyenaar J, Shieh MS, Pekow PS, Stefan M, Lindenauer PK. Unnecessary antibiotic prescribing in children hospitalised for asthma exacerbation: a retrospective national cohort study. Bmj Qual Saf. 2021;30(4):292-9.
39. Hashimoto H, Matsui H, Sasabuchi Y, Yasunaga H, Kotani K, Nagai R, et al. Antibiotic prescription among outpatients in a prefecture of Japan, 2012-2013: a

retrospective claims database study. Bmj Open. 2019;9(4):e26251.
40. Hadi U, Duerink DO, Lestari ES, Nagelkerke NJ, Werter S, Keuter M, et al. Survey of antibiotic use of individuals visiting public healthcare facilities in Indonesia. Int J Infect Dis. 2008;12(6):622-9.
41. Stone S, Gonzales R, Maselli J, Lowenstein SR. Antibiotic prescribing for patients with colds, upper respiratory tract infections, and bronchitis: a national study of hospital-based emergency departments. Ann Emerg Med. 2000;36(4):320-7.
42. Vanderweil SG, Tsai CL, Pelletier AJ, Espinola JA, Sullivan AF, Blumenthal D, et al. Inappropriate use of antibiotics for acute asthma in United States emergency departments. Acad Emerg Med. 2008;15(8):736-43.
43. Thu TA, Rahman M, Coffin S, Harun-Or-Rashid M, Sakamoto J, Hung NV. Antibiotic use in Vietnamese hospitals: a multicenter point-prevalence study. Am J Infect Control. 2012;40(9):840-4.
44. Shapiro DJ, Hicks LA, Pavia AT, Hersh AL. Antibiotic prescribing for adults in ambulatory care in the USA, 2007-09. J Antimicrob Chemoth. 2014;69(1):234-40.
45. Sikkens JJ, Gerritse SL, Peters E, Kramer M, van Agtmael MA. The 'morning dip' in antimicrobial appropriateness: circumstances determining appropriateness of antimicrobial prescribing. J Antimicrob Chemoth. 2018;73(6):1714-20.
46. Seni J, Mapunjo SG, Wittenauer R, Valimba R, Stergachis A, Werth BJ, et al. Antimicrobial use across six referral hospitals in Tanzania: a point prevalence survey. Bmj Open. 2020;10(12):e42819.
47. Sencan I, Cag Y, Karabay O, Kurtaran B, Guclu E, Ogutlu A, et al. Antibiotic use and influencing factors among hospitalized patients with COVID-19: a multicenter point-prevalence study from Turkey. Balk Med J. 2022;39(3):209-17.
48. Xavier SP, Victor A, Cumaquela G, Vasco MD, Rodrigues O. Inappropriate use of antibiotics and its predictors in pediatric patients admitted at the Central Hospital of Nampula, Mozambique. Antimicrob Resist in. 2022;11(1):79.
49. Shin SM, Shin JY, Kim MH, Lee SH, Choi S, Park BJ. Prevalence of antibiotic use for pediatric acute upper respiratory tract infections in Korea. J Korean Med Sci. 2015;30(5):617-24.
50. Zhang Z, Hu Y, Zou G, Lin M, Zeng J, Deng S, et al. Antibiotic prescribing for upper respiratory infections among children in rural China: a cross-sectional study of outpatient prescriptions. Global Health Action. 2017;10(1):1287334.
51. Teixeira RA, Ferreira M, Pineiro-Lamas M, Falcao A, Figueiras A, Herdeiro MT. Determinants of physician antibiotic prescribing behavior: a 3 year cohort study in Portugal. Curr Med Res Opin. 2016;32(5):949-57.
52. Velasco E, Espelage W, Faber M, Noll I, Ziegelmann A, Krause G, et al. A national cross-sectional study on socio-behavioural factors that influence physicians' decisions to begin antimicrobial therapy. Infection. 2011;39(4):289-97.

53. Havers FP, Hicks LA, Chung JR, Gaglani M, Murthy K, Zimmerman RK, et al. Outpatient antibiotic prescribing for acute respiratory infections during influenza seasons. Jama Netw Open. 2018;1(2):e180243.
54. Nunez-Nunez M, Perez-Galera S, Antonio Giron-Ortega J, Sandoval Fernandez-Del-Castillo S, Beltran-Garcia M, De Cueto M, et al. Predictors of inappropriate antimicrobial prescription: eight-year point prevalence surveys experience in a third level hospital in Spain. Front Pharmacol. 2022;13:1018158.
55. Salzo A, Ripabelli G, Sammarco ML, Mariano A, Niro C, Tamburro M. Healthcare-associated infections and antibiotics consumption: a comparison of point prevalence studies and intervention strategies. Hosp Top. 2021;99(3):140-50.
56. Opoku MM, Bonful HA, Koram KA. Antibiotic prescription for febrile outpatients: a health facility-based secondary data analysis for the Greater Accra region of Ghana. Bmc Health Serv Res. 2020;20(1):978.
57. Nepal A, Hendrie D, Robinson S, Selvey LA. Analysis of patterns of antibiotic prescribing in public health facilities in Nepal. J Infect Dev Countr. 2020;14(1):18-27.
58. Morley VJ, Firgens E, Vanderbilt RR, Zhou Y, Zook M, Read AF, et al. Factors associated with antibiotic prescribing for acute bronchitis at a university health center. Bmc Infect Dis. 2020;20(1):177.
59. McKay R, Patrick DM, McGrail K, Law MR. Antibiotic prescribing for pediatric respiratory infections: what explains a large variation among physicians? Can Fam Physician. 2019;65(6):e278-91.
60. Okoro RN, Nmeka C, Erah PO. Antibiotics prescription pattern and determinants of utilization in the national health insurance scheme at a tertiary hospital in Nigeria. Afr Health Sci. 2019;19(3):2356-64.
61. Monteiro L, Chauque A, Barros MP, Ira TR. Determinants of antibiotic prescription in paediatric patients: the case of two hospitals in Maputo, Mozambique. S Afr J Child Health. 2017;11(3):109-11.
62. Manne M, Deshpande A, Hu B, Patel A, Taksler GB, Misra-Hebert AD, et al. Provider variation in antibiotic prescribing and outcomes of respiratory tract infections. South Med J. 2018;111(4):235-42.
63. Paul IM, Maselli JH, Hersh AL, Boushey HA, Nielson DW, Cabana MD. Antibiotic prescribing during pediatric ambulatory care visits for asthma. Pediatrics. 2011;127(6):1014-21.
64. Moro ML, Marchi M, Gagliotti C, Di Mario S, Resi D. Why do paediatricians prescribe antibiotics? Results of an Italian regional project. Bmc Pediatr. 2009;9:69.
65. Osatakul S, Puetpaiboon A. Appropriate use of empirical antibiotics in acute diarrhoea: a cross-sectional survey in southern Thailand. Ann Trop Paediatr. 2007;27(2):115-22.
66. Rutschmann OT, Domino ME. Antibiotics for upper respiratory tract infections in ambulatory practice in the United States, 1997-1999: does physician specialty matter? J Am Board Fam Pract. 2004;17(3):196-200.

67. Nsofor CA, Amadi ES, Obijuru CE, Ohalete CV, Ukwandu N. Prevalence of antimicrobial use in major hospitals in Owerri, Nigeria. EC Microbiology. 2016;3(5):522-7.
68. Nadeem AM, Muyot MM, Begum S, Smith P, Little C, Windemuller FJ. Antibiotic prescription pattern for viral respiratory illness in emergency room and ambulatory care settings. Clin Pediatr. 2010;49(6):542-7.
69. Aspinall SL, Good CB, Metlay JP, Mor MK, Fine MJ. Antibiotic prescribing for presumed nonbacterial acute respiratory tract infections. Am J Emerg Med. 2009;27(5):544-51.
70. Tham D, Abubakar U, Tangiisuran B. Prevalence and predictors of antibiotic use among children visiting the emergency department in a tertiary hospital in Malaysia. Eur J Pediatr. 2020;179(5):743-8.
71. Ahmad A, Nor J, Abdullah AA, Tuan KT, Yazid MB. Patient factors in inappropriate antibiotic prescribing for upper respiratory tract infection in the emergency department. Malays J Med Sci. 2021;28(2):72-83.
72. Sawaya RD, El ZT, Mrad S, Abdul MC, Shaya S, Makki M, et al. Comparing febrile children presenting on and off antibiotics to the emergency department: a retrospective cohort study. Bmc Pediatr. 2020;20(1):117.
73. Steinberg MB, Akincigil A, Kim EJ, Shallis R, Delnevo CD. Tobacco smoking as a risk factor for increased antibiotic prescription. Am J Prev Med. 2016;50(6):692-8.
74. Zhao H, Bian J, Han X, Zhang M, Zhan S. Outpatient antibiotic use associated with acute upper respiratory infections in China: a nationwide cross-sectional study. Int J Antimicrob Ag. 2020;56(6):106193.

Supplementary Figure 1. Forest plot of outpatient antibiotic prescribing

Study	Events	Total	Prevalence	95%CI	Weight
Yi S et al., 2022	713	1082	0.659	[0.630; 0.687]	1.1%
Zhao H et al., 2021	1529384	11911432	0.128	[0.128; 0.129]	1.1%
Zhao H et al., 2021	5639535	49739475	0.113	[0.113; 0.113]	1.1%
Zhao H et al., 2021	5266957	49233504	0.107	[0.107; 0.107]	1.1%
Zhao H et al., 2021	4935667	47946364	0.103	[0.103; 0.103]	1.1%
Zhao H et al., 2021	1477321	13873342	0.106	[0.106; 0.107]	1.1%
Almansoori N et al., 2021	195	1454	0.134	[0.117; 0.153]	1.1%
Atal S et al., 2021	117	503	0.233	[0.196; 0.272]	1.1%
Atal S et al., 2021	140	1008	0.139	[0.118; 0.162]	1.1%
Atal S et al., 2021	282	1208	0.233	[0.210; 0.258]	1.1%
Shrestha JTM et al., 2021	138	559	0.247	[0.212; 0.285]	1.1%
Wendie TF et al., 2021	660	1500	0.440	[0.415; 0.466]	1.1%
Ofori−Adjei YA et al., 2019	44	678	0.065	[0.048; 0.086]	1.1%
Li H et al., 2019	9156	45397	0.202	[0.198; 0.205]	1.1%
Li H et al., 2019	10174	58135	0.175	[0.172; 0.178]	1.1%
Li H et al., 2019	9036	59484	0.152	[0.149; 0.155]	1.1%
Li H et al., 2019	8414	59463	0.141	[0.139; 0.144]	1.1%
Li H et al., 2019	7762	60000	0.129	[0.127; 0.132]	1.1%
Yimenu DK et al., 2019	418	600	0.697	[0.658; 0.733]	1.1%
Komagamine J et al., 2020	153	1972	0.078	[0.066; 0.090]	1.1%
Tham DWJ et al., 2020	239	549	0.435	[0.393; 0.478]	1.1%
Khan Z et al., 2020	194	300	0.647	[0.590; 0.701]	1.1%
Khan Z et al., 2020	156	300	0.520	[0.462; 0.578]	1.1%
Yilma Z et al., 2020	410	1000	0.410	[0.379; 0.441]	1.1%
Yilma Z et al., 2020	225	384	0.586	[0.535; 0.636]	1.1%
Nepal A et al., 2020	1470	3980	0.369	[0.354; 0.385]	1.1%
Frost HM et al., 2020	2224	23722	0.094	[0.090; 0.098]	1.1%
Bimba HV et al., 2020	772	2128	0.363	[0.342; 0.384]	1.1%
Wang CN et al., 2020	63729	199147	0.320	[0.318; 0.322]	1.1%
Denny KJ et al., 2019	1019	7497	0.136	[0.128; 0.144]	1.1%
Croche Santander B et al., 2018	104	630	0.165	[0.137; 0.196]	1.1%
Mengistu G et al., 2020	511	1200	0.426	[0.398; 0.454]	1.1%
Hussain S et al., 2018	197	1000	0.197	[0.173; 0.223]	1.1%
Wang S et al., 2018	2089	4800	0.435	[0.421; 0.449]	1.1%
Zeng L et al., 2017	311	500	0.622	[0.578; 0.665]	1.1%
Sisay M et al., 2017	868	1500	0.579	[0.553; 0.604]	1.1%
Fleming−Dutra KE et al., 2016	15393	141202	0.109	[0.107; 0.111]	1.1%
Fleming−Dutra KE et al., 2016	8352	42830	0.195	[0.191; 0.199]	1.1%
Lee ML et al., 2016	346157	1494613	0.232	[0.231; 0.232]	1.1%
Bilal AI et al., 2016	525	636	0.825	[0.794; 0.854]	1.1%
Atif M et al., 2016	1236	2400	0.515	[0.495; 0.535]	1.1%
Akhtar M et al., 2012	245	302	0.811	[0.762; 0.854]	1.1%
Henry CN et al., 2013	212	600	0.353	[0.315; 0.393]	1.1%
Angamo MT et al., 2011	1645	3058	0.538	[0.520; 0.556]	1.1%
Al−Niemat SI et al., 2014	3840	4530	0.848	[0.837; 0.858]	1.1%
Mohajer KA et al., 2011	822	4433	0.185	[0.174; 0.197]	1.1%
Wang H et al., 2013	1525	5099	0.299	[0.287; 0.312]	1.1%
Mulwa Charles N et al., 2015	525	720	0.729	[0.695; 0.761]	1.1%
Mudenda W et al., 2016	426	793	0.537	[0.502; 0.572]	1.1%
Joda AE et al., 2013	403	933	0.432	[0.400; 0.464]	1.1%
Assen A et al., 2014	191	362	0.528	[0.475; 0.580]	1.1%
Fadare J et al., 2015	374	526	0.711	[0.670; 0.749]	1.1%
Muyu G et al., 2013	266	391	0.680	[0.632; 0.726]	1.1%
Desalegn AA et al., 2013	749	1290	0.581	[0.553; 0.608]	1.1%
Pathak A et al., 2011	3732	5623	0.664	[0.651; 0.676]	1.1%
Kolyva S et al., 2017	382	2416	0.158	[0.144; 0.173]	1.1%
Sharma S et al., 2016	299	811	0.369	[0.335; 0.403]	1.1%
Wushouer H et al., 2020	103321	385529	0.268	[0.267; 0.269]	1.1%
Okoro RN et al., 2019	211	802	0.263	[0.233; 0.295]	1.1%
Cole CP et al., 2018	488	600	0.813	[0.780; 0.844]	1.1%
Mao W et al., 2019	1293	3878	0.333	[0.319; 0.349]	1.1%
Mao W et al., 2019	1130	3802	0.297	[0.283; 0.312]	1.1%
Palms DL et al., 2018	1722927	7500000	0.230	[0.229; 0.230]	1.1%
Al−Azayzih A et al., 2017	4269	24089	0.177	[0.172; 0.182]	1.1%
Ahmed AM et al., 2010	488	600	0.813	[0.780; 0.844]	1.1%
Park SY et al., 2022	1843	46314	0.040	[0.038; 0.042]	1.1%
Yang Q et al., 2022	42661	201963	0.211	[0.209; 0.213]	1.1%
Yang Q et al., 2022	88970	406000	0.219	[0.218; 0.220]	1.1%
Yang Q et al., 2022	89068	416271	0.214	[0.213; 0.215]	1.1%
Yang Q et al., 2022	88372	431701	0.205	[0.204; 0.206]	1.1%
Oghuvwu S et al., 2023	658	2400	0.274	[0.256; 0.292]	1.1%
Ranasinghe P et al., 2022	1327	3055	0.434	[0.417; 0.452]	1.1%
Joshi R et al., 2022	853	4838	0.176	[0.166; 0.187]	1.1%
Okoye BI et al., 2022	651	800	0.814	[0.785; 0.840]	1.1%
El−Dahiyat F et al., 2022	180	419	0.430	[0.382; 0.479]	1.1%
Sodhi B et al., 2022	144	219	0.658	[0.591; 0.720]	1.1%
Amponsah OKO et al., 2022	39339	110280	0.357	[0.354; 0.360]	1.1%
Yimer YS et al., 2022	276	780	0.354	[0.320; 0.389]	1.1%
Meenakshi R et al., 2022	44	600	0.073	[0.054; 0.097]	1.1%
Abu Farha R et al., 2022	207	2451	0.084	[0.074; 0.096]	1.1%
Avci IY et al., 2006	5004	33491	0.149	[0.146; 0.153]	1.1%
Al−Niemat SI et al., 2008	66865	187822	0.356	[0.354; 0.358]	1.1%
Akande TM et al., 2007	135	303	0.446	[0.389; 0.503]	1.1%
Oshikoya KA et al., 2007	804	1944	0.414	[0.392; 0.436]	1.1%
Dimri S et al., 2009	74	254	0.291	[0.236; 0.351]	1.1%
Ghimire S et al., 2009	1196	4231	0.283	[0.269; 0.297]	1.1%
Karande S et al., 2005	198	500	0.396	[0.353; 0.440]	1.1%
Chukwuani CM et al., 2002	151	300	0.503	[0.445; 0.561]	1.1%
**Random effects model**		**184735601**	**0.343**	**[0.296; 0.393]**	**100.0%**

Heterogeneity: I^2 = 99.99%, τ^2 = 0.0596, P < 0.001

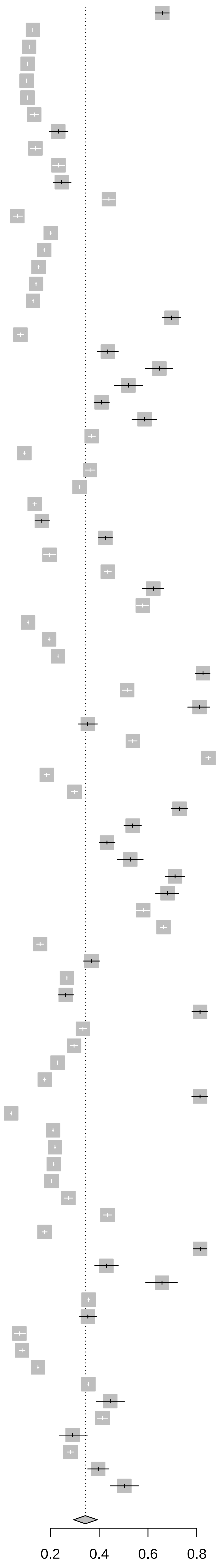

Figure S1. Forest plot of outpatient antibiotic use

Supplementary Figure 2. Outpatient antibiotic prescribing across countries

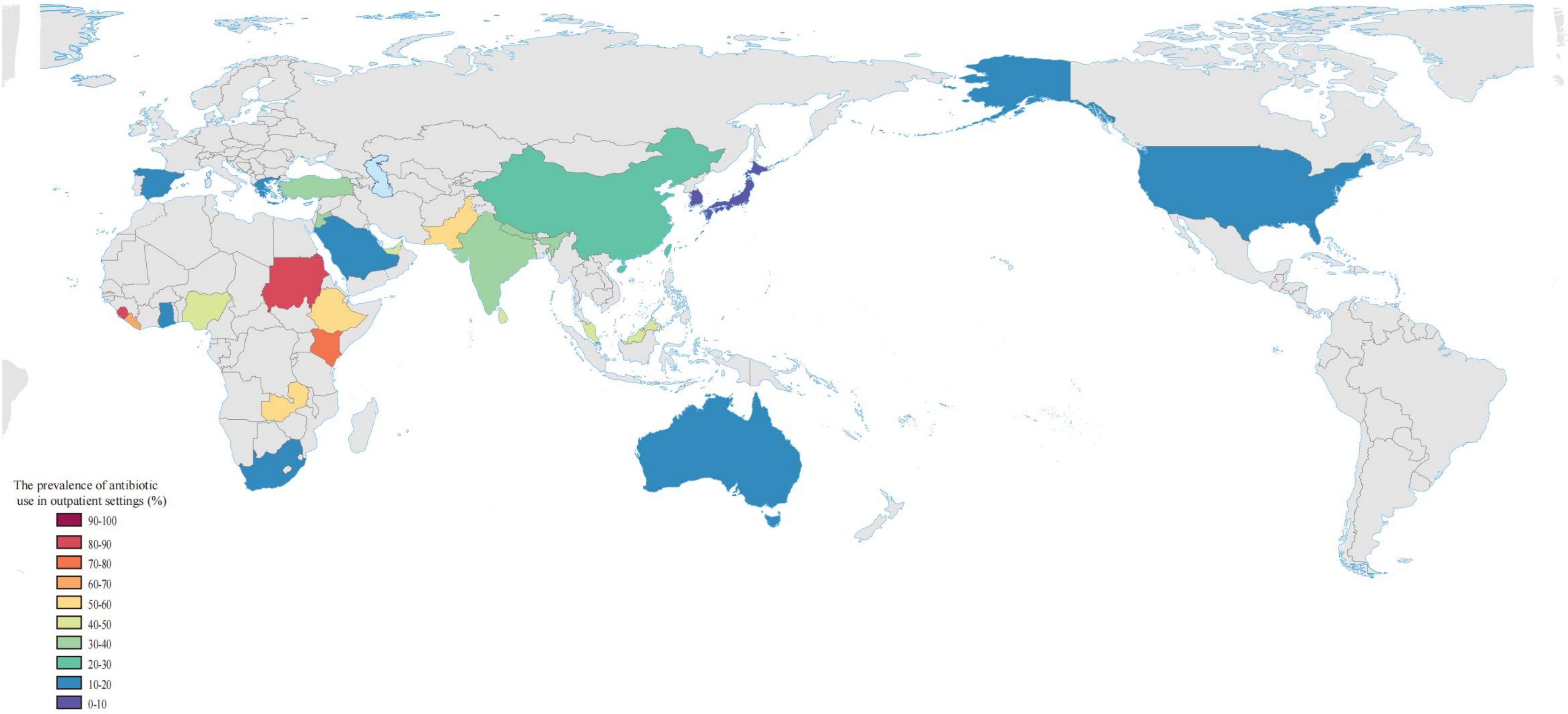

Figure S2. Outpatient antibiotic prescribing across countries

Supplementary Figure 3. Forest plot of inpatient antibiotic prescribing

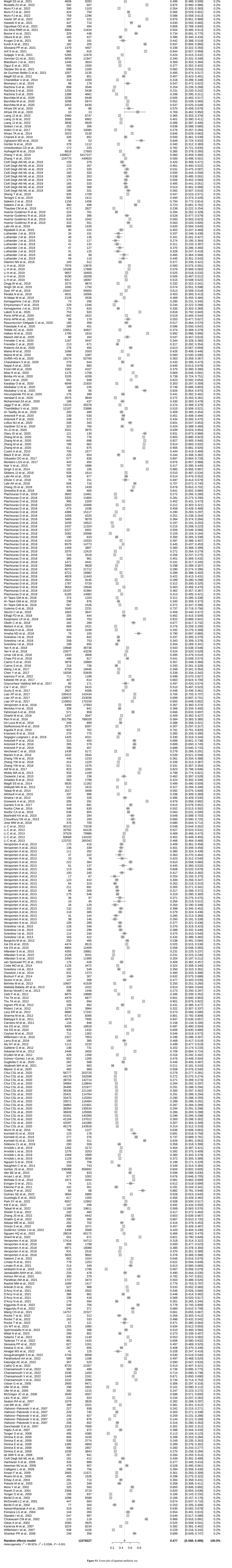

Figure S3. Forest plot of inpatient antibiotic use

Supplementary Figure 4. Year-prevalence scatter plot

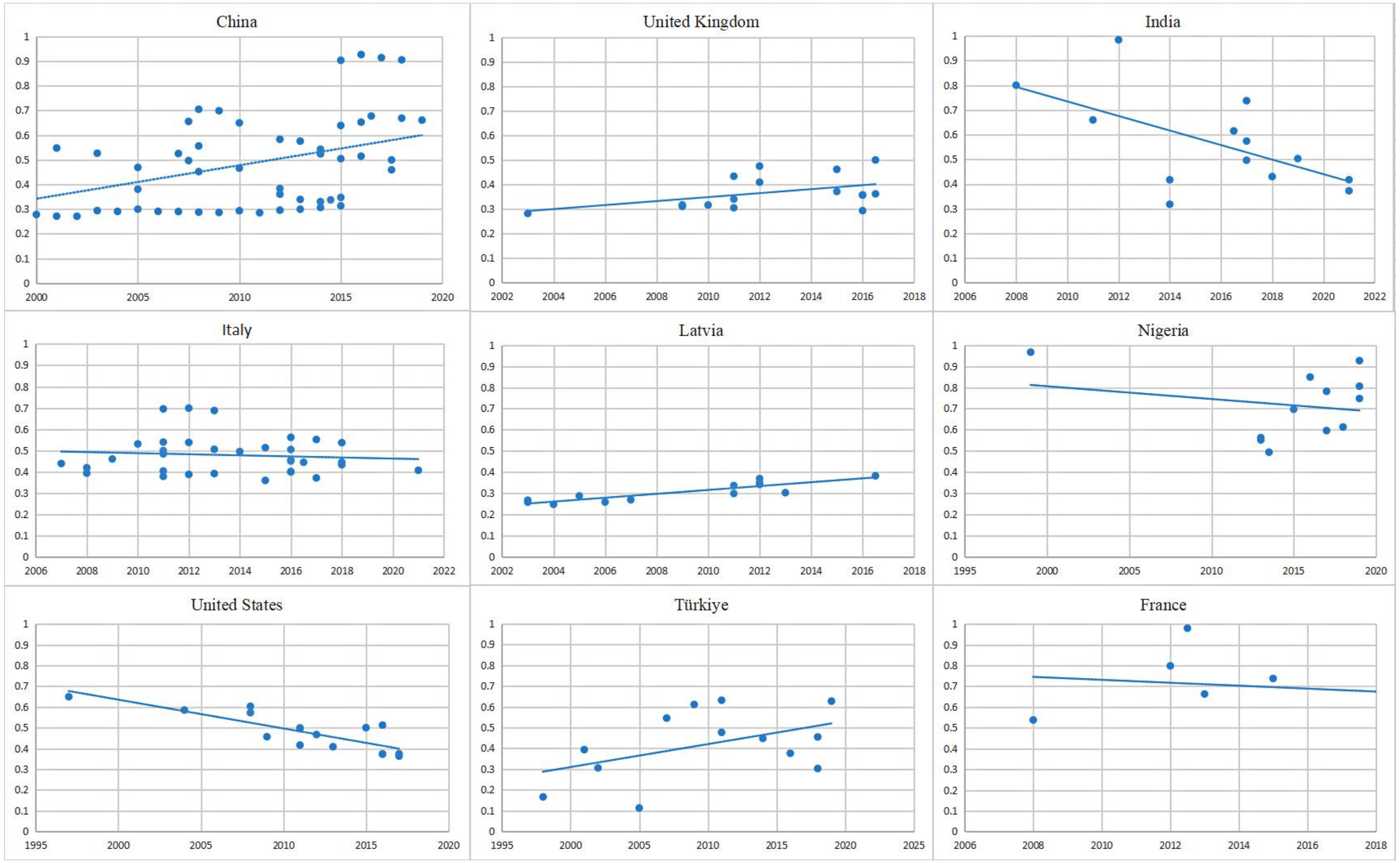

Figure S4. Year-prevalence scatter plot
